# “We were locked in with our trauma” - a mixed-methods study of health pathways among intimate partner violence (IPV) survivors during COVID-19 lockdowns in Ontario

**DOI:** 10.1186/s12889-026-28032-6

**Published:** 2026-06-19

**Authors:** Dina Idriss-Wheeler, Ziad El-Khatib, Lauren Hancock, Marlene Ham, Shannon Bainbridge, Raywat Deonandan

**Affiliations:** 1 Interdisciplinary School of Health Sciences, University of Ottawa, Ottawa, Canada; 2 Department of Global Public Health, Karolinska Institutet, Stockholm, Sweden; 3 Ontario Association of Interval and Transition Houses, Toronto, Canada; 4 Department of Cellular and Molecular Medicine, University of Ottawa, Ottawa, Canada

**Keywords:** intimate partner violence (IPV), COVID-19 pandemic, women's health, mental health, physical health, behavioural pathways, mixed methods, emergency preparedness

## Abstract

**Background:**

The COVID-19 pandemic disproportionately affected certain populations, with intimate partner violence (IPV) survivors facing heightened risks of adverse health outcomes. This study examined whether women who experienced IPV during Ontario’s COVID-19 lockdowns were more likely to report negative behavioural, psychological, and physiological health outcomes compared to women who did not experience IPV .

**Methods:**

Using a convergent mixed-methods design, a cross-sectional survey of Ontario residents and semi-structured interviews with key stakeholders were conducted. The final sample comprised 653 survey respondents who identified as women, 23% of whom reported experiencing IPV during COVID-19 lockdowns (*n* = 150). A total of 24 interviews were conducted, including 14 IPV survivors and 10 violence against women (VAW) service providers.

**Results:**

Quantitative findings revealed that IPV survivors had significantly higher odds of adverse behavioural outcomes, including increased alcohol (43.3% vs. 21.9%, RRR = 3.39, *p* < 0.001), tobacco (15.3% vs. 8.8%, RRR = 2.16, *p* = 0.014), cannabis (23.8% vs. 10.4%, RRR = 3.49, *p* < 0.001), and illicit substance use (6.8% vs. 0.6%, RRR = 14.31, *p* < 0.001), as well as greater decreases in sleep quality (61.4% vs. 40.3%, RRR = 3.24, *p* < 0.001). They also exhibited more polarized help-seeking patterns: increased reliance on formal supports (34.7% vs. 12.9%, RRR = 4.75, *p* < 0.001) and higher likelihood of decreased informal support (29.5% vs. 17.3%, RRR = 2.53, *p* = 0.001). Logistic regression further supported that IPV survivors had over twice the odds of reporting poor mental health (aOR = 2.46, 95% CI: 1.27–4.75) and nearly twice the odds of poor physical health (aOR = 1.84, 95% CI: 1.00–3.36). Qualitative insights revealed that structural barriers, trauma, stigma, and loss of autonomy contributed to these adverse outcomes.

**Conclusions:**

These results underscore the bidirectional relationship between mental and physical health and the compounded vulnerabilities exacerbated by crisis conditions. Findings highlight the need to integrate IPV considerations into emergency preparedness and response planning to ensure continuity of care for IPV survivors. As public health emergencies and climate disasters become more frequent, inclusive responses must prioritize those most at risk, including IPV survivors.

**Supplementary Information:**

The online version contains supplementary material available at 10.1186/s12889-026-28032-6.

## Background

Intimate partner violence (IPV) - including physical, psychological/emotional, sexual, technological, spiritual and financial abuse - is recognized as both a social justice and human rights issue [1]. IPV is situated within the broader continuum of gender-based violence (GBV), which encompasses a range of harms rooted in gender inequality and power imbalances [2]. Global bodies, such as the World Health Organization (WHO) and the United Nations (UN) Women have called for urgent, equity-focused action to address its health impacts [3, 4]. In Canada, organizations like the Canadian Women’s Foundation and the Women’s Shelters Canada have advocated for systemic, trauma-informed responses that center survivors’ dignity and rights [5].

Approximately 44% of Canadian women report experiencing some form of IPV in their lifetime, with higher rates among Indigenous, racialized, and younger women [2, 6, 7]. IPV is associated with negative physical and mental health outcomes across the lifespan [1, 8]. These health impacts can become even more pronounced during stressful life events (SLEs), such as public health crises, economic recessions, and environmental disasters [9]. This includes the global COVID-19 pandemic, during which mandated lockdowns intensified IPV severity, increased survivors’ isolation, and reduced access to health and social support systems globally [9]. While these disruptions affect the general population, IPV survivors tend to face disproportionate barriers to accessing health and social supports, further increasing their risk of poor health outcomes.

In Canada, studies have reported increased risk of IPV and worsened health and wellbeing among survivors during COVID-19 lockdowns [9–13]. While national and international research has documented adverse health outcomes among IPV survivors during the pandemic [9], gaps remain in understanding how lockdowns disrupted multiple dimensions of health simultaneously.

This study is guided by the socioecological model to explore multilevel influences on health [14]. It also draws on Berkman and Krishna’s (2014) conceptualization of health pathways: (i) behavioural (e.g., substance use, sleep, diet, exercise, help-seeking), (ii) psychological (e.g., mental health, self-efficacy, coping, emotional regulation), and (iii) physiological (e.g., physical health, stress response, immune function) [15]. These frameworks allow for an in-depth analysis of how COVID-19 lockdowns disrupted multiple health pathways for IPV survivors and intensified pre-existing health vulnerabilities. To the authors’ knowledge, no study to date has concurrently examined perceived health outcomes across three key health pathways - behavioural, psychological, and physiological - while integrating both survivors’ lived experiences and the perspectives of service providers.

Conducted in Ontario, Canada, our study examines whether women who experienced IPV during the COVID-19 lockdowns were at greater risk of adverse behavioural, psychological, and physiological health outcomes compared to women who did not experience IPV. Ontario experienced some of the longest lockdown periods in Canada, beginning in March 2020 and intermittently extending until June 2021, with restrictions including school closures, stay-at-home orders, and limited access to services [16]. Quantitative analyses compared self-reported health data between IPV and non-IPV groups, while qualitative interviews with IPV survivors and violence against women (VAW) service providers offered contextual insights into structural barriers and contributing mechanisms.

We hypothesized that although lockdown measures affected the population broadly, IPV survivors experienced more severe disruptions across all health pathways, resulting in worse overall health outcomes than the non-IPV women. By documenting the disproportionate health impacts of public health emergencies on IPV survivors, this contributes evidence for more equitable and responsive emergency preparedness and planning.

## Methods

### Study design and setting

We employed a convergent mixed-methods approach [17], collecting quantitative and qualitative data simultaneously, analyzing them independently, and integrated findings for comprehensive interpretation. The study received approval from the University of Ottawa Research Ethics Board (H-01-22-7703; February 8, 2022). We adhered to the Good Reporting of a Mixed Methods Study (GRAMMS) checklist to ensure comprehensive and transparent reporting of our design and findings (Additional File 1) [18]. Conducted in Ontario, Canada’s most populous province, the study used a cross-sectional survey to compare health pathways between IPV and non-IPV participants, complemented by in-depth interviews exploring survivors’ lived experiences and service providers’ insights. This mixed-methods design aimed to triangulate findings and enhance understanding of how the COVID-19 lockdowns affected health among IPV survivors.

### Partnership and advisory group

A partnership was established with the Ontario Association of Interval and Transition Houses (OAITH), a provincial community-based organization supporting IPV survivors. A project advisory group (PAG) was convened, consisting of the principal investigator (DIW), OAITH’s executive director and policy/research coordinator, a program manager from the Salvation Army Family Life Resource Centre in Brampton, and when available, a volunteer IPV survivor. The PAG contributed to study design, tool development, and data interpretation.

### Instrument development

#### Quantitative component

An online survey was developed using validated instruments, findings from a prior scoping review, and feedback from the PAG [9, 19–26]. The PAG ensured content and face validity, cultural relevance, and facilitated pretesting among IPV survivors and service providers. Revisions to the survey were made based on this feedback. The instrument was initially tested with 6 participants. Following revisions, it was re-administered to 12 participants, 4 of whom also completed the first round. This allowed for a preliminary assessment of test–retest reliability, while also expanding the sample for further validation.

#### Qualitative component

The semi-structured interview guides were developed specifically for this study to explore experiences of IPV and health pathways during COVID-19 lockdowns (see Additional File 2). The guides were informed by literature and refined in collaboration with the PAG for clarity and cultural sensitivity. Pre-tests were conducted with subject-matter experts and community members (i.e., survivors and service providers), with subsequent revisions based on feedback.

### Recruitment

#### Survey and interview recruitment was carried out concurrently

##### Quantitative component

Survey recruitment took place from February to April 2024, through a survey research firm, Leger LEO. A quota-sampling [27] design was implemented to ensure demographic representativeness across Ontario, with quotas set on the basis of gender (approximately 50% female, 50% male) and age group (18–24, 25–34, 35–44, 45–54, 55–64, 65–74, and 75+), reflecting Ontario’s population distribution. The sampling frame consisted of 27,184 panel members; 26,490 were invited to participate, of whom 3,072 opened the survey. A total of 1,344 of the opened surveys were completed, with 1,480 participants screened out for failing to meet eligibility criteria and 248 excluded due to incomplete responses. All participants provided informed consent prior to participation. Inclusion criteria were: Ontario residents aged 18 years or older who consented to participate. Exclusion criteria were: individuals residing outside Ontario, those under 18 years of age, and those who did not provide informed consent. For this mixed-methods analysis, the analytic sample was further restricted to the 653 respondents who self-identified as women, as only women IPV survivors participated in the qualitative component.

##### Qualitative component

Semi-structured interview recruitment occurred concurrently with the survey, from October 2023 to March 2024. Participants were recruited through convenience sampling, facilitated by the PAG and its networks of VAW organizations across Ontario. Recruitment materials were circulated through OAITH and partner organizations to reach both IPV survivors and VAW service providers. Interviews were conducted via secure online platforms (Microsoft Teams or Zoom) or in person, based on participant preference, and participants received compensation for their time. Inclusion criteria for IPV survivor interviews were: self-identified survivors of IPV aged 18 years or older; residing in Ontario during the COVID-19 lockdown period (March 2020–June 2021); and able to participate in English or French. Inclusion criteria for VAW service provider interviews were: currently or recently employed in a VAW or IPV-related service organization in Ontario with direct experience providing services during the COVID-19 lockdown period; and able to participate in English or French. Exclusion criteria for both groups were: individuals who did not meet the above criteria, those unable or unwilling to provide informed consent, and those for whom participation would have posed a safety risk.

##### Sample

We surveyed 1,344 Ontario residents aged *≥* 18, including 653 women, 661 men, 12 gender-diverse participants, and 18 who preferred not to disclose their gender. Because only women IPV survivors participated in the qualitative interviews, the mixed-methods analysis was restricted to the 653 women survey respondents. For the qualitative arm, we conducted 24 semi-structured interviews: 14 with women who identified as IPV survivors and 10 with violence against women (VAW) service providers. All interviews were audio-recorded, transcribed, and supplemented by field notes and reflexive journaling.

For the purposes of this study, participants were categorized based on self-reported IPV experience during the COVID-19 lockdown period. *“Women with IPV experience”* refers to participants who reported any form of IPV during this time, while *“women without IPV experience”* refers to those who did not. For brevity in tables and comparative analyses, these groups are referred to as *“yes IPV”* and *“no IPV*,*”* respectively. In narrative and qualitative sections, the term *“IPV survivors”* is also used to recognize the lived experiences and resilience of those affected.

While no formal power analysis was conducted due to the exploratory nature of the study, our quantitative sample of 653 women provided sufficient variability to detect meaningful differences in health outcomes between yes IPV and no IPV groups. For the qualitative component, the sample size (*n* = 24) encompassed both IPV survivor and provider perspectives. Adequacy was not framed in terms of “data saturation,” as this concept has been critiqued by Clarke and Braun (2021) as incompatible with reflexive thematic analysis [28, 29]. Instead, it was assessed based on whether the dataset was sufficiently rich and relevant to address the study’s objectives. Following the principle of “information power”, discussed by Malterud et al.(2016), the diversity and specificity of participants were judged to provide adequate insight to generate robust and meaningful themes [30].

##### Variable selection and measurement

We measured changes in behavioural, psychological, and physiological health pathways using self-reported survey data. Participants indicated whether each health domain had increased, decreased, or remained the same during the COVID-19 lockdown period.

*Behavioural pathways* included alcohol consumption, tobacco use, cannabis use, illicit substance use, screen time (TV), internet use, physical exercise, junk food consumption, and sleep quality. For each domain, participants were asked: *“Did you change your weekly habits for any of the following activities during COVID-19 lockdowns?”* with response options of increased, decreased, or no change. The same format was applied consistently across all behavioural domains. Increases in substance use, sedentary behaviour, and unhealthy eating, as well as decreases in sleep quality and physical activity, are associated with negative health outcomes [31–33].

In qualitative interviews, IPV Survivors were asked: “*Can you tell me a story or talk about any changes in health behaviours – did you find you exercised less or more*,* slept less*,* ate more junk*,* drank more alcohol or smoked more during covid-19?”* Service providers were asked: *“From your perspective*,* can you comment or provide a story regarding changes in health behaviours of clients due to COVID-19 lockdowns?”*

*Psychological pathways* were assessed through participants’ perceived changes in mental health relative to pre-pandemic. Participants were first asked to rate their mental health before the lockdowns: *“Would you say*,* in general*,* that your mental health — which includes stress*,* depression*,* anxiety*,* and problems with emotions — before COVID-19 lockdowns (March 2020–June 2021) was:”* (response options: excellent, very good, good, fair, or poor). They were then asked: *“Thinking about your overall mental health — which includes stress*,* depression*,* anxiety*,* and problems with emotions — how did COVID-19 lockdowns affect your mental health? My mental health became…”* (response options: much better, better, about the same, worse, or much worse). These two items were combined to derive a binary outcome: poor mental health (coded as 1) was assigned to participants who reported their mental health was worse or much worse during lockdowns, or who rated it as about the same but had reported poor mental health pre-pandemic. All other combinations were coded as good mental health (0). Full coding details are provided in Additional File 3.

In qualitative interviews, IPV survivors were asked: *“Can you talk about or provide a story regarding change in your mental health (e.g.*,* coping*,* depression/distress*,* emotional regulation*,* sense of well-being*,* PTSD)? What was tough for you*,* and how did you find it mentally to deal with things during this time?”* Service providers were asked: “*From your perspective*,* can you comment on or provide a story regarding change in your clients’ overall mental health?*”

*Physiological pathways* were measured through participants’ perceived changes in physical health relative to pre-pandemic. Participants were first asked to rate their physical health before the lockdowns: *“Would you say*,* in general*,* that your physical health — which includes illness and injury — before COVID-19 lockdowns (March 2020–June 2021) was:”* (response options: excellent, very good, good, fair, or poor). They were then asked: *“Thinking about your overall physical health — which includes illness and injury — how did COVID-19 lockdowns affect your physical health? My physical health became…”* (response options: much better, better, about the same, worse, or much worse). These two items were combined to derive a binary outcome: poor physical health (coded as 1) was assigned to participants who reported their physical health was worse or much worse during lockdowns, or who rated it as about the same but had reported poor physical health pre-pandemic. All other combinations were coded as good physical health (0). Full coding details are provided in Additional File 3.

In qualitative interviews, IPV Survivors were asked: “*Can you talk about or provide a story regarding change in your physical health (stress overload*,* immunity/getting sick more often*,* physical injury)?”* Service providers were asked: “*From your perspective*,* can you comment on or provide a story regarding change in your clients’ overall physical health?*”

*Main independent variable (IPV experience)*: The primary independent variable was self-reported exposure to IPV during the COVID-19 lockdown period (March 2020–June 2021), coded dichotomously as “Yes IPV” or “No IPV.” IPV was assessed across multiple forms, including physical, emotional/psychological, sexual, financial, technological, and spiritual/religious abuse by a current or former partner. Participants who reported experiencing any form of abuse were directed to complete additional items based on the validated Composite Abuse Scale (Revised) – Short Form (CASR-SF) [34], providing further validation of self-reported IPV experience. Respondents were classified as “Yes IPV” if they reported any form of abuse during the specified lockdown period, including those who had separated from their partner but continued to experience IPV post-separation. This measurement approach has been described in full in a recently published study using the same dataset [35].

*Control variables*: Sociodemographic variables were included based on national data collection standards and Canadian Institute for Health Information (CIHI) guidelines [36–39]. These included: gender, education level, immigration status, Racial and Indigenous identity, age (18–24, 25–34, 35–44, 45–54, 55–64, and 65+), geographic region (Eastern, Central, Toronto, Suburban “905” belt, Western, and Northern), household income level (< CAD$40,000; CAD$40,000– CAD $69,999; CAD $70,000– CAD $99,999; and CAD $100,000+), and employment status during the COVID-19 lockdowns (self and partner).

*Covariates*: Additional variables were selected based on prior literature [40–49] and stakeholder input due to their established associations with health outcomes:

- Community violence exposure (yes/no).
- Informal caregiving responsibilities, including: Change in caregiving load, primary caregiver status (yes/no), number of dependents.
- Service information access: Whether participants felt informed about service availability (yes/no).
- Substance use impact score: A composite score capturing stress related to partner substance use. Response items were dichotomized (always / often / sometimes = 1; never / seldom = 0), summed, and categorized as “low impact” (*≤* 2) or “moderate / high impact” (> 2).

##### Multinomial Logistic regression

*Outcome variables (health behaviors)*: For each health behavior domain, outcome variables were recoded into three mutually exclusive categories based on participant self-report during the COVID-19 lockdown period (March 2020-June 2021):

- Increased (coded as 1): Assigned to participants who reported a greater frequency or intensity of the behavior compared to pre-pandemic.
- Decreased (coded as 2): Assigned to participants who reported a lower frequency or intensity of the behavior compared to pre-pandemic.
- No change (coded as 0): Assigned to participants who reported no change in the behavior. This category was used as the reference outcome in all multinomial regression models.

The following domains were included: alcohol consumption, tobacco use, cannabis use, illicit substance use, television watching, internet use, physical activity, junk food consumption, sleep quality, and informal and formal supports help seeking.

##### Multivariate logistic regression

*Outcome variables (poor mental and poor physical health)*: Two binary outcome variables were created based on self-reported health status [50, 51].

- Poor mental health (coded as 1): Assigned to participants who rated their mental health during the COVID-19 period (March 2020 – June 2021) as “worse or “much worse”, or who rated it as “about the same” but had reported “poor” mental health pre-pandemic.
- Good mental health (coded as 0): Assigned to participants with all other combinations.
- Poor physical health (coded as 1): Assigned using the same logic applied to self-reported mental health status.
- Good physical health (coded as 0): Assigned to participants with all other combinations.

Participants with missing data for either time point (pre-pandemic or during lockdown) were excluded from analyses. Additional details on variable coding and survey items are provided in Additional File 3; STATA Syntax available in Additional File 4.

*Qualitative contextualization*: In interviews, survivors elaborated on how IPV affected their mental and physical health, as well as health-related behaviours before and during the pandemic. Service providers reflected on changes in client needs, behaviours and access barriers during Ontario’s lockdowns, offering insight into the mechanisms behind the quantitative findings.

##### Data analysis

*Quantitative analysis*: Categorical variables were summarized using proportions. Bivariate relationships between IPV status and behavioural or health outcomes were assessed using Chi-square (or Fisher’s exact) tests with Bonferroni correction (Additional File 5). For health behavior outcomes, multinomial logistic regression models were estimated to assess differences in behaviour pathways change (increased or decreased vs. no change) between women with and without IPV experience, adjusting for covariates/controls. Multivariate logistic regression models were constructed for self-reported poor mental health and poor physical health outcomes, adjusting for relevant covariates. Analyses were conducted using the software package STATA version 18 [52].

*Qualitative analysis*: Interviews were audio-recorded with consent, transcribed verbatim, and supplemented with field notes and reflexive memos. Data analysis followed the six phases of Reflexive Thematic Analysis as outlined by Braun and Clarke (2006, 2022), using both inductive (to identify emergent patterns) and deductive (individual, community, policy) coding. Separate codebooks were maintained for IPV survivor and VAW service provider data. NVivo 12 software was used to manage data from service provider interviews, while Dedoose was used for survivor interview data, reflecting the co-analyzer’s proficiency and preference with the respective platforms. Coding reports related to health outcomes and health pathways of IPV survivors were the focus of this analysis. Themes were reviewed and refined with the PAG for relevance and alignment. Reflexivity was prioritized throughout the process via memoing before and after each interview, alongside ongoing team-based reflection.

*Integrated data analysis*: Following a convergent parallel approach, quantitative and qualitative data were analyzed independently before being merged for integrated interpretation [17]. Quantitative findings were organized by health pathway — behavioural, psychological, and physiological — and these pathways served as the organizing framework for integration. Qualitative themes that answered the key questions about health pathways were then systematically mapped onto each quantitative pathway outcome to identify points of convergence (where qualitative findings confirmed quantitative results), divergence (where findings conflicted or complicated each other), and expansion (where qualitative data explained mechanisms that the survey could not capture). For example, the quantitative finding that IPV survivors were significantly more likely to report decreased internet use — counterintuitive during a period of broadly increased internet usage — was explained through qualitative accounts of technological abuse and coercive control, representing a point of expansion. Where qualitative data neither confirmed nor contradicted a quantitative finding — such as the association between male partners and poor mental health — this non-alignment was explicitly noted rather than forced into convergence. Interpretations emerging from this integration process were reviewed with the PAC to ensure community relevance and alignment with survivor and service provider perspectives.

## Results

### Sample characteristics

Table 1 summarizes the descriptive statistics of survey respondents who experienced IPV with those who did not during the COVID-19 lockdowns in Ontario (see Additional File 5– Table S1 for the full descriptive table). Of the 653 women surveyed, 23% (*n* = 150) reported experiencing IPV during the lockdown period. Significant differences emerged across sociodemographic and contextual factors. IPV survivors were more likely to be aged 18–34 (51.33% vs. 20.12%), while non-IPV women were older and aged 55+ (38.25% vs. 7.33%). A higher proportion of IPV survivors identified as racialized or Indigenous (42.00% vs. 16.30%), LGBTQ2+ (19.46% vs. 6.96%) and had household incomes below $40,000 (41.55% vs. 14.44%). Differences were also observed in partner characteristics. Survivors were more likely to have racialized partners (37.58% vs. 17.37%), younger partners aged 18–34 (46.94% vs. 16.80%), and partners with only primary education (33.78% vs. 25.65%). Geographically, a greater proportion of IPV survivors resided in Eastern Ontario (19.33% vs. 10.34%). They also reported community violence as a problem (42.00% vs. 27.35%), were more often informal caregivers (60.00% vs. 44.53%), and were less likely to feel adequately informed about available services during the pandemic (46.98% vs. 70.82%).

**Table 1:** Summary of Descriptive Statistics of Ontario Survey Respondents who Experienced Intimate Partner Violence (IPV) and those who did not During COVID-19 Lockdowns (*n* = 653)

Variable	Yes IPV (%) (*n* = 150; 23%)	No IPV (%) (*n* = 503; 77%)	*p*-value
Age 18–34	51.3%	20.1%	< 0.001
Racialized/Indigenous identity	42.0%	16.3%	< 0.001
LGBTQ2 + identity	19.5%	7.0%	< 0.001
Household income < $40K	41.6%	14.4%	< 0.001
Enough info about services	47.0%	70.8%	< 0.001
Moderate/High impact of substance use	62.0%	10.5%	< 0.001
Has children	54.7%	34.3%	< 0.001
Informal caregiver	60.0%	44.5%	0.001
Perceives community violence as a problem	42.0%	27.4%	0.001
Partner racialized/Indigenous	37.6%	17.4%	< 0.001
Partner unemployed	36.5%	29.3%	0.095
Geographic differences	Higher in Eastern Ontario	Lower in Western Ontario	0.007

Table 2 summarizes the descriptive statistics of interview participants who experienced IPV and those who were service providers. Among the 14 IPV survivors interviewed, the average age was 37 years (range: 25–59), with all identifying as female. Half of the survivors were racialized or Indigenous, and the majority (86%) lived in urban areas. For the 10 service providers, all were female, 30% were racialized or Indigenous, and 70% worked in urban areas.

**Table 2:** Interview Participant characteristics

Characteristics*	IPV Survivors (*n* = 14)	Service Providers (*n* = 10)
Average age	37 years old (min = 25, max = 59)	N/A
Gender		
Female	14 (100%)	10 (100%)
Race		
Racialized/Indigenous	7 (50%)	3 (30%)
White	7 (50%)	7 (70%)
Partner Race		
Racialized/Indigenous	8 (57%)	N/A
White	6 (43%)	
Urban/rural		
Urban	12 (86%)	7 (70%)
Rural	2 (14%)	3 (30%)
Geography**		
Eastern Ontario	2 (14%)	3 (30%)
Central Ontario	5 (36%)	3 (30%)
Toronto and the GTA (905 Belt)	4 (29%)	2 (20%)
Western Ontario	2 (14%)	2 (20%)
Northern Ontario	1 (7%)	-
Informal Caregiver		
Yes	10 (71%)	N/A
No	4 (29%)	
Discussed Financial constraints***	14 (100%)	N/A

* Participant characteristics were collected during interviews and were not part of the structured questionnaire

** For a map of Ontario’s geographical regions, refer to this link on the Government of Ontario website. [53]

***Although not directly asked,* all participants voluntarily discussed financial constraints during theinterviews. These included challenges such as lack of affordable housing after leaving transition housing/shelter*,* returning to live with family post abuse*,* having to share accommodations with others*,* having difficulties making ends meet*,* and experiences of financial abuse by a partner*

### Health pathways

#### Behavioural

Table 3 summarizes multinomial logistic regression estimates of changes in health behaviors, highlighting differences between women with and without IPV experience during the COVID-19 lockdowns. Significant differences were observed across most behavioural domains, with yes IPV consistently reporting greater changes compared to no IPV. In contrast, no IPV respondents more frequently reported no change across behaviours—a pattern evident across nearly all domains (Refer to Additional File 5 – Table S2 for descriptives).

**Table 3:** Multinomial Logistic Regression Results: Relative Risk Ratios (RRR), Model-Adjusted Predicted Probabilities (PP), and Differences in Outcomes Between IPV and Non-IPV Groups (Reference = ‘No Change’)

Domain / Outcome	Change vs. “No change”	RRR (Yes IPV vs. No IPV)	*p*-value	PP No IPV (%)	PP Yes IPV (%)	Difference (%)	*p*-value
Alcohol	Increased	3.39 (2.06–5.58)	0.001	21.90	43.30	21.40	0.001
	Decreased	2.56 (1.33–4.96)	0.005	9.90	14.70	4.80	0.161
	No change (base)	–	-	68.20	42.00	-26.20	0.001
Tobacco	Increased	2.16 (1.17–4.00)	0.014	8.80	15.30	6.50	0.045
	Decreased	3.21 (1.20–8.61)	0.021	3.00	7.90	4.90	0.083
	No change (base)	–		88.20	76.80	-11.40	0.005
Cannabis	Increased	3.49 (2.00–6.10)	0.001	10.40	23.80	13.40	0.001
	Decreased	8.16 (2.81–23.70)	0.001	1.70	9.10	7.40	0.009
	No change (base)	–		87.90	67.10	-20.80	0.001
Illicit substances	Increased	14.31 (3.54–57.89)	0.001	0.60	6.80	6.20	0.001
	Decreased	4.50 (1.18–17.09)	0.027	1.20	4.10	2.90	0.078
	No change (base)	–		98.20	89.10	-9.10	0.001
TV watching	Increased	1.00 (0.56–1.78)	0.992	72.90	70.80	-2.10	0.691
	Decreased	1.53 (0.59–3.97)	0.378	5.80	8.30	2.50	0.338
	No change (base)	–		21.40	20.90	-0.50	0.927
Internet use	Increased	1.28 (0.70–2.37)	0.422	77.20	76.90	-0.30	0.956
	Decreased	3.56 (1.28–9.89)	0.015	2.90	7.50	4.60	0.039
	No change (base)	–		20.00	15.60	-4.40	0.298
Exercise	Increased	1.37 (0.76–2.47)	0.303	26.30	26.30	0.00	0.999
	Decreased	1.73 (1.05–2.87)	0.032	36.70	46.40	9.70	0.068
	No change (base)	–		36.90	27.30	-9.60	0.045
Junk food	Increased	1.19 (0.74–1.93)	0.47	46.40	48.10	1.70	0.750
	Decreased	1.55 (0.74–3.26)	0.244	9.90	13.20	3.30	0.360
	No change (base)	–		43.80	38.70	-5.10	0.340
Informal support	Increased	1.76 (1.01–3.07)	0.045	22.60	27.20	4.60	0.330
	Decreased	2.53 (1.47–4.36)	0.001	17.30	29.50	12.20	0.008
	No change (base)	–		60.10	43.30	-16.80	0.002
Formal support	Increased	4.75 (2.74–8.24)	0.001	12.90	34.70	21.80	0.001
	Decreased	2.98 (1.68–5.29)	0.001	13.70	22.30	8.60	0.034
	No change (base)	–		73.40	43.00	-30.40	0.001
Sleep quality	Increased	2.19 (1.12–4.29)	0.022	16.50	16.90	0.40	0.078
	Decreased	3.24 (1.87–5.62)	0.001	40.30	61.40	21.10	0.000
	No change (base)	–		43.20	21.70	-21.50	0.000

*RRR* = relative risk ratio; *PP* = predicted probability; *CI* = confidence interval. Reference category = “No Change.” All models adjusted for sociodemographic and contextual covariates

Substance use disparities were evident across all four categories examined. Survivors faced significantly elevated relative risks of increased substance use compared to those with no IPV exposure. Survivors had more than three times the relative risk of reporting increased alcohol use compared to the no change reference category (RRR = 3.39, 95% CI: 2.06–5.58, p < 0.001), with predicted probabilities of 43.3% versus 21.9% for non-IPV respondents. Similar patterns were observed for tobacco (RRR = 2.16, 95% CI: 1.17–4.00, p = 0.014; 15.3% vs. 8.8%) and cannabis (RRR = 3.49, 95% CI: 2.00–6.10, p < 0.001; 23.8% vs. 10.4%). The largest disparity was observed for illicit substances, where survivors had more than 14 times the relative risk of reporting increased use (RRR = 14.31, 95% CI: 3.54–57.89, p < 0.001; 6.8% vs. 0.6%). Notably, survivors were also significantly more likely to report *decreased* substance use across several categories, with the most striking effect for cannabis (RRR = 8.16, 95% CI: 2.81–23.70, p < 0.001; 9.1% vs. 1.7%). However, in absolute terms, decreases represented a smaller proportion than increases across all substances and both groups. This pattern likely reflects the heterogeneity of survivors’ circumstances during lockdowns — including shelter-based restrictions on substance access, disrupted supply chains, or survivor-driven harm reduction efforts. Non-IPV respondents showed comparatively greater stability overall, though a notable minority still reported increases — particularly for alcohol (21.9%) and cannabis (10.4%). For IPV survivors, the qualitative data contextualize the mechanisms underlying these quantitative disparities, revealing substance use as intertwined with coping and survival. As one IPV survivor describes, substance use was a coping mechanism and got worse because of the IPV experience:

“*I went down a bad path when this stuff [IPV] happened*,* and I started using drugs. I was on Methadone*,* you know. I’m off of Methadone now…And I know that it was because of what happened … Right? [A] coping mechanism. So*,* I turned to those things [substances] for coping mechanism and my life [got] worse.*” *(Survivor_4)*.

Service providers emphasized the severity of substance use escalation among IPV survivors in shelters during the COVID-19 lockdowns. One provider described witnessing alarming increases in substance use, even among individuals with no prior history of use:

“*Even folks who are not using before…they started*,* yeah*,* yeah*,* heightened use of alcohol. For the clients who use other harder drugs… yes*,* I saw that there were deaths of clients that I know. Uh*,* which is really sad…of opioid overdose.*” *(Service Provider_6)*.

Another provider reflected on how pandemic-related isolation and safety protocols delayed staff awareness of residents’ substance use:

“*Because they were in their room*,* we were often not aware [of substance use]. It took us longer to realize the kind of clients we’re serving in the circumstances that they hide. A lot of clients were telling us - they never used to drink before*,* but the lockdown measures - they turned to alcohol*,* or*,* you know*,* excessive use of Tylenol or whatever muscle relaxants…. Those were not realized [by staff] for a period of time.” (Service Provider_3)*.

By contrast, most non-IPV respondents expressed stability in their substance use habits during the pandemic (Table 3). One non-IPV participant wrote:

*“I don’t drink or smoke or use cannabis or drugs. Nothing has changed.” (non-IPV participant_519)*.

This sentiment was echoed by several others (*n* = 6), reflecting a broader pattern of behavioural consistency in the non-IPV group.

Across both groups, most participants reported increases in screen use during the lockdown, with no significant differences in the proportion of yes IPV and no IPV who reported increased TV watching (70.8% vs. 72.9%) or internet use (76.9% vs. 77.2%). No significant differences were found for decreases in TV watching. Differences were observed in patterns of reduced internet use, where IPV survivors were more likely to report decreases compared to no IPV (7.5% vs. 2.9%; RRR = 3.56, 95% CI 1.28–9.89, p = 0.015). For some IPV survivors, this reduction may have been linked to technological control and abuse by partners. One survivor shared:

“*I wish there had been something [to access in terms of services]*,* but he had broken my cell phones*,* alienated me*,* and kept me isolated. It was hard for me to access help because of the control he had over me. If he found out I was talking to someone*,* he’d break my phone.” (Survivor_2)*.

For physical activity, IPV survivors were more likely to report decreased exercise compared to no IPV (46.4% vs. 36.7%, RRR = 1.73, 95% CI: 1.05–2.87, p = 0.032) and were less likely to report no change (27.3% vs. 36.9%, p = 0.045). No differences were observed for increased exercise. Respondents frequently cited loss of access to fitness spaces and diminished motivation. A non-IPV survivor noted:

*“Being isolated for months became depressing. Especially since one of my outlets is going to the gym*,* it became hard to get into [exercise]at home.” (non-IPV participant_27)*.

Service providers also noted reduced activity levels among IPV-yes shelter residents, linking it to emotional distress and lack of access to facilities:

*“Clients reported to me … they’ve gained so much weight… a lot of gyms closed.” (Service Provider_6)*.

Changes in eating habits were frequently reported, although adjusted analyses showed no significant differences between yes IPV and no IPV. A similar proportion of both groups reported increased junk food consumption, and decreases were also comparable. Despite the lack of significant quantitative differences, qualitative accounts highlighted disruptions in eating patterns during the lockdown. Survivors described disordered eating behaviours, with some turning to food for comfort and weight gain, while others experienced appetite loss and weight decline:

*“Oh*,* my gosh! During Covid*,* because we were isolated*,* all I did was eat … So*,* it was very bad. Yeah*,* I was- oh*,* I was almost 200 something pounds during COVID.” (Survivor_8)*.

*“Yeah*,* like I wasn’t myself at all. Like it was crazy*,* and it was like noticeable. And I like*,* I lost so much weight like I was just like I like literally was like a shell of myself.” (Survivor_12)*.

Service providers echoed these experiences, noting widespread weight gain and food-related coping among shelter clients:

*“So yeah … clients reported to me that they are eating more….” (Service Provider_6)*.

Sleep disturbances were prominent among IPV survivors. They had significantly higher odds of reporting decreased sleep quality compared to the no IPV group (61.4% vs. 40.3%, RRR = 3.24, 95% CI: 1.87–5.62, p = 0.001) and were less likely to report no change (21.7% vs. 43.2%, p < 0.001). Increased sleep was also more common among survivors (16.9% vs. 16.5%, RRR = 2.19, 95% CI: 1.12–4.29, p = 0.022). Interviews with survivors and service providers highlighted a range of sleep disruptions - including excessive sleep and insomnia- often linked to emotional distress, trauma and disrupted routines. Participants described how the psychological toll of isolation and abuse intensified sleep disruptions:

*“Definitely. So*,* my behavior change[d]. I didn’t feel like the same person I was. I was isolating myself. I wasn’t*,* you know. I wasn’t the happy person that I was before and when I got out of the relationship*,* it took me literally like a long time*,* couple of like months*,* like where I would just sleep for hours on end*,* just to like*,* you know … it was like my body decompressing itself right*,* my mind decompressing itself of the whole situation. You know I would sleep hours on end.” (Survivor_4)*.

*“I was like smoking cigarettes and vaping and stuff like that*,* and like just not sleeping. Um not eating and stuff like that. I really just didn’t really put effort into myself and yeah*,* I really just didn’t care.” (Survivor_3)*.

Changes in help-seeking behavior during COVID-19 lockdowns differed significantly between IPV survivors and non-IPV individuals, with notable contrasts in both informal (e.g., friends and family) and formal (e.g., healthcare providers, counselling services) supports. IPV survivors were significantly more likely to report decreases in informal support compared to non-IPV individuals (29.5% vs. 17.3%), with over twice the relative risk of a decline (RRR = 2.53, 95% CI 1.47–4.36, *p* = 0.001). At the same time, they were also more likely to report increases in informal support (27.2% vs. 22.6%), though this difference was modest (RRR = 1.76, 95% CI 1.01–3.07, *p* = 0.045). Patterns in formal help-seeking were even more pronounced. IPV survivors were nearly five times more likely to report increased use of formal supports compared to non-IPV individuals (34.7% vs. 12.9%; RRR = 4.75, 95% CI 2.74–8.24, *p* = 0.001), making this the strongest observed association. They were also more likely to report decreases in formal support (22.3% vs. 13.7%; RRR = 2.98, 95% CI 1.68–5.29, *p* = 0.001). By contrast, the majority of non-IPV individuals reported no change in their help-seeking behaviors, both informally (60.1% vs. 43.3%) and formally (73.4% vs. 43.0%). These results suggest more polarized engagement patterns among IPV survivors compared to the relative stability seen among non-IPV individuals.

Several non-IPV respondents described how their social support systems remained largely intact during lockdowns, with only minor adaptations to format or logistics:

*“Community with friends and family decreased only in terms of face-to-face interactions.” (non-IPV participant_1031)*.

Another described how technology assisted adaptations, like virtual visits and delivery services, helped maintain a sense of normalcy:

*“We did not really change much except for staying home and shopping with Instacart*,* and we visited at a distance from our older daughter early on … KN95 masks were and are a blessing.” (non-IPV participant_188)*.

In contrast, many IPV survivors described significant barriers to seeking support. These included being psychologically overwhelmed, lack of awareness, and the need for proactive outreach for services. One survivor noted:

*“When we have so much in our heads… we don’t pay attention to things. I remember that the whole year*,* going through what I was going through*,* I didn’t focus much on what was out there [in terms of seeking help]. Unless somebody approached me and explained it to me*,* I didn’t have the capability to find that on my own… I didn’t know.” (Survivor_Int_8)*.

Others pointed to fear of judgment and shame as significant deterrents to help-seeking, even when the need was clear:

*“I don’t know if there are [formal] services I could turn to*,* but for me*,* again—it’s telling people my story. And I don’t know… It’s people’s judgment. I’ve always been the type of person who cares too much about what people think of me. And it’s also like… you’re embarrassed*,* right? You don’t want to tell anybody about what’s happening in your life.” (Survivor_Int_4)*.

Some survivors also described how internalized stigma and deeply ingrained beliefs about self-reliance made it difficult to acknowledge the need for support:

*“I think that stigma becomes internalized and like*,* I think that’s part of like*,* why*,* like*,* I wouldn’t have reached out for the supports*,* even though I absolutely should have. Is because I would think the same things*,* and it sounds horrible to like say out loud*,* but like I’m a smart*,* like*,* confident*,* assertive person*,* like this doesn’t happen to me.” (Survivor_Int_12)*.

#### Psychological and physiological

Table 4 presents results from the multivariate logistic regression models predicting poor mental (psychological) and poor physical (physiological) health outcomes (see Additional File 5 – Table S3 for full model details). Women who had experienced IPV were 2.5 times more likely to report poor mental health (aOR = 2.460, 95% CI: 1.273–4.753, *p* = 0.007; Table 4) and nearly twice as likely to report poor physical health (aOR = 1.8435, 95% CI: 1.003–3.359, *p* = 0.049; Table 4) compared to those with no history of IPV. The models also revealed strong interdependence between mental and physical health outcomes. Women reporting poor physical health were nearly seven times more likely to also report poor mental health (aOR = 6.997, 95% CI: 4.245–11.530, *p* < 0.001; Table 4), and vice versa (aOR = 7.056, 95% CI: 4.272–11.660, *p* < 0.001; Table 4), highlighting the bidirectional nature of these health pathways.

Qualitative data further illustrates how IPV, compounded by pandemic-related stressors, intensified distress among survivors. One woman described how her symptoms worsened during the lockdown:

*“Yes [it got worse during COVID-19] … and it was like*,* anxiety*,* depression*,* and all that was too much. I was on medication [for depression].” (Survivor_11)*.

Service providers also noted heightened anxiety among women residing in shelters:

*“We definitely did see an increase in general anxiety among the women who lived here.” (Service Provider_3)*.

Survivors emphasized how multiple overlapping stressors – such as psychological distress, financial insecurity, caregiving responsibilities, and isolation - further deteriorated their mental and physical health. One survivor recounted how her partner’s pre-existing mental health challenges escalated during the pandemic, leading to increased violence:

*“Before COVID*,* I was married to the father of my kids. There was love in the beginning. But as life stresses piled up*,* I was able to work through the challenges*,* but he wouldn’t. He went through a major mental shift*,* and things got really difficult—really difficult. ” (Survivor_8)*.

She goes on to describe how her partner became emotionally, financially, and physically violent during the COVID-19 pandemic. Job loss increased household tension and stress, leading to further violence.

*“I feel like it just got worse once COVID hit because of financial stress. Not being able to work… that really contributed to a lot of it because it was just so much stress. It was all about blame. I was working full-time*,* and then when COVID hit*,* I lost my job.” (Survivor_4)*.

Caregiving responsibilities became increasingly overwhelming for many IPV survivors during the lockdowns, particularly with school closures and the sudden shift to online learning. One survivor shared the difficulty of managing her child’s schooling at home:

*“The kids had to go to school online*,* and it was terrible. My son couldn’t sit still. It was a war because I wanted him to go to school*,* but I couldn’t be fighting with my son every day.” (Survivor_8)*.

Service providers echoed these challenges, noting widespread increases in caregiver strain:

*“The school closing*,* the online learning… that added to women’s stress.” (Service Provider_10)*.

For some IPV survivors, psychological abuse intensified during the lockdowns, especially as pandemic-related restrictions removed access to their usual coping mechanisms. One survivor described how her partner’s persistent body-shaming contributed to disordered eating, which worsened when she could no longer attend the gym:

*“He would make comments about my body*,* saying it could be toner. I was heavily going to the gym*,* trying to lose weight and be smaller and smaller. When COVID hit and I couldn’t go to the gym anymore*,* I thought*,* ‘Oh my gosh*,* he’s going to be on me about my body*,* and I’m going to gain weight.’ So*,* then gyms closed*,* and I freaked out. I felt huge. Eventually*,* I was so stressed that I turned to food for comfort and developed binge eating disorder. It was this whole spectrum—restriction*,* then overconsumption.” (Survivor_14)*.

Even within shelters, where women sought refuge, pandemic restrictions introduced new challenges. Service providers reflected on how mandatory isolation and testing protocols increased survivors’ sense of confinement, especially among those already experiencing mental health crises.

*“Some days were not so good. You could see them being isolated. They had to wait four days for COVID test results… they couldn’t leave their rooms except to go to the washroom. Some were already in a mental health crisis when they arrived*,* and now they were locked in*,* with their children. We were controlling them…It was a difficult time. Really difficult.” (Service Provider_6)*.

Several survivors described a marked escalation in the severity of physical abuse during the pandemic. One woman reflected on how public health measures—such as mask mandates—unintentionally enabled abusers to conceal visible injuries:

*“… Got physical and stuff… like punching my face like*,* and just like aggressive*,* really breaking beer bottles over my head*,* and stuff like that. And then*,* after that*,* like*,* I think it just got*,* I don’t know like he just seemed to be having more power and control over me. And so*,* then it went from that to like strangling and throwing me into walls and all that stuff… And like even sexual abuse… And I think because of COVID too*,* and like wearing masks like … ‘Oh*,* alright.’ Like ‘No problem*,* no one’s gonna notice.’ Cause I literally could just wear a mask over my face so like my mouth is swollen [no one would see] …?” (Survivor_3)*.

Beyond physical violence, survivors also described physiological symptoms likely linked to chronic stress and compromised immunity. A service provider recounted persistent infections among shelter residents:

*“Yeah*,* I just recall hearing… [the women] having things like infections that just won’t go away. Those sorts of things*,* right? So*,* Like things immune response and struggling with getting their healthcare needs met*,* because*,* yeah*,* COVID.” (Service Provider_4)*.

One survivor reflected on her own experience with illness while in an abusive relationship:

*“I think I did get sick more often when I was in that [abusive] relationship. Like*,* if I think about it like even just thinking about like common colds and stuff. Like I don’t get sick very often…like I don’t think I’ve been sick otherwise*,* but when I was in that relationship*,* I did get sick quite often.” (Survivor_12)*.

These accounts underscore how the chronic stress of IPV, exacerbated by the pandemic, may have further compromised survivors’ physical health, increasing susceptibility to illness and stress-related health conditions.

The regression model also revealed that women with male partners had more than twice the odds of reporting poor mental health (aOR = 2.283, 95% CI: 1.150–4.532, *p* = 0.018; Table 4). While this finding was not explicitly confirmed or contradicted in the qualitative interviews, the data primarily reflects the experiences of heterosexual relationships; 13 of the 14 survivors interviewed had male partners. Additionally, all service providers worked in women-only shelters, which may have influenced the scope of experiences captured in the qualitative data. As a result, the qualitative findings neither strongly support nor challenge this particular regression result.

Access to information and services emerged as another key determinant of well-being. The model showed that women who lacked awareness of available services during the pandemic had significantly higher odds of reporting poor physical health (aOR = 1.733, 95% CI: 1.107–2.712, p = 0.016; Table 4). In interviews, survivors frequently emphasized how not recognizing IPV – or not knowing where to seek support - delayed help-seeking and impacted health outcomes.

*“It had like never occurred to me that*,* like that [IPV] was actually like what I was experiencing. Which sounds so strange*,* because at this point it’s like 4 years of it*,* like going on 5. So*,* like*,* I don’t know why*,* like it had never occurred to me … I was Googling it*,* and it was like the list of all the signs and all the different categories. It was kind of like as if he was using it as a [check]list. You know what I mean*,* “Oh*,* my God! Like*,* does he have this like printed out and laminated somewhere?” Like it was crazy when I was reading it… not to like to make a joke about it…I was like*,* that is like wild. So … in terms of like that kind of support*,* like just informational.” (Survivor_12)*.

Some described searching for help online, only to find unclear or inaccessible information:

*“I never knew there was help. I googled like Crisis Line*,* but there was no information [on] What it was. What it is. Like what is that for what? … What kind of support? It’s very hidden. Certain topics and programs you don’t really know unless you are in a really*,* really bad place.” (Survivor_8)*.

The model also identified a strong association between decreased informal help-seeking (e.g., family and friends) and poor mental health (aOR = 3.222, 95% CI: 1.701–6.106, *p* < 0.001; Table 4). Survivors described how shame, fear of judgment, and internalized stigma contributed to social withdrawal, intensifying their sense of isolation and emotional distress. One survivor explained how embarrassment and perceived judgment led her to distance herself from others:

*“We pushed a lot of people away because people would see that*,* and then I would cry about it*,* but I would do nothing about it. And then you feel worse with yourself. Because here I am*,* sitting with some of the people … who can clearly see what I’m going through*,* and I bet they were wondering*,* ‘What are you doing with this person?’ So*,* I got to a point where I was very afraid to talk to people*,* embarrassed to talk to people*,* and feeling like there was no point because I felt judged.”* (*Survivor_8)*.

Another described how her identity as a strong and independent person made it difficult to ask for help – even from close family:

*“It’s like I’m always looked upon as the one that’s helping people*,* always there for people. And then*,* now*,* I’m the one that needs help. And I’ve always been that type of person where I do everything myself*,* and I don’t ask people for help. Even my mom still doesn’t even know what happened. My family still doesn’t even know what happened… And it’s also like*,* you’re embarrassed*,* right? So*,* you don’t want to tell anybody about what’s happening in your life.”* (*Survivor_4*).

In some cases, survivors became so isolated that seeking help no longer seemed possible. This isolation stemmed from two factors: erosion of self-efficacy/autonomy through the perpetrator’s control, and withdrawal of support networks frustrated by repeated attempts to leave and return. When survivors’ belief in their ability to escape is systematically broken down, they may see themselves as powerless or undeserving of help—particularly when others assume they are “choosing” to stay rather than recognizing complex barriers to leaving. This dynamic is captured in one survivor’s account:

*“A thousand percent my family knew…like every single person knew about it. They just- because I kept going back. It’s like*,* they say [I] just wanted it*,* like I chose it*,* and I’m just like*,* “Oh*,* I chose to get strangled and pushed on the highway. I chose to get raped and pinned down”. I don’t choose any of that*,* but they just think*,* “Oh*,* you keep going like you’re choosing it”. Like when you’re like in a relationship like that for so long and then you just like really don’t have anybody cause they give up on you. And you just have that one person you just like I don’t know like he made me think like he owned me*,* and stuff like that he diminished me where*,* like he had me like literally in the wintertime in broad underwear*,* laying that dog bed on the balcony*,* eating dog food. At that point you feel like you’re just- you have no value. You’re worthless. So*,* like why*,* even try*,* you know what I mean? Why push to get out of that when like you just really feel like you’re nothing…” (Survivor_3)*.

Others shared how internalized stigma and societal expectations discouraged help-seeking, even when they recognized the severity of their situation:

*“I obviously knew the gravity of the situation at the time*,* but to be able to see it and accept it for what it was*,* in order to be willing to reach out for help*,* you know? But because there was so much shame and a lack of support*,* I was just trying to do the same thing that everyone else around me was doing—just sweep it under the rug. Not really accepting that this is my actual situation*,* and I need help. I think if I was better able to accept it*,* I would have reached out for help. I think more support from friends and family*,* but also more public awareness… like*,* I kind of get why the stigma exists.” (Survivor_12)*.

For many, the absence of support from close social circles not only deepened their isolation but also made it more difficult to seek help beyond their immediate networks:

*“I think that was very isolating. And I think because the people directly around me didn’t offer me a lot of support*,* that kind of stopped me from reaching out to other people like my family or friends from home. At the time*,* I felt embarrassed*,* right? I think that kind of got me stuck in the cycle more—just feeling isolated. And then*,* like*,* even if someone knows what’s going on*,* it’s not gonna matter. They’re not gonna help.” (Survivor_12)*.

Cultural and financial dynamics further complicated informal support-seeking in some cases, particularly when abusers used financial leverage to manipulate family members. One survivor shared:

*“He used my family… My family said*,* ‘It’s okay*,* let him. He’s old*,* he’s not able to see the kids’ … He kept going to my mom. And sometimes*,* to be honest*,* he would give my mom money…you know*,* because it’s a crisis [back home]. Like*,* if you give her one hundred [dollars]*...*” (Survivor_5)*.

These narratives illustrate how survivors’ ability to seek out informal support was shaped not just by internal barriers like shame and self-perception, but also by external factors such as cultural norms, financial dependence, and systemic misunderstanding of IPV. The regression findings reinforce this complexity, highlighting the significant link between reduced informal support and poor mental health outcomes.

**Table 4:** Key Findings from Logistic Regression Models Predicting Poor Mental and Physical Health Among IPV and Non-IPV Survey Respondents During COVID-19 Lockdowns in Ontario (*n* = 559)

Variable	Outcome	aOR	95% CI	*p*-value	
Poor Mental Health					
IPV during COVID-19	Poor mental health	2.46	1.273–4.753	0.007	
Partner gender (man)	Poor mental health	2.283	1.150–4.532	0.018	
Informal help-seeking decreased	Poor mental health	3.222	1.701–6.106	0.000	
Poor physical health (for mental health outcome)	Poor mental health	6.997	4.245–11.53	0.000	
Poor Physical Health					
IPV during COVID-19	Poor physical health	1.835	1.003–3.359	0.049	
Enough information about services (no)		Poor physical health	1.733	1.107–2.712	0.016
Poor mental health (for physical health outcome)		Poor physical health	7.056	4.272–11.66	0.000

*aOR* = adjusted odds ratio; *CI* = confidence interval

#### Propensity score matching secondary analysis

To assess whether the observed associations between IPV exposure and health outcomes could be attributed to pre-existing demographic differences between groups, a propensity score matching [54, 55] secondary analysis was conducted. IPV-exposed women were matched to non-IPV women with similar sociodemographic profiles across 13 characteristics. Of the 150 IPV-exposed women in the analytic sample, 18 were excluded from the matching procedure due to missing values on one or more matching covariates, leaving 132 eligible for matching. Of these, 76 were successfully matched to a non-IPV comparator; the remaining 56 could not be matched because no comparable non-IPV counterpart existed in the sample. Importantly, these unmatched women were significantly more likely to be young (aged 18–34: 70% vs. 40%, *p* = 0.001), to have low household income (59% vs. 30%, *p* = 0.001), to identify as racialized or Indigenous (55% vs. 37%, *p* = 0.031), and to have a partner with moderate or high substance use (98% vs. 40%, *p* < 0.001) — suggesting they represented the most structurally marginalized women in the sample. Their exclusion from the matched analysis is itself a meaningful finding, reflecting a fundamental limitation of propensity score matching in health equity research: the women who are hardest to match are often those whose experiences are most extreme. In the matched sample, the association between IPV exposure and poor mental health was confirmed and strengthened (aOR = 3.92, 95% CI: 1.24–12.37, *p* = 0.020). The association with poor physical health was attenuated and no longer statistically significant (aOR = 1.69, *p* = 0.284), most likely reflecting reduced statistical power in the smaller matched sample rather than the absence of a true effect. Key behavioural findings were replicated, including significantly higher rates of increased formal support-seeking (RRR = 6.18, *p* < 0.001), decreased sleep (RRR = 2.74, *p* = 0.018), increased cannabis use (RRR = 2.65, *p* = 0.035), and decreased exercise among IPV-exposed women (*p* = 0.034). Full results are reported in Additional File 6.

## Discussion

This mixed methods study examined whether women who experienced IPV during the COVID-19 lockdowns faced greater health inequities compared to those who did not. Guided by Berkman and Krishna’s (2014) conceptualization of behavioural (e.g., substance use, diet, exercise, help-seeking), psychological (perceived mental health), and physiological (perceived physical health) pathways, findings revealed that IPV survivors reported poorer outcomes across all pathways, indicating a heightened risk of health inequities during stressful life events such as the COVID-19 pandemic.

### Participant demographics and representativeness

The demographic profile of IPV survivors included in this study aligns closely with patterns identified in other Canadian research. In the survey data, survivors were more likely to be younger, to report having younger partners [2, 18–34, 56], to identify as racialized or Indigenous [2, 6, 7, 56], to report lower household income [57], and to take on informal caregiving roles or parenting responsibilities [58, 59], compared to women with no reported IPV. The qualitative sample, although smaller and with slightly higher average age (37 years), generally mirrored the same demographic characteristics. Half of the interviewed survivors identified as racialized or Indigenous, over 70% reported caregiving roles, and all described financial strain during the pandemic. Four participants also referenced having partners who were immigrants or non-permanent residents, consistent with survey trends.

These points of demographic convergence increase the interpretive power of the mixed methods design, reinforcing the relevance of survivor voices in contextualizing and expanding on survey findings. Still, the slightly older profile of interviewees may reflect lower willingness or ability among younger survivors to participate in in-depth interviews. Research suggests younger IPV survivors often face barriers to participating in qualitative studies – such as lack of privacy, fear of being identified, concerns around stigma, or discomfort disclosing IPV [60, 61]. Additionally, recruitment through a provincial shelters and transition housing organization may have skewed the sample toward women who were already engaged with services – typically a demographic further along in the process of leaving abusive relationships.

Regional variation in IPV reporting also merit consideration. A significantly higher proportion of IPV survivors resided in Eastern Ontario. This may be partly due to structural and historical factors. For example, Renfrew County - the site of a high-profile triple femicide in 2015 – was the focus of a 2022 coroner’s inquest that issued 86 systemic recommendations, including a call to declare IPV an epidemic. In 2023, Renfrew became the first jurisdiction in Canada to formally adopt this declaration, prompting a wave of similar actions across the province [62]. In contrast, the lower proportion of IPV survivors in Western Ontario may reflect relatively better access to services in mid-sized urban centres, where infrastructure is denser and rural barriers less pronounced [63, 64].

While LGBTQ2 + participants accounted for a sizable proportion of the IPV survivor in the survey (19.5%; Table 1), only one interview participant self-identified as being in a same-sex relationship. As a result, the qualitative data do not fully capture the unique experiences of LGBTQ2 + survivors, who often face distinct stigma, discrimination, and service barriers [65]. This may reflect recruitment limitations or structural barriers to participation from queer and trans communities [65]. Future research should prioritize inclusive recruitment strategies to ensure broader representation of LGBTQ2 + voices in qualitative analyses of IPV and health.

### Health pathways

In line with the study hypothesis, the women survivors of IPV reported greater negative outcomes compared to no IPV across all three health pathways—behavioural, psychological, and physiological—during the COVID-19 lockdowns. While existing literature has documented individual outcomes such as increased substance use or declining mental and physical health, this study is among the first to take an approach grounded in Berkman and Krishna’s (2014) conceptual model of how macro-level structural conditions - such as government-mandated pandemic lockdowns - affect social networks (i.e., social and health supports) and ultimately impact health through behavioural, psychological and physiological pathways of survivors of IPV [15]. The mixed-methods design allows for a layered understanding of these outcomes, with the survey offering provincial-level insight and the interviews providing rich, contextualized narratives of lived experience. The following subsections discuss each of these pathways in more detail.

### Behavioural pathway

Before the pandemic, IPV was already associated with increased health-harming behaviours such as substance use and smoking [66, 67]. With the onset of COVID-19, some studies similarly identified a rise in such coping mechanisms among both survivors and perpetrators of IPV [68, 69]. What this study adds—particularly through qualitative insights from service providers—is that even in contexts intended for safety and support (such as shelters), the lockdowns undermined visibility and connection. The extreme isolation made it difficult for shelter staff to detect escalating substance use or psychological distress occurring behind closed doors, with some individuals who had not previously used substances beginning to do so as a coping strategy. This highlights a gap in crisis care—shelters must be able to maintain connection and oversight during public health emergencies for high-risk groups to respond accordingly.

Although TV and internet use increased broadly during COVID-19 lockdowns due to restricted outdoor activity and more time spent at home, this study revealed some important distinctions. The majority of both IPV survivors and women who did not experience IPV reported increased screen time. This is consistent with other pandemic studies documenting increased media consumption as a coping or boredom-management strategy during periods of confinement and stress [70]. However, a significantly higher proportion of IPV survivors also reported decreased use of internet (7.5% vs. 2.9%; Table 3) compared to non-IPV women. This reduction in internet access among survivors may reflect underlying issues of technological violence and coercive control [71, 72]. Coercive control refers to a pattern of ongoing behaviours—both physical and non-physical—used to dominate, isolate, and regulate a partner’s everyday life [73, 74]. Rather than discrete incidents, it is continuous and cumulative, operating through tactics such as surveillance, deprivation, and restriction of autonomy to produce what Stark describes as “entrapment,” effectively limiting a survivor’s freedom and capacity for independent decision-making. In the context of COVID-19 lockdowns, these dynamics were often intensified, with survivors describing restricted access to technology, communication, and support networks, highlighting how coercive control can extend into digital spaces and further constrain help-seeking. Abusers deliberately restrict, hijack, control or damage access to technology (e.g., phones, Wi-Fi, or digital platforms) as a means of isolation [72]. These findings align with what IPV survivors discussed in their interviews and emerging literature on digital and technological abuse as an extension of IPV (i.e., technology facilitated coercive control), which gained new relevance during COVID-19 when virtual access to services, support networks, and safety planning became essential [71, 72, 75, 76]. The observed internet decrease among survivors, despite pandemic-wide increases in internet use, suggests that public health interventions should account for digital autonomy and safety for survivors of IPV. Future research should continue to explore how technological abuse restricts health information access, help-seeking, and psychosocial wellbeing during emergencies.

Evidence from British Columbia suggests that COVID-19 negatively impacted women’s physical activity behaviour and mental well-being [77]. IPV survivors in this study were almost twice as likely to describe a decrease in physical activity during lockdowns compared to non-IPV women, despite shared barriers like gym closures and mobility restrictions. Survivors faced additional unique challenges such as isolation with the abuser, stress, PTSD, financial strains, reduced safety and privacy [9]. This made it more difficult to maintain exercise routines. While the adjusted models in this study did not show significant differences between yes IPV and no IPV in junk food consumption, qualitative accounts revealed that survivors frequently experienced food-related coping challenges. Narratives described both increased reliance on food for comfort and reduced appetite leading to weight loss, with service providers observing similar patterns among their clients. These patterns align with literature that links IPV with disordered eating as coping responses during compounded crises (IPV and pandemic) [78, 79].

In response to feedback from the project’s advisory committee and pilot phase data collection, sleep quality was integrated as a key health indicator in both the survey and interview instruments. IPV survivors reported a significantly greater decline in sleep quality during the pandemic compared to non-IPV women. For some, sleep served as a temporary refuge, for others, fear and anxiety severely disrupted rest. Service providers confirmed that sleep disturbances were already prevalent among IPV survivors but noted that pandemic-related lockdowns exacerbated these issues - particularly for individuals facing safety threats, mental health challenges, or overcrowded in shelters. These findings are consistent with international literature. For instance, a study from the United Kingdom reported widespread sleep disturbances during the COVID-19 pandemic [80], while a recent narrative review by Carrentante et al. (2025) identified sleep disruption as a frequent symptom of IPV. However, few studies have explicitly examined sleep quality at the intersection of IPV, pandemic-related restrictions, and survivor health [81]. These results suggest that sleep should be considered a critical health indicator in IPV support settings and warrant greater attention in future research.

The divergence in help-seeking behaviours between IPV survivors and non-IPV women during the COVID-19 lockdowns reveals significant barriers to accessing support. While some IPV survivors increased their use of formal services, a notable proportion reported decreased engagement with both formal and informal supports. In contrast, most no IPV participants reported no change, suggesting more stable access to social networks and healthcare access. This disparity highlights the compounded vulnerability of survivors navigating trauma amid a public health crisis, as documented in prior work examining barriers to formal and informal support during Ontario’s COVID-19 lockdowns [35]. As reflected in the interview data and consistent with prior research, psychological distress, shame, and internalized stigma often hindered help-seeking, even when services were technically available. Survivors expressed fears of judgment, dismissal, or not being believed – factors well documented in the literature as barriers among IPV-affected populations [82, 83]. These findings underscore the need for trauma- and stigma-informed outreach strategies that proactively engage IPV survivors, especially during emergencies, when traditional support systems may be disrupted.

### Psychological and physiological pathways

Survivors were more than twice as likely (aOR = 2.46; Table 4) to report poor mental health and nearly twice as likely (aOR = 1.84; Table 4) to report poor physical health compared to non-IPV women during COVID-19 pandemic. While both Canadian and international studies have explored the health impacts of IPV under routine circumstances, these typically focus on mental health alone and seldom include direct comparisons between yes IPV and no IPV groups [84–88]. A recent US-based quantitative cohort study by Scoglio et al. (2023) examined associations between IPV and adverse mental health and behavioural outcomes during the first 1.5 years of the pandemic, finding that IPV was associated with higher odds of depression, anxiety, PTSD symptoms, poorer sleep, and increased substance use [89]. These findings are consistent with and further support the results of the present study, while the mixed-methods design and Canadian context extend this evidence base by integrating survivor and service provider perspectives within a theoretically grounded health pathways framework.

### Psychological

Consistent with national trends during COVID lockdowns, participants described how pandemic-related disruptions - such as job loss, school closures, increased caregiving demands, and prolonged isolation - intensified pre-existing mental health struggles [9, 11, 12]. Similar to another Canadian study, service provider interviews reinforced these accounts, observing that pandemic protocols in shelters lead to isolation and heightened anxiety and loneliness for women already living in crisis [90]. In our study, as in other published research, survivors who remained confined with abusive partners during lockdowns reported that the perpetrators’ own psychological distress - exacerbated by the loss of external outlets, financial stress, job loss - appeared to escalate the intensity, frequency, and severity of abuse, with substantial consequences for survivors’ mental and physical well-being [91]. Additionally, diminished access to informal support significantly predicted poor mental health outcomes. Interviews revealed that internalized shame and stigma commonly discouraged help-seeking, while others described feeling dismissed or unsupported by family and community networks. Survivors frequently used terms like ‘invisible’ or ‘worthless’ to describe their experiences – underscoring how IPV can erode self-worth and self-efficacy. These findings align with Overstreet and Quinn’s (2013) IPV stigmatization model, which identifies three key stigma components – cultural stigma, internalized stigma, and anticipated stigma – that hinder help-seeking among IPV survivors [92].

### Physiological

Both survivors and service providers in this study described an escalation in the severity of physical abuse and injuries during the COVID-19 pandemic. Some participants also reported symptoms suggestive of decreased immune function, which they attributed to the ongoing stress of living in an abusive environments. These experiences are consistent with the concept of *allostatic load*, described by Berkman and Krishna (2014) as a key physiological pathway through which chronic stress and adverse social conditions—such as ongoing IPV, prolonged fear and lack of social support—can erode health [15]. Allostatic load refers to the cumulative “wear and tear” on the body’s stress-regulation systems from repeated or prolonged stress exposure [93], affecting multiple physiological domains, including the neuroendocrine, cardiovascular, metabolic, and immune systems. Survivors of IPV are frequently exposed to unpredictable and persistent stress, which repeatedly activates the hypothalamic-pituitary-adrenal (HPA) axis and the sympathetic-adrenal-medullary system, often without adequate buffering supports [81]. Over time, this dysregulation may lead to heightened inflammation, impaired immune function, and increased risk for infections, chronic pain, cardiovascular disease, and psychiatric disorders [94, 95].

Additionally, women who lacked access to information about available services during the pandemic had significantly higher odds of reporting poor physical health. Survivors described uncertainty in recognizing their experiences as abuse and delays in seeking support, highlighting how gaps in information can exacerbate health risks. These findings are consistent with broader disaster literature emphasizing the importance of timely and accessible information during public health emergencies [96].

Importantly, the study also identified a bidirectional relationship between mental and physical health – each significantly increasing the likelihood of the other - reinforcing the need for integrated and holistic approaches to health support. Many studies have described these interconnections in IPV-affected populations [81, 86, 94, 97]. This relationship reflects well-established links between psychological distress and physiological functioning. Chronic stress and trauma associated with IPV can activate prolonged stress responses, contributing to sleep disruption, immune dysregulation, and other physical symptoms. In turn, declining physical health may exacerbate psychological distress by reducing coping capacity, increasing fatigue, and limiting individuals’ ability to engage with supportive resources. During crisis conditions such as pandemic lockdowns, these reinforcing pathways may intensify, highlighting the importance of coordinated mental and physical health services for IPV survivors.

### Reflexive methodological insight

This study used a quota sample due to recruitment constraints at the time and was not intended to be representative of a broader population; therefore, findings should be interpreted within the study cohort. To address concerns regarding baseline differences between groups, we conducted a propensity score matched analysis to improve comparability. Findings from the matched analysis were largely consistent with the main results, suggesting that the observed attenuated differences are unlikely to be solely driven by baseline sample characteristics. However, matching reduced the analytic sample by excluding individuals who could not be matched [98–100]. Those excluded were younger, lower-income, racialized, and exposed to higher partner substance use, suggesting that the method may omit individuals experiencing greater structural vulnerability. This highlights an important equity-related limitation and reinforces the value of the qualitative component of this study that is capturing perspectives that may be underrepresented in the additional matched analysis.

### Implications for practice and policy

The findings of this study, interpreted alongside the perspectives of community partners with sustained experience supporting IPV survivors through COVID-19, yield several implications for service providers, public health and government systems, policymakers, and emergency planners.

Emergency preparedness frameworks must explicitly integrate gender-based violence and IPV as a priority concern [9, 101, 102]. The findings of this study illustrate how lockdown-related conditions — confinement with abusive partners, disrupted service access, severed informal support networks, loss of employment, and reduced shelter capacity — directly worsened health outcomes across behavioural, psychological, and physiological pathways for IPV survivors. As climate-related disasters become more frequent, these conditions risk being replicated: fires, floods, and forced relocation restrict movement and sever community ties in ways that mirror pandemic confinement, and emergency planners must anticipate how these dynamics intersect with coercive control and elevate the risk of IPV [9, 35].

This study also provides VAW service providers and organizations with documented evidence to advocate for emergency planning policies that explicitly account for how public health directives can be weaponized within abusive relationships. The qualitative data showed that movement restrictions and isolation requirements were used by some abusive partners to deepen surveillance and control — in a pattern consistent with documented cases of perpetrators framing government mandates as justification for confinement [103]. Many survivors experienced sustained, uninterrupted coercive control and violence with no respite during lockdown, contributing to the declines in mental health, sleep quality, and help-seeking documented in this study. Taken together, these findings highlight the multilevel nature of IPV and health outcomes, demonstrating how individual behaviours, interpersonal dynamics, and broader structural conditions—such as public health measures—interact to shape survivor experiences during crises. This evidence reinforces the longstanding advocacy of the VAW sector that IPV must be built into the design of emergency responses from the outset, not retrofitted after the harm has been done.

VAW organizations themselves also require dedicated emergency planning and support within their organizations — the findings showed that even within shelter environments, pandemic protocols introduced new challenges including isolation, restricted movement, and delayed detection of escalating substance use and psychological distress among residents. Ensuring that shelters and VAW organizations have the resources, flexibility, institutional backing and emergency preparedness to adapt their operations during a crisis is essential to maintaining continuity of care for the most vulnerable survivors. The finding that many survivors lacked awareness of available services — itself a significant predictor of poor physical health in this study — further underscores the need for proactive, community-embedded outreach as a core component of emergency planning, particularly to reach first-time survivors who may never have engaged with formal support [9]. This lack of awareness may have been compounded by pandemic-related service disruptions and closures, which reduced visibility and accessibility of supports.

### Limitations

This study has several limitations. The cross-sectional design of the quantitative survey limits causal inference and relies on retrospective self-report, which may introduce recall and social desirability biases. Although many survey items were adapted from validated measures, the self-perceived nature of mental and physical health outcomes does not necessarily reflect clinical assessments. Importantly, participants were asked to recall and compare their health behaviours and outcomes from a period approximately two to three years prior to data collection. Recalling nuanced changes across behavioural, psychological, and physiological domains over an extended and emotionally turbulent period introduces meaningful potential for recall bias. Participants may have difficulty accurately distinguishing pre-pandemic baselines from lockdown-period experiences, and the emotional weight of the pandemic period may further shape how those experiences are remembered and reported. These factors should be considered when interpreting the magnitude and direction of reported changes.

While Bonferroni correction was applied to the descriptive bivariate analyses, formal correction for multiple testing was not applied across the regression models. Each model was estimated separately for a distinct health outcome and should be interpreted accordingly. Findings with p-values approaching conventional thresholds should be interpreted with caution.

The categorization of participants as “women without IPV experience” refers specifically to those who did not report experiencing IPV during the COVID-19 lockdown period. It is possible that some participants in this group may have experienced IPV prior to the pandemic. Prior experiences of violence may influence current coping strategies, mental health, and health behaviours, which could obscure observed differences between groups and limit the comparability of the IPV and non-IPV groups. Furthermore, the non-IPV group was not screened for other forms of adversity or trauma experienced during the lockdown period, meaning that some participants in this group may have faced other stressors not included in this study that affect health outcomes independently of IPV. The decision to focus on IPV exposure during the lockdown period specifically reflects the study’s aim to examine how pandemic-related restrictions intersected with experiences of violence and health outcomes at that time. These limitations in group comparability should be considered when interpreting between-group differences.

Although the survey sample was diverse across several sociodemographic variables, the qualitative interviews included only women IPV survivors. This excludes the perspectives of survivors with other gender identities and may overrepresent individuals with stronger connections to shelter or service networks. Recruitment through partner organizations may also have introduced selection bias toward participants already engaged with support services. Because only women IPV survivors were interviewed, the mixed-methods analysis was restricted to the 653 women participants rather than the full sample of 1,344 survey respondents. Although IPV affects individuals of all genders, the qualitative findings reflect only the experiences of those who self-identified as women.

While the mixed-methods design enabled triangulation of findings, full integration was constrained by differences in sample composition and timing of data collection. Despite these limitations, this study offers important insights into how pandemic-related disruptions intersected with IPV to exacerbate health inequities, guided by theoretical frameworks and shaped by community engagement throughout the research process. This study affirms the value of community-engaged research in generating findings that are not only academically rigorous but directly actionable, grounded in the realities of frontline practice.

## Conclusion

This mixed-methods study demonstrates that women who experienced IPV during COVID-19 lockdowns in Ontario were significantly more likely to report adverse behavioural, psychological, and physiological health outcomes compared to women who did not. These findings provide empirical evidence that public health emergencies disproportionately burden survivors of IPV and underscore the urgent need for crisis planning and policy responses that are trauma-informed, equity-oriented, and grounded in the lived experience of survivors. By documenting the specific health impacts of IPV during a global crisis, this study contributes to the broader evidence base supporting health equity for marginalized populations. It also offers actionable insights to inform emergency preparedness, disaster response, and pandemic planning—ensuring that IPV survivors are not overlooked in times of crisis.

## Supplementary Information

Additional file 1. GRAMMS Checklist: Reporting of Mixed Methods in this Study.

Additional file 2. Semi-structured interview guides created for this study, including one for IPV survivors and another for VAW service providers.

Additional file 3. Variable Coding and Operationalization.

Additional file 4. STATA Syntax for data analysis.

Additional file 5. Detailed Descriptive and Analytical Results Tables.

Additional file 6. Propensity Score Matching Sensitivity Analysis.

## Data Availability

The qualitative and quantitative datasets generated during this study are not publicly available due to their sensitive nature involving intimate partner violence experiences and the need to protect participant privacy as guaranteed during informed consent. Anonymized data may be available from the corresponding author upon reasonable request and subject to appropriate ethical and confidentiality safeguards.

## Abbreviations

aOR: adjusted odds ratio
CIHI: Canadian Institute for Health Information
CI: confidence interval
COVID-19: Coronavirus Disease 2019
GBV: gender-based violence
GRAMMS: Good Reporting of a Mixed Methods Study
GTA: Greater Toronto Area
HPA: hypothalamic-pituitary-adrenal
IPV: intimate partner violence
LEO: LEO powered by Leger (survey research firm)
LGBTQ2+: Lesbian, Gay, Bisexual, Transgender, Queer, Two-Spirit, and others
OAITH: Ontario Association of Interval and Transition Houses
OR: odds ratio
PAG: project advisory group
PP: predicted probability
PTSD: Post-Traumatic Stress Disorder
RRR: relative risk ratio
SLE: stressful life events
UN: United Nations
VAW: violence against women
WHO: World Health Organization
