# Supplementary material for: “We were locked in with our trauma” - a mixed-methods study of health pathways among intimate partner violence (IPV) survivors during COVID-19 lockdowns in Ontario
Source: BMC Public Health. 2026 Jun 19;26:1924. doi: 10.1186/s12889-026-28032-6 (PMC13282876; doi:10.1186/s12889-026-28032-6)
Supplement: Supplementary file 1 — Additional file 1. GRAMMS Checklist: Reporting of Mixed Methods in this Study. [file 12889_2026_28032_MOESM1_ESM.docx]

**Additional File 1**

**GRAMMS Checklist: Reporting of Mixed Methods in this Study** [1]

(Based on: O’Cathain et al., 2008)

| **GRAMMS Reporting Criterion** | **Description of How Criterion Was Addressed in This Study** |
| --- | --- |
| 1. Justification for using a mixed methods approach | A mixed methods approach was used to explore and compare the experiences of IPV survivors and non-survivors during the COVID-19 lockdowns. The integration of quantitative survey data and qualitative interviews provided both breadth (population-level comparisons) and depth (rich survivor and service provider narratives), allowing for a more comprehensive understanding of health inequities. |
| 2. Description of the design in terms of the purpose, priority, and sequence of methods | The study employed a convergent design where qualitative and quantitative data were collected simultaneously, analyzed separately, and then integrated. Neither method was prioritized over the other; both were treated as equally important for understanding health outcomes and pathways. |
| 3. Description of each method in terms of sampling, data collection, and analysis | The quantitative component included a cross-sectional survey (n=653 women) collected through LEO (Leger), using quota sampling. Analysis included descriptive stats, chi-square tests, and logistic regression. The qualitative component included semi-structured interviews with 14 IPV survivors and 10 service providers, using convenience sampling and thematic analysis following Braun & Clarke. |
| 4. Description of where integration has occurred and how | Integration occurred at the interpretation and reporting stages. Quantitative results identified patterns in behavioural, psychological, and physiological health pathways; qualitative themes provided explanations and real-world context. Triangulation was used to confirm and expand upon findings from each strand. |
| 5. Description of any limitations of the mixed methods design | Limitations included the cross-sectional nature of the quantitative data, potential recall bias, and the fact that only women participated in the interviews, limiting generalizability to other genders. Integration was limited by differences in sample sizes and timing of data collection. |
| 6. Description of insights gained from mixing methods | Mixed methods enabled a more nuanced understanding of how COVID-19 amplified health inequities for IPV survivors. Quantitative results demonstrated disparities in health outcomes; qualitative data explained underlying mechanisms such as stigma, isolation, caregiving strain, and informational barriers. This added context enriched interpretation and provided more actionable implications for policy and emergency planning. |

1. O’cathain A, Murphy E, Nicholl J. The Quality of Mixed Methods Studies in Health Services Research. J Health Serv Res Policy. 2008 Apr 1;13(2):92–8.
