# Supplementary material for: “We were locked in with our trauma” - a mixed-methods study of health pathways among intimate partner violence (IPV) survivors during COVID-19 lockdowns in Ontario
Source: BMC Public Health. 2026 Jun 19;26:1924. doi: 10.1186/s12889-026-28032-6 (PMC13282876; doi:10.1186/s12889-026-28032-6)
Supplement: Supplementary file 2 — Additional file 2. Semi-structured interview guides created for this study, including one for IPV survivors and another for VAW service providers. [file 12889_2026_28032_MOESM2_ESM.docx]

**Additional File 2**

**Semi-Structured Interview Guide 1**

**Individuals who Experienced IPV during COVID-19 Lockdowns in Ontario**

**Introduction**

Thank you for taking the time to meet with me today and sharing your experiences. Before we begin, is it safe to talk/answer questions for the next 30-60mins? Please know you can hang up at any point and feel free to connect with me when it is safe to continue.

My name is Dina Idriss-Wheeler, and I would like to speak with you today about your experience of intimate partner violence during the pandemic lockdown periods between March 2020 and June 2021 while you were living in Ontario.

Before we begin, I would like to ask you if you had any questions about the consent form that was sent. I would like to seek verbal consent; do you agree to participate in this research study?

I understand that these questions are of a sensitive nature and may trigger emotional or psychological distress. Please know that we can stop at any time and I have shared and can share resources. Potential supports and my contact information are in the chat feature and I am happy to resend them to you.

List of resources*:*

- Assaulted Women's Helpline via 1-888-388-2915 (24 hours a day) or their online chat via: https://www.awhl.org/online-chat 11am-8pm Monday-Friday EST
- Talk 4 Healing offers confidential support 24 hours a day in 14 Indigenous languages, French, and English via 1-855-554-HEAL.
- Femaide provides support 24 hours a day to Francophone survivors via 1-877-336-2433
- Find your closest violence against women service via www.sheltersafe.ca
- Didri040@uottawa.ca

I would also like to seek your permission to audio-record this interview to ensure I have captured all the details. Please know that your responses will be confidential, and no personal identifiers will be reported. You can also stop this interview at any moment. Please also feel free to turn off your video. Please note that only the audio will be kept for transcription, the video will be destroyed.

Finally, I am obliged to let you know that I have a legal responsibility to disclose information on child abuse and neglect under the Ontario Child and Family Services Act.

Under the Act, if there is something worrying regarding child abuse and neglect from an abuser in the household, I have to contact Children’s Aid Society (CAS), even when the information is confidential or privileged. If, during the interview, information is disclosed that a child under the age of 18 is in danger of abuse or neglect from the abusive person in the home, a consult with experts (Project Advisory Committee of violence against women organizations) in the field will take place to determine disclosure to CAS. If the team of experts determines a report is necessary, the researcher will have to disclose that information to CAS.

Do you have any questions for me before we begin the session and recording?

If you’d like to turn off your camera now, you are welcome to.

**About the participant**

1. Can you please tell me a little bit about yourself? [probing questions about age, employment, income, education, number of children/dependents, partnership status & dynamics]
2. What was your motivation to participate in this study?

**Experience of IPV before and during COVID-19 lockdowns**

1. You contacted me about this study on intimate partner violence. Can you please tell me about your experience with IPV before and then after COVID? Please let me know if you need to stop at any point in time:

- Did you experience any physical, sexual, mental, emotional, financial, controlling, or coercive force from your partner before lockdown? [will give examples of each] Can you talk about the experience? [i.e., did it happen daily, weekly, monthly].
- How did this change during COVID lockdowns? (worse, better, stayed the same) Can you tell me more about what changed and why you think it changed?
- Prompt: were the lockdowns used as a threat? If yes, how? Can you tell me about having to wear masks; did this play any role (i.e., covering up bruises)?
- Did you or your partner lose your job and how did things shift for you in terms of experience of violence after this happened?

1. Can you please comment on your partner’s alcohol drinking or other forms of substance use both before and after COVID lockdowns, and how it related to your experience of IPV?

**Exploring how COVID-19 shaped how VAW clients accessed social supports (i.e., formal and informal supports)**

The next set of questions are about your experience and if you accessed formal services (i.e., violence against women shelters/social supports or healthcare organizations or helplines) or informal supports (family, friends, neighbours).  I will start with formal services:

1. Can you please tell me about your experience of trying to access any violence against women support services (i.e., shelters, crisis lines crisis response, health care) during lockdown? If you did not, did you know about them or how to access them?
2. *Alternative Housing (hotel, relative, private, and donated institutions) –* If you did access them during COVID, were you placed in alternative housing? If yes, what did you like or not like about alternative housing? Can you comment about how you felt in terms of safety/security from your partner? How about safety in terms of health, did this play a role in accessing the these services for you (i.e., living during pandemic)? What would have helped, if anything?
3. What would you say were the ***challenges/barriers/problems that you faced*** (if any) to accessing formal support services VAW shelters, crisis lines, healthcare)  during lockdown?
4. What do you think would have helped you to access the support services (i.e., VAW shelters, crisis lines, healthcare) during lockdown?
5. What was your experience, if any, to accessing any other organizations to seek advice, help, support for your experience of IPV? For example, did you access (or attempt to access) faith-based supports (i.e., church, mosque, temple) or social supports available in your area during lockdown (community centres)?”
6. Do you think you had enough information about what types of services for violence against women are available to you during lockdown and how to access them? If not, do you have suggestions for how best to get this information to you?
7. Can you please talk to me about how school closures and/or work from home orders affected you? Did the violence increase when you worked at home?

Now I am shifting to talking about informal supports – so contacting family, friends, neighbours…

1. What was your experience with contacting or connecting with any family, friends, or neighbours during the lockdown? Did this change from how it was before COVID? [Prompt: experience of isolation? face-to-face contact? virtual contact?]?

1. Do you have any suggestions or is there anything that would have made it easier for you to access family or friends – something you can share from this experience which you think would be helpful in other emergency/disaster contexts or situations or future pandemics to help you access services or your social network of family/friends/neighbours?

**Exploring perceived behavioural, psychological, and physiological health of women who experienced IPV in during COVID-19 lockdowns in Ontario**

The next series of questions asks about you felt physical and mentally…in terms of your behaviours and health (mental and physical) and if anything changed during COVID-19 lockdowns. I found it challenging to exercise and eat well during lockdown.

1. Can you tell me a story or talk about any changes in your health behaviours – did you find you exercised less or more, slept less, ate more junk, drank more alcohol or smoked more….what changed for you during COVID-19 lockdowns?
2. Why do you think it changed?
3. Did you try to seek help for any of this?
4. What was your experience and what would have helped?

1. Can you talk about or provide a story regarding change in your mental health (i.e., coping, depression/distress, emotional regulation, sense of well-being; PTSD)? What was tough for you, how did you find it mentally to deal with things during this time?
2. Did you try to seek help for any of this?
3. What was your experience and what would have helped?

1. Can you talk about or provide a story regarding change in your overall physical health (i.e., stress overload, immune function reduced/getting sick more often, physical injury)?
2. Did you try to seek help for any of this?
3. What was your experience and what would have helped?

**Experience of Violence currently, as we move into pandemic recovery**

1. Can you tell me how things are for you now as we move into recovery phase of the pandemic?
2. Are you experiencing any barriers to accessing either social or health or community supports or services?
3. Would you say things are different than before the pandemic?

**Closing Questions**

1. Is there anything else you would like to share with me?
2. How did you think that went?
3. Do you have any questions for me?

I know that this is hard, and it is so generous of you to share your story with me. Please know it is a privilege and I reassure you that this work is being done to make a change. Your resilience is inspiring, and I thank you very much for taking the time to share your perspectives with me.

I have posted support resources in the chat and I can send them to you in any way that is easy for you. I also have a $50 compensation to give you – it can be in the form of e-transfer, gift card. How would you like to receive it?

Finally, would you be interested in learning about the findings of this research? If yes, do you consent to my using your contact email to communicate research related findings?

**Semi-Structured Interview Guide 2**

**Violence Against Women (VAW) Service Organization Representative**

**Introduction**

Thank you for taking the time to meet with me today. My name is Dina Idriss-Wheeler, and I would like to speak with you today about your experience as a VAW service provider, in Ontario, during the pandemic lockdown periods between March 2020 and June 2021. This interview will take between 45 mins to an hour. Before we begin, I would like to ask you if you had any questions about the consent form that was sent [if signed form was not submitted before interview, verbal consent will be sought at this time by asking participants to state if they agree to participate in this research study]. I would also like to seek your permission to audio-record this interview to ensure I have captured all the details.

I understand that these questions are of a sensitive nature and may trigger emotional or psychological distress. Please know that we can stop at any time and I have shared and can share resources to help. Potential supports and my contact information are in the chat feature and I am happy to resend them to you.

Please know that your responses will be confidential, and no personal identifiers will be reported. You can also stop this interview at any moment.

Do you have any questions for me before we begin?

**Context of VAW organization before COVID-19 pandemic**

1. Can you please tell me about yourself and your role at [Insert VAW organization name]?
2. What were the main programs and services provided by your organization prior to COVID-19?
3. Can you please describe the client base served by your organization prior to COVID-19?

**Exploring how COVID-19 affected VAW service provision**

1. Did your role change after the COVID-19 lockdown measures were implemented? If so, how?
2. After lockdown measures were implemented in Ontario, what changes did you see in terms of clients seeking your services?
3. If you provided shelters, were you above, at or below capacity at any point in time during lockdowns? Please explain.
4. Did you have to implement alternative housing options as part of your services during COVID-19 (i.e., hotels, relatives’ houses, private rentals such as university/municipality facilities)? Please explain.
5. What are the barriers to implementing alternative housing? Can you suggest strategies that would help decrease risks associated with using alternative housing options or to help remove the barriers in case of future crisis/disasters/pandemics?
6. Did you notice if the socio-demographics of your clients changed during COVID-19 (i.e., race, age, ethnicity, immigrant/refugee status)?
7. What were the ***challenges/barriers*** to providing your services? From your perspective what were the challenges/barriers to reaching your clients?

*Prompt: what are some cultural barriers that impeded women from accessing services?*

1. What were the ***facilitators/key strategies*** that helped you provide your services and reach your clients?

*Prompt: how did technology impact or shift service provision? Did you have resources to provide services? Did clients have devices to access online services & know how to use them?*

1. From your perspective, what new strategies (if any) do you think will continue to be used after we “return to normal”?
2. Do you have lesson learned that you can share from this experience you think would be helpful in other emergency/disaster contexts or situations or future pandemics?

**Exploring Perspectives of VAW representatives regarding impact of COVID-19 lockdown on behavioural, psychological, and physiological health of women who experienced IPV in Ontario.**

1. From your perspective, did physical distancing measures make it difficult to provide your services? [skip if addressed above]
2. Isolation is a key factor in the ability of clients to access services and your organization’s ability to reach clients. From your perspective, can you please comment on the role isolation played in your ability to reach your clients and for them to access services, both before and then during COVID-19 lockdown?
3. From your perspective, can you comment or provide a story regarding changes in health behaviours (i.e., substance use, physical exercise, help seeking, reduced sleep, unhealthy diets) of clients due to COVID-19 lockdowns?
4. From your perspective, can you comment or provide a story regarding change in your clients’ mental health (i.e., coping, depression/distress, emotional regulation, sense of wellbeing; PTSD)?
5. From your perspective, can you comment or provide a story regarding change in overall physical health of the clients served by your organization (i.e., stress overload, immune function reduced/getting sick more often, physical injury)?

**Closing questions**

1. Is there anything else you would like to share with me?
2. Do you have any questions for me?

Thank you very much for taking the time to share your perspectives with me. Would you be interested in learning about the findings of this research? If yes, do you consent to my using your contact email to communicate research-related findings?
