# Supplementary material for: “We were locked in with our trauma” - a mixed-methods study of health pathways among intimate partner violence (IPV) survivors during COVID-19 lockdowns in Ontario
Source: BMC Public Health. 2026 Jun 19;26:1924. doi: 10.1186/s12889-026-28032-6 (PMC13282876; doi:10.1186/s12889-026-28032-6)
Supplement: Supplementary file 3 — Additional file 3. Variable Coding and Operationalization. [file 12889_2026_28032_MOESM3_ESM.docx]

**Additional File 3**

**Variable Coding and Operationalization**

This supplementary file provides a detailed overview of the coding procedures and variable construction used in this study. The aim is to ensure transparency and replicability of the analyses. We describe how the primary outcome variables—self-reported poor mental and physical health—were operationalized using participants’ retrospective and contemporaneous assessments of their health before and during the COVID-19 lockdown period. We also outline the coding of covariates informed by existing literature and community consultation, including variables related to caregiving roles, community violence, service access knowledge, and substance use. These details complement the main manuscript by offering greater clarity on how key constructs were derived and categorized for analysis.

**Outcome Variables: Self-Reported Poor Mental and Physical Health**

Two binary outcome variables—*poor mental health* and *poor physical health*—were created to capture participant self-reported health status during the COVID-19 pandemic lockdowns. Prior research supports the validity of using self-reported health measures during the pandemic period.[1,2]

Participants were first asked about their **pre-pandemic** health:

- *“Would you say, in general, that your physical health—which includes illness and injury—before COVID-19 lockdowns (March 2020–June 2021) was:”*
- *“Would you say, in general, that your mental health—which includes stress, depression, anxiety, and problems with emotions—before COVID-19 lockdowns (March 2020–June 2021) was:”*

Response options: *excellent, very good, good, fair,* or *poor.*

They were then asked about their health **during the lockdowns**:

- *“Thinking about your overall physical health—which includes illness and injury—how did COVID-19 lockdowns (March 2020–June 2021) affect your physical health? My physical health became...”*
- *“Thinking about your overall mental health—which includes stress, depression, anxiety, and problems with emotions—how did COVID-19 lockdowns (March 2020–June 2021) affect your mental health? My mental health became...”*

Response options: *much better, better, about the same, worse,* or *much worse.*

These responses were used to classify participants into binary outcome groups:

- **Bad Mental Health** (coded as 1):
  - If the participant reported their mental health was *“About the Same”* during COVID-19 but had been rated as *“Poor”* pre-pandemic,
  - Or if they reported their mental health was *“Worse”* or *“Much Worse”* during the pandemic.
- **Good Mental Health** (coded as 0):
  - If their mental health was *“About the Same”* during COVID-19 but rated as *“Excellent,” “Very Good,” “Good,”* or *“Fair”* pre-pandemic,
  - Or if they reported their mental health was *“Better”* or *“Much Better”* during COVID-19.
- **Bad Physical Health** (coded as 1):
  - If the participant reported their physical health was *“About the Same”* during COVID-19 but had been rated as *“Poor”* pre-pandemic,
  - Or if they indicated their physical health was *“Worse”* or *“Much Worse”* during the pandemic.
- **Good Physical Health** (coded as 0):
  - If physical health was *“About the Same”* during COVID-19 but had been rated as *“Excellent,” “Very Good,” “Good,”* or *“Fair”* before COVID-19,
  - Or if they reported their physical health was *“Better”* or *“Much Better”* during the pandemic.

Missing data in either pre- or during-COVID-19 health variables were coded as missing for both outcomes.

**Covariates**

The following covariates are not the primary independent variable (IPV), but were selected based on literature and consultation with the PAG, given their known influence on health outcomes.

- **Community Violence**
  Exposure to community violence has been associated with adverse physical and mental health outcomes.[3–5] Participants were asked:
  *“Do you think community violence was a problem where you lived during the pandemic (March 2020–June 2021)?”*
  (Response options: *Yes/No*)
- **Caregiving Roles**
  IPV and caregiving have both been linked to worse physical and mental health outcomes.[6] Informal caregiving was measured by:
  - Self-reported change in caregiving roles (*increased, decreased, no change*)
  - Whether the participant was the **primary informal caregiver** for children or dependents (*yes/no*)
  - Number of children cared for
- **Service Access Knowledge**
  Participants were asked whether they felt adequately informed about available services and how to access them during lockdowns (*yes/no*). Satisfaction with health information has been shown to be protective against stress, anxiety, and depression.[7,8]
- **Substance Use Impact Score**
  Given the role of substance use in IPV dynamics,[9,10] during pandemic lockdowns,[11] and its association with mental and physical health outcomes,[12] a composite measure—**Substance Use Impact Score**—was created.

This score combines responses to the following items:

- - “I wished my partner would not drink so many drinks/use substances.”
  - “My partner’s drinking/substance use is a source of strain in our relationship.”
  - “I considered leaving because of drinking/substance use.”

Responses were dichotomized:

- - *Always/Often/Sometimes* = 1 (presence of stressor)
  - *Never/Seldom* = 0 (absence)

The sum of these binary variables generated a total score, which was categorized as:

- - Low Impact (score ≤ 2)
  - Moderate/High Impact (score > 2)

**References:**

1. Moniuszko-Malinowska A, Czupryna P, Dubatówka M, Łapińska M, Kazberuk M, Szum-Jakubowska A, et al. COVID-19 pandemic influence on self-reported health status and well-being in a society. Sci Rep. 2022 May 24;12(1):8767.

2. Moser N, Sahiti F, Gelbrich G, Cejka V, Kerwagen F, Albert J, et al. Association between self-reported and objectively assessed physical functioning in the general population. Sci Rep. 2024 Jul 14;14(1):16236.

3. CDC. Community Violence Prevention. 2024 [cited 2025 Jan 24]. About Community Violence. Available from: https://www.cdc.gov/community-violence/about/index.html

4. Miliauskas CR, Faus DP, da Cruz VL, do Nascimento Vallaperde JGR, Junger W, Lopes CS. Community violence and internalizing mental health symptoms in adolescents: A systematic review. BMC Psychiatry. 2022 Apr 9;22(1):253.

5. Wright AW, Austin M, Booth C, Kliewer W. Systematic Review: Exposure to Community Violence and Physical Health Outcomes in Youth. Journal of Pediatric Psychology. 2017 May 1;42(4):364–78.

6. Ferreira P, Loxton D, Tooth LR. Intimate personal violence and caregiving: Influences on physical and mental health in middle-aged women. Maturitas. 2017 Aug 1;102:34–40.

7. Xiong J, Lipsitz O, Nasri F, Lui LMW, Gill H, Phan L, et al. Impact of COVID-19 pandemic on mental health in the general population: A systematic review. J Affect Disord. 2020 Dec 1;277:55–64.

8. Wang C, Pan R, Wan X, Tan Y, Xu L, McIntyre RS, et al. A longitudinal study on the mental health of general population during the COVID-19 epidemic in China. Brain, Behavior, and Immunity. 2020 Jul 1;87:40–8.

9. Heise LL. Determinants of partner violence in low and middle-income countries : exploring variation in individual and population-level risk [Internet] [doctoral]. London School of Hygiene & Tropical Medicine; 2012 [cited 2021 May 11]. Available from: https://researchonline.lshtm.ac.uk/id/eprint/682451/

10. Smith PH, Homish GG, Leonard KE, Cornelius JR. Intimate partner violence and specific substance use disorders: Findings from the National Epidemiologic Survey on Alcohol and Related Conditions. Psychol Addict Behav. 2012 Jun;26(2):10.1037/a0024855.

11. Hartney E. The Shadow Pandemic of Alcohol Use during COVID-19: A Canadian Health Leadership Imperative. Healthc Policy. 2021 May;16(4):17–24.

12. CDC. Alcohol Use. 2025 [cited 2025 Jan 24]. Alcohol Use and Your Health. Available from: https://www.cdc.gov/alcohol/about-alcohol-use/index.html
