# Supplementary material for: “We were locked in with our trauma” - a mixed-methods study of health pathways among intimate partner violence (IPV) survivors during COVID-19 lockdowns in Ontario
Source: BMC Public Health. 2026 Jun 19;26:1924. doi: 10.1186/s12889-026-28032-6 (PMC13282876; doi:10.1186/s12889-026-28032-6)
Supplement: Supplementary file 4 — Additional file 4. STATA Syntax for data analysis. [file 12889_2026_28032_MOESM4_ESM.pdf]

```
**Additioinal File 4**

*STATA Syntax for data analysis*

*************************
*CREATING CODEBOOK*
*************************

*DROPPING CASES*
label variable DROP "Cases to Drop"
label define casestodrop 1 "DROP" 0 "Keep"
label values DROP casestodrop
codebook DROP

*Language of Survey*
label variable LANG "Language the Survey was taken"
label define langtaken 1 "English" 2 "French"
label values LANG langtaken
codebook LANG

*EXPERIENCE OF IPV*
label variable IPV_EXP "Experience of IPV During COVID"
label define IPV 1 "YesIPV" 2 "NoIPV"
label values IPV_EXP IPV
codebook IPV_EXP

*HEALTH BEFORE*
label variable PHYS_B4_COVID "Physical_Health_B4_COVID"
label define PHYSB4 1 "Excellent" 2 "Very good" 3 "Good" 4 "Fair" 5 "Poor" 0 "Missing"
label values PHYS_B4_COVID PHYSB4
codebook PHYS_B4_COVID

label variable MENTAL_B4_COVID "Mental_Health_B4_COVID"
label define MENTB4 1 "Excellent" 2 "Very good" 3 "Good" 4 "Fair" 5 "Poor" 0 "Missing"
label values MENTAL_B4_COVID MENTB4
codebook MENTAL_B4_COVID

*HEALTH DURING*
label variable PHYS_IN_COVID "Physical_Health_IN_COVID"
label define PHYSIN 1 "MuchBetter" 2 "Better" 3 "About the Same" 4 "Worse" 5 "MuchWorse" 0 "Missing"
label values PHYS_IN_COVID PHYSIN
codebook PHYS_IN_COVID

label variable MENTAL_IN_COVID "Mental_Health_IN_COVID"
label define MENTIN 1 "MuchBetter" 2 "Better" 3 "About the Same" 4
```

```
"Worse" 5 "MuchWorse" 0 "Missing"
label values MENTAL_IN_COVID MENTIN
codebook MENTAL_IN_COVID

*HEALTH POST*
label variable PHYSICAL_POST "Physical_Health_POST_COVID"
label define PHYSPOST 1 "MuchBetter" 2 "Better" 3 "About the Same" 4 "Worse" 5 "MuchWorse" 0 "Missing"
label values PHYSICAL_POST PHYSPOST
codebook PHYSICAL_POST

label variable MENTAL_POST "Mental_Health_POST_COVID"
label define MENTPOST 1 "MuchBetter" 2 "Better" 3 "About the Same" 4 "Worse" 5 "MuchWorse" 0 "Missing"
label values MENTAL_POST MENTPOST
codebook MENTAL_POST


*HEALTH BEHAVIOURS DURING*
label variable ALCOHOL_DURING "ConsumingAlcoholDuringCOVID"
label define ALCOHinCOV 1 "increased" 2 "decreased" 3 "no change" 0 "Missing"
label values ALCOHOL_DURING ALCOHinCOV
codebook ALCOHOL_DURING

label variable TOBAC_DRUING "ConsumingTobaccoDuringCOVID"
label define TOBACinCOV 1 "increased" 2 "decreased" 3 "no change" 0 "Missing"
label values TOBAC_DRUING TOBACinCOV
codebook TOBAC_DRUING

label variable CANNAB_DURING "ConsumingCannabisDuringCOVID"
label define CANNABinCOV 1 "increased" 2 "decreased" 3 "no change" 0 "Missing"
label values CANNAB_DURING CANNABinCOV
codebook CANNAB_DURING

label variable SUBSTANCE_DURING "ConsumingSubstancesDuringCOVID"
label define SUBSTinCOV 1 "increased" 2 "decreased" 3 "no change" 0 "Missing"
label values SUBSTANCE_DURING SUBSTinCOV
codebook SUBSTANCE_DURING

label variable TV_DURING "WatchingTVDuringCOVID"
label define TVinCOV 1 "increased" 2 "decreased" 3 "no change" 0 "Missing"
label values TV_DURING TVinCOV
codebook TV_DURING
```

```
label variable INTERNET_DURING "TimeOnInternetDuringCOVID"
label define INTERinCOV 1 "increased" 2 "decreased" 3 "no change" 0 "Missing"
label values INTERNET_DURING INTERinCOV
codebook INTERNET_DURING

label variable EXERCISING_DURING "ExercisingDuringCOVID"
label define EXERCinCOV 1 "increased" 2 "decreased" 3 "no change" 0 "Missing"
label values EXERCISING_DURING EXERCinCOV
codebook EXERCISING_DURING

label variable EATJUNK_DURING "EatingJunkDuringCOVID"
label define EATJUNKinCOV 1 "increased" 2 "decreased" 3 "no change" 0 "Missing"
label values EATJUNK_DURING EATJUNKinCOV
codebook EATJUNK_DURING

label variable COMM_FRND_DURING "CommunicatingFriendsDuringCOVID"
label define COMMFRNDinCOV 1 "increased" 2 "decreased" 3 "no change" 0 "Missing"
label values COMM_FRND_DURING COMMFRNDinCOV
codebook COMM_FRND_DURING

label variable COMM_FAM_DURING "CommunicatingFamilyDuringCOVID"
label define COMMFAMinCOV 1 "increased" 2 "decreased" 3 "no change" 0 "Missing"
label values COMM_FAM_DURING COMMFAMinCOV
codebook COMM_FAM_DURING

label variable INFORMAL_DURING "SeekingINFORMALSupportsDuringCOVID"
label define INFORSUPinCOVID 1 "increased" 2 "decreased" 3 "no change" 0 "Missing"
label values INFORMAL_DURING INFORSUPinCOVID
codebook INFORMAL_DURING

label variable FORMAL_DURING "SeekingFORMALSupportsDuringCOVID"
label define FORMSUPinCOVID 1 "increased" 2 "decreased" 3 "no change" 0 "Missing"
label values FORMAL_DURING FORMSUPinCOVID
codebook FORMAL_DURING

label variable SLEEP_DURING "QualityOfSleepDuringCOVID"
label define SLEEPCOVID 1 "increased" 2 "decreased" 3 "no change" 0 "Missing"
label values SLEEP_DURING SLEEPCOVID
codebook SLEEP_DURING
```

```
*CAREGIVING AND CHILDREN*

label variable PRIMARY_CAREGIVER_DURING "Primary_CaregiverDuringCOVID"
label define PRIMARY_CAREGIVER_DURING 1 "Yes" 2 "No" 0 "Missing"
label values PRIMARY_CAREGIVER_DURING CAREGIVINGCOV
codebook PRIMARY_CAREGIVER_DURING

label variable NUM_CHILD "NumberOfChildren_under_17"
label define NumCHILD17 1 "None" 2 "1" 3 "2" 4 "3" 5 "4" 6 ">4"
label values NUM_CHILD NumCHILD17
codebook NUM_CHILD

label variable CAREGIVING_nonCHILD "Primary_CaregiverNon_Child_DuringCOVID"
label define CAREGIVINGNONCHILD 1 "Yes" 2 "No" 0 "Missing"
label values CAREGIVING_nonCHILD CAREGIVINGNONCHILD
codebook CAREGIVING_nonCHILD

label variable CHANGE_CAREGIVING_DURING "ChangeInCaregivingDuringCOVID"
label define CAREGIVCHANGEINCOV 1 "No_stayed_same" 2 "Yes_increased" 3 "Yes_decreased" 0 "Missing"
label values CHANGE_CAREGIVING_DURING CAREGIVCHANGEINCOV
codebook CHANGE_CAREGIVING_DURING

*INCOME/JOBSTATUS CHANGE DURING COVID*

label variable JOB_STATUS_DURING  "Job_status_changeINCOVID"
label define JOBSTATUSCOV 1 "Yes_no_longer_job" 2 "No_kept_job" 0 "Missing"
label values JOB_STATUS_DURING JOBSTATUSCOV
codebook JOB_STATUS_DURING

label variable INCOME_DURING  "ChangIncomeDuringCOVID"
label define INCOMECHANGE 1 "Yes_decreased" 2 "No_stayed_same" 3 "Yes_increased" 0 "Missing"
label values INCOME_DURING INCOMECHANGE
codebook INCOME_DURING

*FACE TO FACE CONTACT VS VIRTUAL*

label variable F2F_CONTACT_DURING  "Face_to_Face_Contact_during_COVID"
label define F2FinCOV 1 "No_Contact_outside_HH" 2 "Yes_1to3_Individ" 3 "Yes_4to8_Individ" 4 "Yes_>8" 5 "Unknown"
label values F2F_CONTACT_DURING F2FinCOV
codebook F2F_CONTACT_DURING
```

```
codebook F2F_CONTACT_DURING

label variable VIRT_CONTACT_DURING  "Virtual_Contact_during_COVID"
label define VIRTinCOV 1 "No_Contact_outside_HH" 2 "Yes_1to3_Individ" 3 "Yes_4to8_Individ" 4 "Yes_>8" 5 "Unknown"
label values VIRT_CONTACT_DURING VIRTinCOV
codebook VIRT_CONTACT_DURING

*SERVICE IN PERSON OR VIRTUAL OR BOTH*

label variable SERVICES_VIRT_INPERS  "Services accessed in person, virtual or both"
label define SERVICEPERSONVIRT 1 "in-person" 2 "Virtual" 3 "both" 4 "DidNotAccessDuringLockdown"
label values SERVICES_VIRT_INPERS SERVICEPERSONVIRT
codebook SERVICES_VIRT_INPERS

*BARRIERS DURING COVID*

label variable BARR_COMMBASE_DURING "Barr_CommunityBasedOrg_DURING_COVID"
label define BARRCBOCOV 1 "Yes" 2 "No" 3 "Did_not_try" 0 "Missing"
label values BARR_COMMBASE_DURING BARRCBOCOV
codebook BARR_COMMBASE_DURING

label variable BARR_COUNS_DURING  "Barr_Counselling_DURING_COVID"
label define BARRCOUNSCOV 1 "Yes" 2 "No" 3 "Did_not_try" 0 "Missing"
label values BARR_COUNS_DURING BARRCOUNSCOV
codebook BARR_COUNS_DURING

label variable BARR_CRISISLINE_DURING  "Barr_CrisisLine_DURING_COVID"
label define BARRCRLineCOV 1 "Yes" 2 "No" 3 "Did_not_try" 0 "Missing"
label values BARR_CRISISLINE_DURING BARRCRLineCOV
codebook BARR_CRISISLINE_DURING

label variable BARR_HEALTHCR_DURING  "Barr_Healthcare_DURING_COVID"
label define BARRHCCOV 1 "Yes" 2 "No" 3 "Did_not_try" 0 "Missing"
label values BARR_HEALTHCR_DURING BARRHCCOV
codebook BARR_HEALTHCR_DURING

label variable BARR_EMER_SHELT_DURING "Barr_Emergency_Shelter_DURING_COVID"
label define BARRSHELTERCOV 1 "Yes" 2 "No" 3 "Did_not_try" 0 "Missing"

label values BARR_EMER_SHELT_DURING BARRSHELTERCOV
codebook BARR_EMER_SHELT_DURING
```

```
codebook BARR_EMER_SHELT_DURING

label variable BARR_SETT_DURING  "Barr_Settlement_Agency_DURING_COVID"
label define BARRSETTAGCOV 1 "Yes" 2 "No" 3 "Did_not_try" 0 "Missing"
label values BARR_SETT_DURING BARRSETTAGCOV
codebook BARR_SETT_DURING

label variable BARR_CHILDPROT_DURING "Barr_ChildProtection_DURING_COVID"
label define BARRCHILDPROCOV 1 "Yes" 2 "No" 3 "Did_not_try" 0 "Missing"
label values BARR_CHILDPROT_DURING BARRCHILDPROCOV
codebook BARR_CHILDPROT_DURING

label variable BARR_LEGAL_DURING  "Barr_Legal_DURING_COVID"
label define BARRLEGCOV 1 "Yes" 2 "No" 3 "Did_not_try" 0 "Missing"
label values BARR_LEGAL_DURING BARRLEGCOV
codebook BARR_LEGAL_DURING

label variable BARR_MENT_HC_DURING "Barr_MentalHealth_DURING_COVID"
label define BARRMENTHCOV 1 "Yes" 2 "No" 3 "Did_not_try" 0 "Missing"
label values BARR_MENT_HC_DURING BARRMENTHCOV
codebook BARR_MENT_HC_DURING

label variable BARR_ADDICT_DURING "Barr_AddictionServices_DURING_COVID"
label define BARRADDICTCOV 1 "Yes" 2 "No" 3 "Did_not_try" 0 "Missing"
label values BARR_ADDICT_DURING BARRADDICTCOV
codebook BARR_ADDICT_DURING

label variable BARR_HOUSING_DURING "Barr_HOUSING_DURING_COVID"
label define BARRHOUSCOV 1 "Yes" 2 "No" 3 "Did_not_try" 0 "Missing"
label values BARR_HOUSING_DURING BARRHOUSCOV
codebook BARR_HOUSING_DURING

label variable BARR_TRANS_DURING "Barr_Transportation_DURING_COVID"
label define BARRTRANSPCOV 1 "Yes" 2 "No" 3 "Did_not_try" 0 "Missing"
label values BARR_TRANS_DURING BARRTRANSPCOV
codebook BARR_TRANS_DURING

*Barriers POST COVID*

*label variable BARR_COMMBASE_POST "Barr_CommunityBasedOrg_POST_COVID"
```

```
"Barr_CommunityBasedOrg_POST_COVID"
*label define BARRCBOPOST 1 "Yes" 2 "No" 3 "Did_not_try" 0 "Missing"
*label values BARR_COMMBASE_POST BARRCBOPOST
*codebook BARR_COMMBASE_POST

*label variable BARR_COUNS_POST  "Barr_Counselling_POST_COVID"
*label define BARRCOUNSPOST 1 "Yes" 2 "No" 3 "Did_not_try" 0 "Missing"
*label values BARR_COUNS_POST BARRCOUNSPOST
*codebook BARR_COUNS_POST

*label variable BARR_CRISISLIN_POST  "Barr_CrisisLine_POST_COVID"
*label define BARRCRLinePOST 1 "Yes" 2 "No" 3 "Did_not_try" 0 "Missing"
*label values BARR_CRISISLIN_POST BARRCRLinePOST
*codebook BARR_CRISISLIN_POST

*label variable BARR_HEALTHC_POST  "Barr_Healthcare_POST_COVID"
*label define BARRHCPOST 1 "Yes" 2 "No" 3 "Did_not_try" 0 "Missing"
*label values BARR_HEALTHC_POST BARRHCPOST
*codebook BARR_HEALTHC_POST

*label variable BARR_EMER_SHELT_POST "Barr_Emergency_Shelter_POST_COVID"
*label define BARRSHELTERPOST 1 "Yes" 2 "No" 3 "Did_not_try" 0 "Missing"
*label values BARR_EMER_SHELT_POST BARRSHELTERPOST
*codebook BARR_EMER_SHELT_POST

*label variable BARR_SETT_POST  "Barr_Settlement_Agency_POST_COVID"
*label define BARRSETTAGPOST 1 "Yes" 2 "No" 3 "Did_not_try" 0 "Missing"
*label values BARR_SETT_POST BARRSETTAGPOST
*codebook BARR_SETT_POST

*label variable BARR_CHILDPROT_POST  "Barr_ChildProtection_POST_COVID"
*label define BARRCHILDPROPOST 1 "Yes" 2 "No" 3 "Did_not_try" 0 "Missing"
*label values BARR_CHILDPROT_POST BARRCHILDPROPOST
*codebook BARR_CHILDPROT_POST

*label variable BARR_LEGAL_POST  "Barr_Legal_POST_COVID"
*label define BARRLEGPOST 1 "Yes" 2 "No" 3 "Did_not_try" 0 "Missing"
*label values BARR_LEGAL_POST BARRLEGPOST
*codebook BARR_LEGAL_POST
```

```
*label variable BARR_MENTAHC_POST "Barr_MentalHealth_POST_COVID"
*label define BARRMENTHPOST 1 "Yes" 2 "No" 3 "Did_not_try" 0 "Missing"
*label values BARR_MENTAHC_POST BARRMENTHPOST
*codebook BARR_MENTAHC_POST

*label variable BARR_ADDICT_POST "Barr_AddictionServices_POST_COVID"
*label define BARRADDICTPOST 1 "Yes" 2 "No" 3 "Did_not_try" 0 "Missing"
*label values BARR_ADDICT_POST BARRADDICTPOST
*codebook BARR_ADDICT_POST

*label variable BARR_HOUS_POST "Barr_HOUSING_POST_COVID"
*label define BARRHOUSPOST 1 "Yes" 2 "No" 3 "Did_not_try" 0 "Missing"
*label values BARR_HOUS_POST BARRHOUSPOST
*codebook BARR_HOUS_POST

*label variable BARR_TRANSP_COVID "Barr_Transportation_POST_COVID"
*label define BARRTRANSPOST 1 "Yes" 2 "No" 3 "Did_not_try" 0 "Missing"
*label values BARR_TRANSP_COVID BARRTRANSPOST
*codebook BARR_TRANSP_COVID


*WERE SERVICES HELPFUL*

label variable HELPFUL_DURING "Were_services_Helpful"
label define SERVHELPCOV 1 "Yes" 2 "No" 3 "Did_not_try" 0 "Missing"
label values HELPFUL_DURING SERVHELPCOV
codebook HELPFUL_DURING

*Information during COVID*

label variable INFORMATION_DURING "Information_during_COVID"
label define INFORMinCOVID 1 "Yes" 2 "No" 0 "Missing"
label values INFORMATION_DURING INFORMinCOVID
codebook INFORMATION_DURING

*Accessed BENEFITS DURING COVID**

label variable ACCESS_BENEFIT_DURING "ACCESSED_Financial_Benefits_during_COVID"
label define BENEFITSinCOVID 1 "Yes_applied_unsuccessfull" 2 "Yes_applied_successful" 3 "No_did_not_apply" 0 "Missing"
label values ACCESS_BENEFIT_DURING BENEFITSinCOVID
codebook ACCESS_BENEFIT_DURING
```

```

**DEMOGRPHAIC DATA**

*Gender*
label variable GENDER_PARTICIPANT "Participant_Gender"
label define GENDERPART 1 "Woman" 2 "Man" 3 "Gender_Non_Conforming" 4 "Transgender" 5 "Trans Woman" 6 "Trans Man" 7 "Gender Fluid" 8 "PrefNotSay" 0 "Missing"
label values GENDER_PARTICIPANT GENDERPART
codebook GENDER_PARTICIPANT

label variable GENDER_PARTNER "Partner_Gender"
label define GENDERPARTNER 1 "Man" 2 "Woman" 3 "Gender_Non_Conforming" 4 "Transgender" 5 "Trans Woman" 6 "Trans Man" 7 "Gender Fluid" 8 "PrefNotSay" 0 "Missing"
label values GENDER_PARTNER GENDERPARTNER
codebook GENDER_PARTNER

*Age*

label variable AGE_PART "Age_Participant"
label define AGEPARTICIPANT 1 "18-24" 2 "25-34" 3 "35-44" 4 "45-54" 5 "55-64" 6 "65+" 0 "Missing"
label values AGE_PART AGEPARTICIPANT
codebook AGE_PART

label variable AGE_PARTNER "Age_Partner"
label define AGEPARTNER 1 "18-24" 2 "25-34" 3 "35-44" 4 "45-54" 5 "55-64" 6 "65+" 7 "Don't_know" 0 "Missing"
label values AGE_PARTNER AGEPARTNER
codebook AGE_PARTNER

*Citizenship Status*

label variable CITIZEN_STATUS "Participant_citizenship_status"
label define CITIZENPARTICIPANT 1 "Canadian citizen" 2 "landed_imm_Perm_Res" 3 "Refugee_Protected_Person" 4 "Refugee claimnant" 5 "Temporary_worker" 6 "Int_Student" 7 "Don't_know" 0 "Missing"
label values CITIZEN_STATUS CITIZENPARTICIPANT
codebook CITIZEN_STATUS

label variable STATUS_PARTNER "Partner_citizenship_status"
label define CITIZENPARTNER 1 "Canadian citizen" 2 "landed_imm_Perm_Res" 3 "Refugee_Protected_Person" 4 "Refugee claimnant" 5 "Temporary_worker" 6 "Int_Student" 7 "Don't_know" 0 "Missing"
label values STATUS_PARTNER CITIZENPARTNER
```

```
codebook STATUS_PARTNER

*Race1*
label variable RACE_PARTICIPANT "Participant_Race"
label define RACEPARTICIPANT 1 "Racialized" 2 "Caucasian/White" 3 "Indigenous" 4 "PrefNotSay" 0 "Missing"
label values RACE_PARTICIPANT RACEPARTICIPANT
codebook RACE_PARTICIPANT

label variable RACE_PARTNER "Participant_Race"
label define RACEPARTNER 1 "Racialized" 2 "Caucasian/White" 3 "Indigenous" 4 "PrefNotSay" 0 "Missing"
label values RACE_PARTNER RACEPARTNER
codebook RACE_PARTNER

label variable RACE_PARTICIPANT_2 "Participant Race"
label define RACEPARTICIPANT2 1 "Black" 2 "Caucasian/White" 3 "East Asian" 4 "Indigenous" 5 "Latin American" 6 "Middle East" 7 "North African" 8 "Southeast Asian" 9 "South Asian" 10 "Don't Know" 11 "Prefer Not to Say" 13 "Mixed Race"
label values RACE_PARTICIPANT_2 RACEPARTICIPANT2
codebook RACE_PARTICIPANT_2

label variable RACE_PARTNER_2 "Partner Race OG"
label define RACE_PARTNER2 1 "Black" 2 "Caucasian/White" 3 "East Asian" 4 "Indigenous" 5 "Latin American" 6 "Middle East" 7 "South Asian" 8 "Southeast Asian" 9 "North African" 10 "Don't Know" 11 "Prefer Not to Say" 13 "Mixed Race"
label values RACE_PARTNER_2 RACE_PARTNER2
codebook RACE_PARTNER_2

*Education*

label variable EDUC_PART "Participant_Education"
label define EDUCPART 1 "NorCertificate" 2 "HS_Diploma" 3 "TradeCert/Diploma" 4 "College/CEGEP" 5 "UnivCert/Diploma" 6 "Bachelors" 7 "Masters" 8 "PHD" 0 "Missing"
label values EDUC_PART EDUCPART
codebook EDUC_PART

label variable EDUC_PARTNER "Partner_Education"
label define EDUCPARTNER 1 "NoCertificate" 2 "HS_Diploma" 3 "TradeCert/Diploma" 4 "College/CEGEP" 5 "UnivCert/Diploma" 6 "Bachelors" 7 "Masters" 8 "PHD" 9 "Don't_Know" 0 "Missing"
label values EDUC_PARTNER EDUCPARTNER
codebook EDUC_PARTNER

*Employment Status*
```

```
*During COVID*

label variable EMPL_PART_DURING "Participant_Employment_Status_during_COVID"
label define EMPPARTICIPANTCOV 1 "Yes" 2 "No" 3 "Retired" 0 "Missing"
label values EMPL_PART_DURING EMPPARTICIPANTCOV
codebook EMPL_PART_DURING

label variable EMP_PARTNER_DURING "Partner_Employment_Status_during_COVID"
label define EMPPARTNERCOV 1 "Yes" 2 "No" 3 "Retired" 4 "Uknown"
label values EMP_PARTNER_DURING EMPPARTNERCOV
codebook EMP_PARTNER_DURING

*Geography*

label variable GEOGRAPHY "Geography"
label define GEO 1 "EASTERNOnt" 2 "CENTRALOnt" 3 "TORONTO" 4 "GTA" 5 "WESTERNOnt" 6 "NORTHERNOnt" 0 "Missing"
label values GEOGRAPHY GEO
codebook GEOGRAPHY

label variable RURALURBAN "Rural or Urban"
label define RU_URB 1 "Rural" 2 "Urban" 0 "Missing"
label values RURALURBAN RU_URB
codebook RURALURBAN

*Household Income*

label variable HH_INCOME "Household_Income"
label define HH 1 "<30,000" 2 "30-39,999" 3 "40-49,999" 4 "50-69,999" 5 "70-99,999" 6 ">100,000" 7 "Don't_Know" 8 "PrefNotSay" 0 "Missing"
label values HH_INCOME HH
codebook HH_INCOME

*Owned Residence During COVID*

label variable OWN_RES_DURING "Own Residence During Pandemic"
label define OWNRES 1 "Yes, I co-own/co-owned my residence with my partner" 2 "Yes, I own/owned the residence_it is under my name only" 3 "No_Hous is/was under partnre's name only" 4 "No, neither own residence we live in" 5 "No_I live/lived with family" 0 "Missing"
label values OWN_RES_DURING OWNRES
codebook OWN_RES_DURING

label variable TYPE_RES_DURING "ResidenceType_During_Pandemic"
```

```
label variable TYPE_RES_DURING "ResidenceType_During_Pandemic"
label define TYPERES 1 "Single detached" 2 "Apartment_Lowrise" 3 "Apartment_highrise" 4 "TownHome" 5 "Semi-detached" 6"Shelter" 0 "Missing"
label values TYPE_RES_DURING TYPERES
codebook TYPE_RES_DURING

**Community Violence**

label variable COMM_VIOL_DURING "Community_Violence_Problem_During COVID"
label define COMVIOL 1 "No" 2 "Yes" 0 "Missing"
label values COMM_VIOL_DURING COMVIOL
codebook COMM_VIOL_DURING

*Partner Type*

label variable PARTNER_TYPE "PARTNER TYPE"
label define PARTTYPE 1 "Wife" 2 "Husband" 3 "ComLawWife" 4 "ComLawHus" 5 "Boyfriend" 6 "Girlfriend" 7 "PrefNotSay" 0 "Missing"
label values PARTNER_TYPE PARTTYPE
codebook PARTNER_TYPE

*length of relationship*

label variable LENGTH_COMBINED "Length of relationship"
label define LENGTH 1 "1-6 months" 2 "7-11 months" 3 "1-2 yrs" 4 ">2yrs" 0 "Missing"
label values LENGTH_COMBINED LENGTH
codebook LENGTH_COMBINED

*PARTNER DRINKING
label variable WISH_DRINK_COMBINED "Wish_did_not_DRINK"
label define DRINK 5 "Almost Always" 4 "Often" 3 "Sometimes" 2 "Seldom" 1 "Never" 0 "Missing"
label values WISH_DRINK_COMBINED DRINK
codebook WISH_DRINK_COMBINED

label variable STRAIN_DRINK_COMBINED "DrinkingSourceOfStrain"
label define STRAINALC 5 "Almost Always" 4 "Often" 3 "Sometimes" 2 "Seldom" 1 "Never" 0 "Missing"
label values STRAIN_DRINK_COMBINED STRAINALC
codebook STRAIN_DRINK_COMBINED

label variable CON_LEAVE_DRINK_COMBINED "ConsideredLeavingBCDRINK"
label define LEAVEALC 5 "Almost Always" 4 "Often" 3 "Sometimes" 2 "Seldom" 1 "Never" 0 "Missing"
label values CON_LEAVE_DRINK_COMBINED LEAVEALC
```

```
codebook CON_LEAVE_DRINK_COMBINED

label variable WISH_DRUGS_COMBINED "Wish_did_not_DRUGS"
label define DRUGS 5 "Almost Always" 4 "Often" 3 "Sometimes" 2 "Seldom" 1 "Never" 0 "Missing"
label values WISH_DRUGS_COMBINED DRUGS
codebook WISH_DRUGS_COMBINED

label variable STRAIN_DRUGS_COMBINED "DRUGSSourceOfStrain"
label define STRAINDRUGS 5 "Almost Always" 4 "Often" 3 "Sometimes" 2 "Seldom" 1 "Never" 0 "Missing"
label values STRAIN_DRUGS_COMBINED STRAINDRUGS
codebook STRAIN_DRUGS_COMBINED

label variable CON_LEAVE_DRUGS_COMBINED "ConsideredLeavingBCDRUGS"
label define LEAVEDRUGS 5 "Almost Always" 4 "Often" 3 "Sometimes" 2 "Seldom" 1 "Never" 0 "Missing"
label values CON_LEAVE_DRUGS_COMBINED LEAVEDRUGS
codebook CON_LEAVE_DRUGS_COMBINED

*EXPERIENCE OF IPV*
*BEFORE COVID*

label variable PHYS_B4 "Physically Hurt You B4 COVID"
label define PHYSB4COV 1 "Never" 2 "Once" 3 "A few times" 4 "monthly" 5 "weekly" 6 "almost dialy" 0 "Missing"
label values PHYS_B4 PHYSB4COV
codebook PHYS_B4

label variable INSULT_B4 "Insult or talk down to you_B4_COVID"
label define INSULTB4COV 1 "Never" 2 "Once" 3 "A few times" 4 "monthly" 5 "weekly" 6 "almost dialy" 0 "Missing"
label values INSULT_B4 INSULTB4COV
codebook INSULT_B4

label variable THREAT_HARM_B4 "Threaten with Harm B4 COVID"
label define HARMB4COVID 1 "Never" 2 "Once" 3 "A few times" 4 "monthly" 5 "weekly" 6 "almost dialy" 0 "Missing"
label values THREAT_HARM_B4 HARMB4COVID
codebook THREAT_HARM_B4

label variable SCREAM_CURSE_B4 "Scream_Curse_at You B4 COVID"
label define SCREAMB4COVID 1 "Never" 2 "Once" 3 "A few times" 4 "monthly" 5 "weekly" 6 "almost dialy" 0 "Missing"
label values SCREAM_CURSE_B4 SCREAMB4COVID
codebook SCREAM_CURSE_B4

label variable SEXUAL_B4 "Force You to have sexual activities B4
```

```
COVID"
label define SEXUALB4COVID 1 "Never" 2 "Once" 3 "A few times" 4 "monthly" 5 "weekly" 6 "almost dialy" 0 "Missing"
label values SEXUAL_B4 SEXUALB4COVID
codebook SEXUAL_B4

label variable EMP_MON_B4 "Keep you from access to job/employ_B4_COVID"
label define KEEPEMPMONB4COVID 1 "Never" 2 "Once" 3 "A few times" 4 "monthly" 5 "weekly" 6 "almost dialy" 0 "Missing"
label values EMP_MON_B4 KEEPEMPMONB4COVID
codebook EMP_MON_B4

label variable FAM_FRIEND_B4 "Keep_from seeing_FAM_FRNDS_B4_COVID"
label define KEEPSEEFAMFRNDB4COVID 1 "Never" 2 "Once" 3 "A few times" 4 "monthly" 5 "weekly" 6 "almost dialy" 0 "Missing"
label values FAM_FRIEND_B4 KEEPSEEFAMFRNDB4COVID
codebook FAM_FRIEND_B4

label variable TECH_DIGIT_B4 "Tech/Digital_Violence_B4_COVID"
label define TECHVIOLB4COVID 1 "Never" 2 "Once" 3 "A few times" 4 "monthly" 5 "weekly" 6 "almost dialy" 0 "Missing"
label values TECH_DIGIT_B4 TECHVIOLB4COVID
codebook TECH_DIGIT_B4

label variable REL_SPIRT_B4 "Religious_Spirit_VIOL_B4_COVID"
label define RELIGVIOLB4COVID 1 "Never" 2 "Once" 3 "A few times" 4 "monthly" 5 "weekly" 6 "almost dialy" 0 "Missing"
label values REL_SPIRT_B4 RELIGVIOLB4COVID
codebook REL_SPIRT_B4

*DURING COVID*

label variable PHYS_DURING "Physically Hurt You DURING COVID"
label define PHYSinCOV 1 "Did not happen" 2 "MuchBetter" 3 "SomewhatBetter" 4 "StayedtheSAME" 5 "SOmwhatWorse" 6 "MuchWorse"
label values PHYS_DURING PHYSinCOV
codebook PHYS_DURING

label variable INSULT_DURING "Insult or talk down to you_DURING_COVID"
label define INSULTinCOV 1 "Did not happen" 2 "MuchBetter" 3 "SomewhatBetter" 4 "StayedtheSAME" 5 "SOmwhatWorse" 6 "MuchWorse"
label values INSULT_DURING INSULTinCOV
codebook INSULT_DURING

label variable THREAT_HARM_DURING "Threaten with Harm DURING COVID"
label define HARMinCOVID 1 "Did not happen" 2 "MuchBetter" 3 "SomewhatBetter" 4 "StayedtheSAME" 5 "SOmwhatWorse" 6 "MuchWorse"
```

```
"SomewhatBetter" 4 "StayedtheSAME" 5 "SOmwhatWorse" 6 "MuchWorse"
label values THREAT_HARM_DURING HARMinCOVID
codebook THREAT_HARM_DURING

label variable SCREAM_CURSE_DURING "Scream_Curse_at You DURING COVID"
label define SCREAMinCOVID 1 "Did not happen" 2 "MuchBetter" 3
"SomewhatBetter" 4 "StayedtheSAME" 5 "SOmwhatWorse" 6 "MuchWorse"
label values SCREAM_CURSE_DURING SCREAMinCOVID
codebook SCREAM_CURSE_DURING

label variable SEXUAL_DURING "Force You to have sexual activities
DURING COVID"
label define SEXUALinCOVID 1 "Did not happen" 2 "MuchBetter" 3
"SomewhatBetter" 4 "StayedtheSAME" 5 "SOmwhatWorse" 6 "MuchWorse"
label values SEXUAL_DURING SEXUALinCOVID
codebook SEXUAL_DURING

label variable EMP_MON_DURING "Keep you from access to
job/employ_DURING_COVID"
label define KEEPEMPMONinCOVID 1 "Did not happen" 2 "MuchBetter" 3
"SomewhatBetter" 4 "StayedtheSAME" 5 "SOmwhatWorse" 6 "MuchWorse"
label values EMP_MON_DURING KEEPEMPMONinCOVID
codebook EMP_MON_DURING

label variable FAM_FRIEND_DURING "Keep_from
seeing_FAM_FRNDS_DURING_COVID"
label define KEEPSEEFAMFRNDinCOVID 1 "Did not happen" 2 "MuchBetter" 3
 "SomewhatBetter" 4 "StayedtheSAME" 5 "SOmwhatWorse" 6 "MuchWorse"
label values FAM_FRIEND_DURING KEEPSEEFAMFRNDinCOVID
codebook FAM_FRIEND_DURING

label variable TECH_DIGIT_DURING "Tech/Digital_Violence_DURING_COVID"
label define TECHVIOLinCOVID 1 "Did not happen" 2 "MuchBetter" 3
"SomewhatBetter" 4 "StayedtheSAME" 5 "SOmwhatWorse" 6 "MuchWorse"
label values TECH_DIGIT_DURING TECHVIOLinCOVID
codebook TECH_DIGIT_DURING

label variable RELI_SPRIT_DURING "Religious_Spirit_VIOL_DURING_COVID"
label define RELIGVIOLinCOVID 1 "Did not happen" 2 "MuchBetter" 3
"SomewhatBetter" 4 "StayedtheSAME" 5 "SOmwhatWorse" 6 "MuchWorse"
label values RELI_SPRIT_DURING RELIGVIOLinCOVID
codebook RELI_SPRIT_DURING

*POST_1 COVID*

label variable PHYS_POST_1 "Physically Hurt You POST COVID"
```

```
label variable PHYS_POST_1 "Physically Hurt You POST COVID"
label define PHYSPOSTCOV 1 "Did not happen" 2 "MuchBetter" 3 "SomewhatBetter" 4 "StayedtheSAME" 5 "SOmwhatWorse" 6 "MuchWorse"
label values PHYS_POST_1 PHYSPOSTCOV
codebook PHYS_POST_1

label variable INSULT_POST_1 "Insult or talk down to you_POST_COVID"
label define INSULTPOSTCOV 1 "Did not happen" 2 "MuchBetter" 3 "SomewhatBetter" 4 "StayedtheSAME" 5 "SOmwhatWorse" 6 "MuchWorse"
label values INSULT_POST_1 INSULTPOSTCOV
codebook INSULT_POST_1

label variable THREAT_HARM_POST_1 "Threaten with Harm POST COVID"
label define THREAT_HARM_POST_BOTH 1 "Did not happen" 2 "MuchBetter" 3 "SomewhatBetter" 4 "StayedtheSAME" 5 "SOmwhatWorse" 6 "MuchWorse"
label values THREAT_HARM_POST_1 HARMPOSTCOVID
codebook THREAT_HARM_POST_1

label variable SCREAM_CURSE_POST_1 "Scream_Curse_at You POST COVID"
label define SCREAMPOSTCOVID 1 "Did not happen" 2 "MuchBetter" 3 "SomewhatBetter" 4 "StayedtheSAME" 5 "SOmwhatWorse" 6 "MuchWorse"
label values SCREAM_CURSE_POST_1 SCREAMPOSTCOVID
codebook SCREAM_CURSE_POST_1

label variable SEXUAL_POST_1 "Force You to have sexual activities POST COVID"
label define SEXUALPOSTCOVID 1 "Did not happen" 2 "MuchBetter" 3 "SomewhatBetter" 4 "StayedtheSAME" 5 "SOmwhatWorse" 6 "MuchWorse"
label values SEXUAL_POST_1 SEXUALPOSTCOVID
codebook SEXUAL_POST_1

label variable EMP_MON_POST_1 "Keep you frmo access to job/employ_POST_COVID"
label define KEEPEMPMONPOSTCOVID 1 "Did not happen" 2 "MuchBetter" 3 "SomewhatBetter" 4 "StayedtheSAME" 5 "SOmwhatWorse" 6 "MuchWorse"
label values EMP_MON_POST_1 KEEPEMPMONPOSTCOVID
codebook EMP_MON_POST_1

label variable FAM_FRIEND_POST_1 "Keep_from seeing_FAM_FRNDS_POST_COVID"
label define KEEPSEEFAMFRNDPOSTCOVID 1 "Did not happen" 2 "MuchBetter" 3 "SomewhatBetter" 4 "StayedtheSAME" 5 "SOmwhatWorse" 6 "MuchWorse"
label values FAM_FRIEND_POST_1 KEEPSEEFAMFRNDPOSTCOVID
codebook FAM_FRIEND_POST_1

label variable TECH_DIGIT_POST_1 "Tech/Digital_Violence_POST_COVID"
label define TECHVIOLPOSTCOVID 1 "Did not happen" 2 "MuchBetter" 3 "SomewhatBetter" 4 "StayedtheSAME" 5 "SOmwhatWorse" 6 "MuchWorse"
```

```
"SomewhatBetter" 4 "StayedtheSAME" 5 "SOmwhatWorse" 6 "MuchWorse"
label values TECH_DIGIT_POST_1 TECHVIOLPOSTCOVID
codebook TECH_DIGIT_POST_1

label variable REL_SPIRT_POST_1 "Religious_Spirit_VIOL_POST_COVID"
label define RELIGVIOLPOSTCOVID 1 "Did not happen" 2 "MuchBetter" 3 "SomewhatBetter" 4 "StayedtheSAME" 5 "SOmwhatWorse" 6 "MuchWorse"
label values REL_SPIRT_POST_1 RELIGVIOPOSTCOVID
codebook REL_SPIRT_POST_1


*POST_2 COVID*

label variable PHYS_POST_2 "Physically Hurt You POST COVID"
label define PHYSPOSTCOV2 1 "Did not happen" 2 "MuchBetter" 3 "SomewhatBetter" 4 "StayedtheSAME" 5 "SOmwhatWorse" 6 "MuchWorse"
label values PHYS_POST_2 PHYSPOSTCOV
codebook PHYS_POST_2

label variable INSULT_POST_2 "Insult or talk down to you_POST_COVID"
label define INSULTPOSTCOV2 1 "Did not happen" 2 "MuchBetter" 3 "SomewhatBetter" 4 "StayedtheSAME" 5 "SOmwhatWorse" 6 "MuchWorse"
label values INSULT_POST_2 INSULTPOSTCOV
codebook INSULT_POST_2

label variable THREAT_HARM_POST_2 "Threaten with Harm POST COVID"
label define THREAT_HARM_POSTCOV2 1 "Did not happen" 2 "MuchBetter" 3 "SomewhatBetter" 4 "StayedtheSAME" 5 "SOmwhatWorse" 6 "MuchWorse"
label values THREAT_HARM_POST_2 HARMPOSTCOVID
codebook THREAT_HARM_POST_2

label variable SCREAM_CURSE_POST_2 "Scream_Curse_at You POST COVID"
label define SCREAMPOSTCOVID2 1 "Did not happen" 2 "MuchBetter" 3 "SomewhatBetter" 4 "StayedtheSAME" 5 "SOmwhatWorse" 6 "MuchWorse"
label values SCREAM_CURSE_POST_2 SCREAMPOSTCOVID
codebook SCREAM_CURSE_POST_2

label variable SEXUAL_POST_2 "Force You to have sexual activities POST COVID"
label define SEXUALPOSTCOVID2 1 "Did not happen" 2 "MuchBetter" 3 "SomewhatBetter" 4 "StayedtheSAME" 5 "SOmwhatWorse" 6 "MuchWorse"
label values SEXUAL_POST_2 SEXUALPOSTCOVID
codebook SEXUAL_POST_2

label variable EMP_MON_POST_2 "Keep you frmo access to job/employ_POST_COVID"
label define KEEPEMPMONPOSTCOVID2 1 "Did not happen" 2 "MuchBetter" 3 "SomewhatBetter" 4 "StayedtheSAME" 5 "SOmwhatWorse" 6 "MuchWorse"
```

```
label values EMP_MON_POST_2 KEEPEMPMONPOSTCOVID
codebook EMP_MON_POST_2

label variable FAM_FRIEND_POST_2 "Keep_from seeing_FAM_FRNDS_POST_COVID"
label define KEEPSEEFAMFRNDPOSTCOVID2 1 "Did not happen" 2 "MuchBetter" 3 "SomewhatBetter" 4 "StayedtheSAME" 5 "SOmwhatWorse" 6 "MuchWorse"
label values FAM_FRIEND_POST_2 KEEPSEEFAMFRNDPOSTCOVID
codebook FAM_FRIEND_POST_2

label variable TECH_DIGIT_POST_2 "Tech/Digital_Violence_POST_COVID"
label define TECHVIOLPOSTCOVID2 1 "Did not happen" 2 "MuchBetter" 3 "SomewhatBetter" 4 "StayedtheSAME" 5 "SOmwhatWorse" 6 "MuchWorse"
label values TECH_DIGIT_POST_2 TECHVIOLPOSTCOVID
codebook TECH_DIGIT_POST_2

label variable REL_SPIRT_POST_2 "Religious_Spirit_VIOL_POST_COVID"
label define RELIGVIOLPOSTCOVID2 1 "Did not happen" 2 "MuchBetter" 3 "SomewhatBetter" 4 "StayedtheSAME" 5 "SOmwhatWorse" 6 "MuchWorse"
label values REL_SPIRT_POST_2 RELIGVIOPOSTCOVID
codebook REL_SPIRT_POST_2


***DROPPING CASES****

drop if DROP==1
tab DROP, m

*********************************
*   RECODING VARIABLES  *
*********************************

********************************************************************************
*INDEPENDANT_EXPLANATORY VARIABLES: Q1. EXPERIENCE OF IPV DURING COVID
********************************************************************************

recode IPV_EXP (1=1 "Yes IPV") (2=0 "No IPV") (.=.), gen (dumYES_IPV)
tab IPV_EXP dumYES_IPV, m

recode IPV_EXP (2=1 "No IPV") (1=0 "Yes IPV") (.=.), gen (dumNo_IPV)
tab IPV_EXP dumNo_IPV, m

********************************************************************************
```

```
********************************************************************************
*OUTCOME VARIABLE #1 - Perceived Health Outcomes
********************************************************************************

**CREATING A BINARY VARIABLE**

*MENTAL HEALTH*

* Step 1: Generate a new binary variable for mental health outcomes
gen MENTAL_HEALTH_OUTCOME = .

* Step 2: Assign "Bad Mental Health" (1) to those who:
* - Said "About the Same" during COVID-19 but were "Poor" before COVID-19
* - Said "Worse" or "Much Worse" during COVID-19
replace MENTAL_HEALTH_OUTCOME = 1 if MENTAL_IN_COVID == 3 & MENTAL_B4_COVID == 5
replace MENTAL_HEALTH_OUTCOME = 1 if inlist(MENTAL_IN_COVID, 4, 5)

* Step 3: Assign "Good Mental Health" (0) to those who:
* - Said "About the Same" during COVID-19 but had better mental health (Excellent, Very Good, Good, or Fair) before COVID-19
* - Said "Better" or "Much Better" during COVID-19
replace MENTAL_HEALTH_OUTCOME = 0 if MENTAL_IN_COVID == 3 & inlist(MENTAL_B4_COVID, 1, 2, 3, 4)
replace MENTAL_HEALTH_OUTCOME = 0 if inlist(MENTAL_IN_COVID, 1, 2)

* Step 4: Assign "Missing" to other cases (e.g., missing values in the original variables)
replace MENTAL_HEALTH_OUTCOME = . if MENTAL_IN_COVID == 0 | MENTAL_B4_COVID == 0

* Step 5: Label the new variable and values for clarity
label variable MENTAL_HEALTH_OUTCOME "Mental Health Outcome During COVID-19"
label define MENTOUTCOME 0 "Good Mental Health" 1 "Bad Mental Health"
label values MENTAL_HEALTH_OUTCOME MENTOUTCOME

*PHYSICAL HEALTH*
* Step 1: Generate a new binary variable for physical health outcomes
gen PHYSICAL_HEALTH_OUTCOME = .

* Step 2: Assign "Bad Physical Health" (1) to those who:
* - Said "About the Same" during COVID-19 but were "Poor" before COVID-19
* - Said "Worse" or "Much Worse" during COVID-19
```

```
* - Said "Worse" or "Much Worse" during COVID-19
replace PHYSICAL_HEALTH_OUTCOME = 1 if PHYS_IN_COVID == 3 & PHYS_B4_COVID == 5
replace PHYSICAL_HEALTH_OUTCOME = 1 if inlist(PHYS_IN_COVID, 4, 5)

* Step 3: Assign "Good Physical Health" (0) to those who:
* - Said "About the Same" during COVID-19 but had better physical health (Excellent, Very Good, Good, or Fair) before COVID-19
* - Said "Better" or "Much Better" during COVID-19
replace PHYSICAL_HEALTH_OUTCOME = 0 if PHYS_IN_COVID == 3 & inlist(PHYS_B4_COVID, 1, 2, 3, 4)
replace PHYSICAL_HEALTH_OUTCOME = 0 if inlist(PHYS_IN_COVID, 1, 2)

* Step 4: Assign "Missing" to other cases (e.g., missing values in the original variables)
replace PHYSICAL_HEALTH_OUTCOME = . if PHYS_IN_COVID == 0 | PHYS_B4_COVID == 0

* Step 5: Label the new variable and values for clarity
label variable PHYSICAL_HEALTH_OUTCOME "Physical Health Outcome During COVID-19"
label define PHYSOUTCOME 0 "Good Physical Health" 1 "Bad Physical Health"
label values PHYSICAL_HEALTH_OUTCOME PHYSOUTCOME

recode MENTAL_HEALTH_OUTCOME (1 = 1 "BadMental") (0=0 "GoodMental"), gen (BadMentHealth)
tab MENTAL_HEALTH_OUTCOME BadMentHealth, m

recode MENTAL_HEALTH_OUTCOME (1 = 0 "BadMental") (0=1 "GoodMental"), gen (GoodMentHealth)
tab MENTAL_HEALTH_OUTCOME GoodMentHealth, m

recode PHYSICAL_HEALTH_OUTCOME (1 = 1 "BadPhys") (0=0 "GoodPhys"), gen (BadPhysHealth)
tab PHYSICAL_HEALTH_OUTCOME BadPhysHealth, m

recode PHYSICAL_HEALTH_OUTCOME (1 = 0 "BadPhys") (0=1 "GoodPhys"), gen (GoodPhysHealth)
tab PHYSICAL_HEALTH_OUTCOME GoodPhysHealth, m

************************************************************
**HEALTH BEHAVIOURS**
************************************************************

***CHANGE IN BEHVIOURS***

recode ALCOHOL_DURING (1=1 "increased") (2 3 =0 "Other") (0=.) (.=.),
```

```
recode ALCOHOL_DURING (1=1 "increased") (2 3 =0 "Other") (0=.) (.=.), gen (AlcoholIncreased)
tab ALCOHOL_DURING AlcoholIncreased, m

recode ALCOHOL_DURING (2=1 "decreased") (1 3 =0 "Other") (0=.) (.=.), gen (AlcoholDecreased)
tab ALCOHOL_DURING AlcoholDecreased, m

recode ALCOHOL_DURING (3=1 "No Change") (1 2 =0 "Other") (0=.) (.=.), gen (AlcoholNoChange)
tab ALCOHOL_DURING AlcoholNoChange, m

recode TOBAC_DRUING (1=1 "increased") (2 3 =0 "Other") (0=.) (.=.), gen (TOBACIncreased)
tab TOBAC_DRUING TOBACIncreased, m

recode TOBAC_DRUING (2=1 "decreased") (1 3 =0 "Other") (0=.) (.=.), gen (TOBACDecreased)
tab TOBAC_DRUING TOBACDecreased, m

recode TOBAC_DRUING (3=1 "No Change") (1 2 =0 "Other") (0=.) (.=.), gen (TOBACNoChange)
tab TOBAC_DRUING TOBACNoChange, m

recode CANNAB_DURING (1=1 "increased") (2 3 =0 "Other") (0=.) (.=.), gen (CANNABIncreased)
tab CANNAB_DURING CANNABIncreased, m

recode CANNAB_DURING (2=1 "decreased") (1 3 =0 "Other") (0=.) (.=.), gen (CANNABDecreased)
tab CANNAB_DURING CANNABDecreased, m

recode CANNAB_DURING (3=1 "No Change") (1 2 =0 "Other") (0=.) (.=.), gen (CANNABNoChange)
tab CANNAB_DURING CANNABNoChange, m

recode SUBSTANCE_DURING (1=1 "increased") (2 3 =0 "Other") (0=.) (.=.), gen (SUBSTANCEIncreased)
tab SUBSTANCE_DURING SUBSTANCEIncreased, m

recode SUBSTANCE_DURING (2=1 "decreased") (1 3 =0 "Other") (0=.) (.=.), gen (SUBSTANCEDecreased)
tab SUBSTANCE_DURING SUBSTANCEDecreased, m

recode SUBSTANCE_DURING (3=1 "No Change") (1 2 =0 "Other") (0=.) (.=.), gen (SUBSTANCENoChange)
tab SUBSTANCE_DURING SUBSTANCENoChange, m
```

```
recode TV_DURING (1=1 "increased") (2 3 =0 "Other") (0=.) (.=.), gen (TV_Increased)
tab TV_DURING TV_Increased, m

recode TV_DURING (2=1 "decreased") (1 3 =0 "Other") (0=.) (.=.), gen (TV_Decreased)
tab TV_DURING TV_Decreased, m

recode TV_DURING (3=1 "No Change") (1 2 =0 "Other") (0=.) (.=.), gen (TV_NoChange)
tab TV_DURING TV_NoChange, m

recode INTERNET_DURING (1=1 "increased") (2 3 =0 "Other") (0=.) (.=.), gen (INTERNET_Increased)
tab INTERNET_DURING INTERNET_Increased, m

recode INTERNET_DURING (2=1 "decreased") (1 3 =0 "Other") (0=.) (.=.), gen (INTERNET_Decreased)
tab INTERNET_DURING INTERNET_Decreased, m

recode INTERNET_DURING (3=1 "No Change") (1 2 =0 "Other") (0=.) (.=.), gen (INTERNET_NoChange)
tab INTERNET_DURING INTERNET_NoChange, m

recode EXERCISING_DURING (1=1 "increased") (2 3 =0 "Other") (0=.) (.=.), gen (EXERCISE_Increased)
tab EXERCISING_DURING EXERCISE_Increased, m

recode EXERCISING_DURING (2=1 "decreased") (1 3 =0 "Other") (0=.) (.=.), gen (EXECRISE_Decreased)
tab EXERCISING_DURING EXECRISE_Decreased, m

recode EXERCISING_DURING (3=1 "No Change") (1 2 =0 "Other") (0=.) (.=.), gen (EXERCISE_NoChange)
tab EXERCISING_DURING EXERCISE_NoChange, m

recode EATJUNK_DURING (1=1 "increased") (2 3 =0 "Other") (0=.) (.=.), gen (JUNK_Increased)
tab EATJUNK_DURING JUNK_Increased, m

recode EATJUNK_DURING (2=1 "decreased") (1 3 =0 "Other") (0=.) (.=.), gen (JUNK_Decreased)
tab EATJUNK_DURING JUNK_Decreased, m

recode EATJUNK_DURING (3=1 "No Change") (1 2 =0 "Other") (0=.) (.=.), gen (JUNK_NoChange)
tab EATJUNK_DURING JUNK_NoChange, m
```

```
*COVARIATES***
************************************************************

**Change in seeking informal supports during COVID19*

recode INFORMAL_DURING (1=1 "increased") (2 3 =0 "Other") (0=.) (.=.), gen (SeekingINFIncreased)
tab INFORMAL_DURING SeekingINFIncreased, m

recode INFORMAL_DURING (2=1 "decreased") (1 3 =0 "Other") (0=.) (.=.), gen (SeekingINFdecreased)
tab INFORMAL_DURING SeekingINFdecreased, m

recode INFORMAL_DURING (3 =1 "No Change") (1 2 =0 "Other") (0=.) (.=.), gen (NoChangeInSeekingINF)
tab INFORMAL_DURING NoChangeInSeekingINF, m

***Informal Seeking2***
recode INFORMAL_DURING (1 3=1 "increased/nochange") (2=0 "decreased") (0=.) (.=.), gen (SeekingINFIncreased_NC2)
tab INFORMAL_DURING SeekingINFIncreased_NC2, m

recode INFORMAL_DURING (2=1 "decreased") (1 3 =0 "increased/nochange") (0=.) (.=.), gen (SeekingINFdecreased2)
tab INFORMAL_DURING SeekingINFdecreased2, m

**Change in seeking formal supports during COVID19*

recode FORMAL_DURING (1=1 "increased") (2 3 =0 "Other") (0=.) (.=.), gen (SeekingFORMIncreased)
tab FORMAL_DURING SeekingFORMIncreased, m

recode FORMAL_DURING (2=1 "decreased") (1 3 =0 "Other") (0=.) (.=.), gen (SeekingFORMdecreased)
tab FORMAL_DURING SeekingFORMdecreased, m

recode FORMAL_DURING (3 =1 "No Change") (1 2 =0 "Other") (0=.) (.=.), gen (NoChangeInSeekingFORM)
tab FORMAL_DURING NoChangeInSeekingFORM, m

**Formal seeking 2**

recode FORMAL_DURING (1 3=1 "increased/nochange") (2=0 "decreased") (0=.) (.=.), gen (SeekingFORMIncreased_NC2)
tab FORMAL_DURING SeekingFORMIncreased_NC2, m

recode FORMAL_DURING (2=1 "decreased") (1 3 =0 "increased/nochange") (0=.) (.=.), gen (SeekingFORMdecreased2)
```

```
tab FORMAL_DURING SeekingFORMdecreased2, m

****Change in communicating with Friends during COVID19*

recode COMM_FRND_DURING (1=1 "increased") (2 3 =0 "Other") (0=.) (.=.), gen (CommFRNDSIncreased)
tab COMM_FRND_DURING CommFRNDSIncreased, m

recode COMM_FRND_DURING (2=1 "decreased") (1 3 =0 "Other") (0=.) (.=.), gen (CommFRNDSdecreased)
tab COMM_FRND_DURING CommFRNDSdecreased, m

recode COMM_FRND_DURING (3 =1 "No Change") (1 2 =0 "Other") (0=.) (.=.), gen (NoChangeCommFRNDS)
tab COMM_FRND_DURING NoChangeCommFRNDS, m

***Communicating with friends 2*

recode COMM_FRND_DURING (1 3=1 "increased/nochange") (2=0 "decreased") (0=.) (.=.), gen (CommFRNDSIncreased_NC2)
tab COMM_FRND_DURING CommFRNDSIncreased_NC2, m

recode COMM_FRND_DURING (2=1 "decreased") (1 3 =0 "increased/nochange") (0=.) (.=.), gen (CommFRNDSdecreased2)
tab COMM_FRND_DURING CommFRNDSdecreased2, m

****Change in communicating with family during COVID19*

recode COMM_FAM_DURING (1=1 "increased") (2 3 =0 "Other") (0=.) (.=.), gen (CommFAMILYSIncreased)
tab COMM_FAM_DURING CommFAMILYSIncreased, m

recode COMM_FAM_DURING (2=1 "decreased") (1 3 =0 "Other") (0=.) (.=.), gen (CommFAMILYdecreased)
tab COMM_FAM_DURING CommFAMILYdecreased, m

recode COMM_FAM_DURING (3 =1 "No Change") (1 2 =0 "Other") (0=.) (.=.), gen (NoChangeFAMILYCommFRNDS)
tab COMM_FAM_DURING NoChangeFAMILYCommFRNDS, m

***Communicating with family 2*

recode COMM_FAM_DURING (1 3=1 "increased/nochange") (2=0 "decreased") (0=.) (.=.), gen (CommFAMILYSIncreased_NC2)
tab COMM_FAM_DURING CommFAMILYSIncreased_NC2, m

recode COMM_FAM_DURING (2=1 "decreased") (1 3 =0 "increased/nochange") (0=.) (.=.), gen (CommFAMILYdecreased2)
```

```
tab COMM_FAM_DURING CommFAMILYdecreased2, m

***Composite - communication with friends and/or family decreased***

* Create composite variable for decreased communication with friends or family
gen DecreasedCommunication = 0 // Initialize variable with 0
replace DecreasedCommunication = 1 if CommFRNDSdecreased == 1 | CommFAMILYdecreased == 1

* Label the new variable for clarity
label variable DecreasedCommunication "Decreased communication with friends or family during COVID-19"

* Check the distribution of the new composite variable
tab DecreasedCommunication
codebook DecreasedCommunication

recode DecreasedCommunication (1=1 "Decreased") (0=0 "other")(.=.), gen (dumDecreasedComm2)
tab DecreasedCommunication dumDecreasedComm2, m

recode DecreasedCommunication (1=0 "Other") (0=1 "Decreased")(.=.), gen (dumNOTDecreasedComm2)
tab DecreasedCommunication dumNOTDecreasedComm2, m

**Quality of sleep**

recode SLEEP_DURING (1=1 "increased") (2 3 =0 "Other") (0=.) (.=.), gen (QualitySLEEPIncreased)
tab SLEEP_DURING QualitySLEEPIncreased, m

recode SLEEP_DURING (2=1 "decreased") (1 3 =0 "Other") (0=.) (.=.), gen (QualitySLEEPdecreased)
tab SLEEP_DURING QualitySLEEPdecreased, m

recode SLEEP_DURING (3 =1 "No Change") (1 2 =0 "Other") (0=.) (.=.), gen (NoChangeInSLEEP)
tab SLEEP_DURING NoChangeInSLEEP, m

recode SLEEP_DURING (2=1 "Decrease") (1 3 =0 "Increase/NoChange") (0 =.) (.=.), gen (dumSLEEP_DECREASEinCOV1)
tab SLEEP_DURING dumSLEEP_DECREASEinCOV1, m

recode SLEEP_DURING (1 3=1 "Increased/NoChange") (2=0 "Decreased") (0 =.) (.=.), gen (dumSLEEP_INCREASE_noCHangeinCOV)
tab SLEEP_DURING dumSLEEP_INCREASE_noCHangeinCOV, m
```

```
*****************************************************************************************
**CHARACTERISTICS**
*****************************************************************************************

*PARTNER TYPE

recode PARTNER_TYPE (1 3 6 =1 "Wife/GF/CLWif") (2 4 5 7 =0 "Other") (0=.) (.=.), gen (dumFEMALEPartner)
tab PARTNER_TYPE dumFEMALEPartner, m

recode PARTNER_TYPE (2 4 5 =1 "Husband/BF/CLHud") (1 3 6 7 =0 "Other") (0=.) (.=.), gen (dumMALEPartner)
tab PARTNER_TYPE dumMALEPartner, m

recode PARTNER_TYPE (7 =1 "PrefNotSay") (1/6 =0 "Other") (0=.) (.=.), gen (dumUnknownPartner)
tab PARTNER_TYPE dumUnknownPartner, m

tab PARTNER_TYPE GENDER_PARTNER, m

**AGE****

*Participant*

recode AGE_PART (1 2=1 "18-34") (3/6=0 "Other")(0=.) (.=.), gen (dumAgePARTIC_18_34)
tab AGE_PART dumAgePARTIC_18_34, m

recode AGE_PART (3 4=1 "35-54") (1 2 5 6=0 "Other")(0=.) (.=.), gen (dumAgePARTIC_35_54)
tab AGE_PART dumAgePARTIC_35_54, m

recode AGE_PART (5 6=1 "55+") (1 2 3 4 =0 "Other")(0=.) (.=.), gen (dumAgePARTIC_55Plus)
tab AGE_PART dumAgePARTIC_55Plus, m

*Partner*

recode AGE_PARTNER (1 2=1 "18-34") (3/6=0 "Other")(7=.)(0=.)(.=.), gen (dumAgePARTNER_18_34)
tab AGE_PARTNER dumAgePARTNER_18_34, m

recode AGE_PARTNER (3 4=1 "35-54") (1 2 5 6=0 "Other")(7=.)(0=.) (.=.), gen (dumAgePARTNER_35_54)
tab AGE_PARTNER dumAgePARTNER_35_54, m
```

```
recode AGE_PARTNER (5 6=1 "55+") (1 2 3 4=0 "Other")(7=.)(0=.) (.=.), gen (dumAgePARTNER_55Plus)
tab AGE_PARTNER dumAgePARTNER_55Plus, m

**RACE***

*Participant race shortened*

recode RACE_PARTICIPANT_2 (1 3 4 5 6 7 8 9 13 = 1 "Racialized/Indigenous") (2 10 11 = 0 "other")(0=.) (.=.), gen ( dumRacializedPARTIC)
tab RACE_PARTICIPANT_2 dumRacializedPARTIC, m

recode RACE_PARTICIPANT_2 (2=1 "White/Caucasian") (1 3 4 5 6 7 8 9 10 11 13 =0 "Other") (0=.) (.=.), gen (dumWHITEPARTIC)
tab RACE_PARTICIPANT_2 dumWHITEPARTIC, m

recode RACE_PARTICIPANT_2 (10 11 =1 "PrefNotSay") (1 2 3 4 5 6 7 8 9 13=0 "Other") (0=.) (.=.), gen (dumPrefNotSayRACEPARTIC)
tab RACE_PARTICIPANT_2 dumPrefNotSayRACEPARTIC, m

**Race partner shortened*

recode RACE_PARTNER_2 (1 3 4 5 6 7 8 9 13 =1 "Racialized/Indigenous") (2 10 11 =0 "Other") (0=.) (.=.), gen (dumRacializedPARTNER)
tab RACE_PARTNER_2 dumRacializedPARTNER, m

recode RACE_PARTNER_2 (2=1 "White/Caucasian") (1 3 4 5 6 7 8 9 10 11 13 =0 "Other") (0=.) (.=.), gen (dumWHITEPARTNER)
tab RACE_PARTNER_2 dumWHITEPARTNER, m

recode RACE_PARTNER_2 (10 11 =1 "PrefNotSay") (1 2 3 4 5 6 7 8 9 13=0 "Other") (0=.) (.=.), gen (dumPrefNotSayRACEPARNER)
tab RACE_PARTNER_2 dumPrefNotSayRACEPARNER, m

*Participant race shortened - combine racialized/indigenous/prefer not to say*

recode RACE_PARTICIPANT_2 (1 3 4 5 6 7 8 9 10 11 13 = 1 "Racialized/Indigenous/Pref_not") (2 = 0 "White/Caucasian")(0=.) (.=.), gen (dumRacializedPARTIC2)
tab RACE_PARTICIPANT_2 dumRacializedPARTIC2, m

recode RACE_PARTICIPANT_2 (2=1 "White/Caucasian") (1 3 4 5 6 7 8 9 10 11 13 =0 "Racialized/Indigenous/Pref_not") (0=.) (.=.), gen ( dumWHITEPARTIC2)
tab RACE_PARTICIPANT_2 dumWHITEPARTIC2, m
```

```
**Race partner shortened* - combine racialized/indigenous/prefer not to say*

recode RACE_PARTNER_2 (1 3 4 5 6 7 8 9 10 11 13 =1 "Racialized/Indigenous/Pref_not") (2=0 "White") (0=.) (.=.), gen (dumRacializedPARTNER2)
tab RACE_PARTNER_2 dumRacializedPARTNER2, m

recode RACE_PARTNER_2 (2=1 "White/Caucasian") (1 3 4 5 6 7 8 9 10 11 13 =0 "Racialized/Indigenous/Pref_not") (0=.) (.=.), gen (dumWHITEPARTNER2)
tab RACE_PARTNER_2 dumWHITEPARTNER2, m

***CITIZENSHIP STATUS****

*Participant*

*CITIZEN_STATUS
recode CITIZEN_STATUS (1=1 "Canadian Citizen") (2/7=0 "Other")(0=.) (.=.), gen (dumCANADIAN_PARTIC2)
tab CITIZEN_STATUS dumCANADIAN_PARTIC2, m

recode CITIZEN_STATUS (2 3 4 5 6 =1 "Immigrant_PR_Refugee") (1 7=0 "Other")(0=.) (.=.), gen (dumIMMIG_PR_REF_PARTIC2)
tab CITIZEN_STATUS dumIMMIG_PR_REF_PARTIC2, m

recode CITIZEN_STATUS (7=1 "unknown") (1/6=0 "Other")(0=.) (.=.), gen (dumCITIZEN_Uknown_PARTIC2)
tab CITIZEN_STATUS dumCITIZEN_Uknown_PARTIC2, m

*Citizenship Status 3

recode CITIZEN_STATUS (1=1 "Canadian Citizen") (2/6=0 "Other")(7=.)(0=.) (.=.), gen (dumCANADIAN_PARTIC3)
tab CITIZEN_STATUS dumCANADIAN_PARTIC3, m

recode CITIZEN_STATUS (2 3 4 5 6 =1 "Immigrant_PR_Refugee") (7=.) (1 = 0 "Other")(0=.) (.=.), gen (dumIMMIG_PR_REF_PARTIC3)
tab CITIZEN_STATUS dumIMMIG_PR_REF_PARTIC3, m


*PARTNER STATUS

recode STATUS_PARTNER (1=1 "Canadian Citizen") (2/7=0 "Other")(0=.) (.=.), gen (dumCANADIAN_PARTNER2)
tab STATUS_PARTNER dumCANADIAN_PARTNER2, m

recode STATUS_PARTNER (2 3 4 5 6 =1 "Immigrant_PR_Refugee") (1 7=0
```

```
"Other")(0=.) (.=.), gen (dumIMMIG_PR_REF_PARTNER2)
tab STATUS_PARTNER dumIMMIG_PR_REF_PARTNER2, m

recode STATUS_PARTNER (7=1 "unknown") (1/6=0 "Other")(0=.)(.=.), gen (
dumCITIZEN_Uknown_PARTNER2)
tab STATUS_PARTNER dumCITIZEN_Uknown_PARTNER2, m

*Citizenship Status 3

recode STATUS_PARTNER (1=1 "Canadian Citizen") (2/6=0 "Other")(7=.)(0
=.) (.=.), gen (dumCANADIAN_PARTNER3)
tab STATUS_PARTNER dumCANADIAN_PARTNER3, m

recode STATUS_PARTNER (2 3 4 5 6 =1 "Immigrant_PR_Refugee") (1=0
"Other")(7=.)(0=.)(.=.), gen (dumIMMIG_PR_REF_PARTNER3)
tab STATUS_PARTNER dumIMMIG_PR_REF_PARTNER3, m

**EDUCATION**

*Education2_Participant*

recode EDUC_PART (1 2=1 "Primary&Secondary") (3/8=0 "Other")(0=.)
(.=.), gen (dumPrimSEcEd_PARTIC2)
tab EDUC_PART dumPrimSEcEd_PARTIC2, m

recode EDUC_PART (3 4 5=1 "Trade/College") (1 2 6 7 8=0 "Other")(0=.)
(.=.), gen (dumTrade_DiplomaEd_PARTIC2)
tab EDUC_PART dumTrade_DiplomaEd_PARTIC2, m

recode EDUC_PART (6 7 8=1 "University or higher") (1/5=0 "Other")(0=.)
 (.=.), gen (dumUniversityEd_PARTIC2)
tab EDUC_PART dumUniversityEd_PARTIC2, m

*Education Partner 2*

recode EDUC_PARTNER (1 2=1 "Primary&Secondary") (3/9=0 "Other")(0=.)
(.=.), gen (dumPrimSEcEd_PARTNER2)
tab EDUC_PARTNER dumPrimSEcEd_PARTNER2, m

recode EDUC_PARTNER (3 4 5=1 "Trade/College") (1 2 6 7 8 9=0 "Other")(
0=.) (.=.), gen (dumTrade_DiplomaEd_PARTNER2)
tab EDUC_PARTNER dumTrade_DiplomaEd_PARTNER2, m

recode EDUC_PARTNER (6 7 8=1 "University or higher") (1 2 3 4 5 9=0
"Other")(0=.) (.=.), gen (dumUniversityEd_PARTNER2)
tab EDUC_PARTNER dumUniversityEd_PARTNER2, m

recode EDUC_PARTNER (9=1 "UnKnown") (1/8=0 "Other")(0=.) (.=.), gen (
```

```
recode EDUC_PARTNER (9=1 "UnKnown") (1/8=0 "Other")(0=.) (.=.), gen (dumUnknownED_PARTNER2)
tab EDUC_PARTNER dumUnknownED_PARTNER2, m

*Education Partner 3*

recode EDUC_PARTNER (1 2=1 "Primary&Secondary") (3/8=0 "Other")(9=.)(0=.) (.=.), gen (dumPrimSEcEd_PARTNER3)
tab EDUC_PARTNER dumPrimSEcEd_PARTNER3, m

recode EDUC_PARTNER (3 4 5=1 "Trade/College") (1 2 6 7 8=0 "Other")(9=.)(0=.) (.=.), gen (dumTrade_DiplomaEd_PARTNER3)
tab EDUC_PARTNER dumTrade_DiplomaEd_PARTNER3, m

recode EDUC_PARTNER (6 7 8=1 "University or higher") (1 2 3 4 5 =0 "Other")(9=.)(0=.) (.=.), gen (dumUniversityEd_PARTNER3)
tab EDUC_PARTNER dumUniversityEd_PARTNER3, m

**EMPLOYMENT**

*Employed during COVID*

*Participant
recode EMPL_PART_DURING (1=1 "Yes") (2 3=0 "Other")(0=.) (.=.), gen (dumEMPLOYEDPARTIC)
tab EMPL_PART_DURING dumEMPLOYEDPARTIC, m

recode EMPL_PART_DURING (2=1 "No") (1 3=0 "Other")(0=.) (.=.), gen (dumUNEMPLOYEDPARTIC)
tab EMPL_PART_DURING dumUNEMPLOYEDPARTIC, m

recode EMPL_PART_DURING (3=1 "Retired") (1 2=0 "Other")(0=.) (.=.), gen (dumRetiredPARTIC)
tab EMPL_PART_DURING dumRetiredPARTIC, m

*Participant Employment2*

recode EMPL_PART_DURING (1=1 "Yes") (2 3=0 "No & Retired")(0=.) (.=.), gen (dumEMPLOYEDPARTIC2)
tab EMPL_PART_DURING dumEMPLOYEDPARTIC2, m

recode EMPL_PART_DURING (2 3=1 "No & Retired") (1=0 "Yes")(0=.) (.=.), gen (dumUNEMPLOYEDPARTIC2)
tab EMPL_PART_DURING dumUNEMPLOYEDPARTIC2, m

*Partner*
recode EMP_PARTNER_DURING (1=1 "Yes") (2 3 4=0 "Other")(0=.) (.=.), gen (dumEMPLOYEDPARTNER)
```

```

recode EMP_PARTNER_DURING (2=1 "No") (1 3 4=0 "Other")(0=.) (.=.), gen (dumUNEMPLOYEDPARTNER)
tab EMP_PARTNER_DURING dumUNEMPLOYEDPARTNER, m

recode EMP_PARTNER_DURING (3=1 "Retired") (1 2 4=0 "Other")(0=.) (.=.), gen (dumRetiredPARTNER)
tab EMP_PARTNER_DURING dumRetiredPARTNER, m

recode EMP_PARTNER_DURING (4=1 "Unknown") (1 2 3=0 "Other")(0=.) (.=.), gen (dumUNKNOWNEMPLOYEDPARTNER)
tab EMP_PARTNER_DURING dumUNKNOWNEMPLOYEDPARTNER, m

*Partner Emp2*

recode EMP_PARTNER_DURING (1=1 "Yes") (2 3=0 "No& Retired")(4=.)(0=.) (.=.), gen (dumEMPLOYEDPARTNER2)
tab EMP_PARTNER_DURING dumEMPLOYEDPARTNER2, m

recode EMP_PARTNER_DURING (2 3 =1 "No") (1=0 "Other")(4=.)(0=.)(.=.), gen (dumUNEMPLOYEDPARTNER2)
tab EMP_PARTNER_DURING dumUNEMPLOYEDPARTNER2, m

*Household Income - unknown is missing (7)*

recode HH_INCOME (1 2=1 ">40,000") (3/6=0 "other") (7=.) (0=.) (.=.), gen (dumHHINCOMEUNDER40)
tab HH_INCOME dumHHINCOMEUNDER40, m

recode HH_INCOME (3 4 =1 "40 to 69,999") (1 2 5 6 =0 "Other") (7=.) (0 =.) (.=.), gen (dumHHINCOMEUNDER40_69)
tab HH_INCOME dumHHINCOMEUNDER40_69, m

recode HH_INCOME (5=1 "70-99,999") (1 2 3 4 6=0 "Other") (7=.) (0=.) (.=.), gen (dumHHINCOMEUNDER70_99)
tab HH_INCOME dumHHINCOMEUNDER70_99, m

recode HH_INCOME (6=1 "100,000PLUS") (1 2 3 4 5=0 "Other")(7=.)  (0=.) (.=.), gen (dumHHINCOMEUNDER100PLUS)
tab HH_INCOME dumHHINCOMEUNDER100PLUS, m

*geography 2

recode GEOGRAPHY (1=1 "Eastern Ontario") (2/6=0 "Other")(0=.) (.=.), gen (dumEASTONT2)
tab GEOGRAPHY dumEASTONT2, m

recode GEOGRAPHY (2=1 "Central Ontario") (1 3 4 5 6 7 8 9=0 "Other")(0
```

```
recode GEOGRAPHY (2=1 "Central Ontario") (1 3 4 5 6 7 8 9=0 "Other")(0=.) (.=.), gen (dumCENTRALONT2)
tab GEOGRAPHY dumCENTRALONT2, m

recode GEOGRAPHY (3 4=1 "Toronto &GTA") (1 2 5 6=0 "Other")(0=.) (.=.), gen (dumToronto_GTA2)
tab GEOGRAPHY dumToronto_GTA2, m

recode GEOGRAPHY (5=1 "Western Ontario") (1 2 3 4 6 =0 "Other")(0=.) (.=.), gen (dumWESTONT2)
tab GEOGRAPHY dumWESTONT2, m

recode GEOGRAPHY (6=1 "Northern Ontario") (1/5=0 "Other")(0=.) (.=.), gen (dumNORTHONT2)
tab GEOGRAPHY dumNORTHONT2, m

*Rural_Urban*

recode RURALURBAN (1=1 "Rural") (2=0 "Urban")(0=.) (.=.), gen (dumRURAL)
tab RURALURBAN dumRURAL, m

recode RURALURBAN (2=1 "Urban") (1=0 "Rural")(0=.) (.=.), gen (dumURBAN)
tab RURALURBAN dumURBAN, m


******OTHER FACTORS KNOWN TO IMPACT IPV***********

**DRINK-DRUGS-SOURCE OF STRAIN**

recode WISH_DRINK_COMBINED (3 4 5=1 "Always/Often/Sometimes") (1 2 =0 "Never/Seldom") (0=.) (.=.), gen (dumWishNoDrink)
tab WISH_DRINK_COMBINED dumWishNoDrink, m

recode WISH_DRINK_COMBINED (1 2=1 "Never/Seldom") (3 4 5=0 "Always/Often/Sometimes") (0=.) (.=.), gen (dumWishDrinkNEVER)
tab WISH_DRINK_COMBINED dumWishDrinkNEVER, m

recode STRAIN_DRINK_COMBINED (3 4 5=1 "Always/Often/Sometimes") (1 2 =0 "Never/Seldom") (0=.) (.=.), gen (dumSourceStrainDrink)
tab STRAIN_DRINK_COMBINED dumSourceStrainDrink, m

recode STRAIN_DRINK_COMBINED (1 2=1 "Never/Seldom") (3 4 5=0 "Always/Often/Sometimes") (0=.) (.=.), gen (dumNO_SourceStrainDrink)
tab STRAIN_DRINK_COMBINED dumNO_SourceStrainDrink, m

recode CON_LEAVE_DRINK_COMBINED (3 4 5=1 "Always/Often/Sometimes") (1
```

```
tab CON_LEAVE_DRINK_COMBINED dumConsLeaveDrink, m

recode CON_LEAVE_DRINK_COMBINED (1 2=1 "Never/Seldom") (3 4 5=0 "Always/Often/Sometimes") (0=.) (.=.), gen (dumNO_ConsLeaveDrink)
tab CON_LEAVE_DRINK_COMBINED dumNO_ConsLeaveDrink, m

recode WISH_DRUGS_COMBINED (3 4 5=1 "Always/Often/Sometimes") (1 2 =0 "Never/Seldom") (0=.) (.=.), gen (dumWishNoDrugs)
tab WISH_DRUGS_COMBINED dumWishNoDrugs, m

recode WISH_DRUGS_COMBINED (1 2=1 "Never/Seldom") (3 4 5=0 "Always/Often/Sometimes") (0=.) (.=.), gen (dumWishNoDrugsNEVER)
tab WISH_DRUGS_COMBINED dumWishNoDrugsNEVER, m

recode STRAIN_DRUGS_COMBINED (3 4 5=1 "Always/Often/Sometimes") (1 2 = 0 "Never/Seldom") (0=.) (.=.), gen (dumSourceStrainDrugs)
tab STRAIN_DRUGS_COMBINED dumSourceStrainDrugs, m

recode STRAIN_DRUGS_COMBINED (1 2=1 "Never/Seldom") (3 4 5=0 "Always/Often/Sometimes") (0=.) (.=.), gen (dumNO_SourceStrainDrugs)
tab STRAIN_DRUGS_COMBINED dumNO_SourceStrainDrugs, m

recode CON_LEAVE_DRUGS_COMBINED (3 4 5=1 "Always/Often/Sometimes") (1 2 =0 "Never/Seldom") (0=.) (.=.), gen (dumConsLeaveDrugs)
tab CON_LEAVE_DRUGS_COMBINED dumConsLeaveDrugs, m

recode CON_LEAVE_DRUGS_COMBINED (1 2=1 "Never/Seldom") (3 4 5=0 "Always/Often/Sometimes") (0=.) (.=.), gen (dumNO_ConsLeaveDrugs)
tab CON_LEAVE_DRUGS_COMBINED dumNO_ConsLeaveDrugs, m

* Sum the binary variables to create a composite score
gen substanceIssueScore = dumWishNoDrink + dumSourceStrainDrink + dumConsLeaveDrink + dumWishNoDrugs + dumSourceStrainDrugs + dumConsLeaveDrugs

* Define the impact level based on the sum score
gen substanceImpactLevel = "Low Impact" if substanceIssueScore <= 2
replace substanceImpactLevel = "Moderate Impact" if substanceIssueScore >= 3 & substanceIssueScore <= 4
replace substanceImpactLevel = "High Impact" if substanceIssueScore >= 5

* Tabulate the new categorized variable
tabulate substanceImpactLevel
encode substanceImpactLevel, generate(substanceImpactLevel_encoded)
tab substanceImpactLevel_encoded,m

recode substanceImpactLevel_encoded (1=1 "high impact" ) (2/4 =0
```

```
"Other")(0=.) (.=.), gen (dumSubstanceHIGHIMPACT)
tab substanceImpactLevel_encoded dumSubstanceHIGHIMPACT, m

recode substanceImpactLevel_encoded (2=1 "Low impact" ) (1 3 4 =0
"Other")(0=.) (.=.), gen (dumSubstanceLOWIMPACT)
tab substanceImpactLevel_encoded dumSubstanceLOWIMPACT, m

recode substanceImpactLevel_encoded (3=1 "Moderate impact" ) (1 2 4 =0
 "Other")(0=.) (.=.), gen (dumSubstanceModerateIMPACT)
tab substanceImpactLevel_encoded dumSubstanceModerateIMPACT, m

***OR - SOURCE OF STRAIN ***
codebook substanceImpactLevel, m
codebook substanceImpactLevel_encoded, m

recode substanceImpactLevel_encoded (1 3 = 1 "moderate/high") (2 = 0
"low")(0=.) (.=.), gen (dumSubstanceModHighIMPACT2)
tab substanceImpactLevel_encoded dumSubstanceModHighIMPACT2, m

recode substanceImpactLevel_encoded (2 = 1 "low") (1 3 = 0
"moderate/high")(0=.) (.=.), gen (dumSubstanceLowIMPACT2)
tab substanceImpactLevel_encoded dumSubstanceLowIMPACT2, m


**************************
*Information_during_COVID
**************************

recode INFORMATION_DURING (1=1 "yes") (2 3=0 "Other") (0=.) (.=.), gen
 (dumYESINFORMATION)
tab INFORMATION_DURING dumYESINFORMATION, m

recode INFORMATION_DURING (2=1 "No") (1 3=0 "Other") (0=.) (.=.), gen
(dumNOINFORMATION)
tab INFORMATION_DURING dumNOINFORMATION, m

recode INFORMATION_DURING (3=1 "I don't know") (1 2=0 "Other") (0=.)
(.=.), gen (dumNOSUREInformation)
tab INFORMATION_DURING dumNOSUREInformation, m

*Information 2

recode INFORMATION_DURING (1=1 "yes") (2 3=0 "No_dontknow") (0=.)
(.=.), gen (dumYESINFORMATION2)
tab INFORMATION_DURING dumYESINFORMATION2, m

recode INFORMATION_DURING (2 3=1 "No_Dontknow") (1=0 "Yes") (0=.)
(.=.), gen (dumNOINFORMATION2)
```

```
tab INFORMATION_DURING dumNOINFORMATION2, m

*Community Violence*

recode COMM_VIOL_DURING (1=1 "No") (2 3=0 "Other")(0=.) (.=.), gen (dumNO_COMMVIOLENCE)
tab COMM_VIOL_DURING dumNO_COMMVIOLENCE, m

recode COMM_VIOL_DURING (2=1 "Yes") (1 3=0 "Other")(0=.) (.=.), gen (dumYES_COMMVIOLENCE)
tab COMM_VIOL_DURING dumYES_COMMVIOLENCE, m

recode COMM_VIOL_DURING (3=1 "Not Sure") (1 2=0 "Other")(0=.) (.=.), gen (dumNotSure_COMMVIOLENCE)
tab COMM_VIOL_DURING dumNotSure_COMMVIOLENCE, m

**rolled up no and not sure

recode COMM_VIOL_DURING (1 3=1 "No") (2=0 "Yes")(0=.) (.=.), gen (dumNO_COMMVIOLENCE2)
tab COMM_VIOL_DURING dumNO_COMMVIOLENCE2, m

recode COMM_VIOL_DURING (2=1 "Yes") (1 3=0 "No")(0=.) (.=.), gen (dumYES_COMMVIOLENCE2)
tab COMM_VIOL_DURING dumYES_COMMVIOLENCE2, m


**Caregiving NUM_CHILD

recode NUM_CHILD (1 = 1 "None") (2/6 = 0 "Other") (0=.) (.=.), gen (dumNoChildren)
tab NUM_CHILD dumNoChildren, m

recode NUM_CHILD (2 3 4 = 1 "1 to 3") (1 5 6 = 0 "Other") (0=.) (.=.), gen (dum1_3_Children)
tab NUM_CHILD dum1_3_Children, m

recode NUM_CHILD (5 6 = 1 "4 +") (1 2 3 4 = 0 "Other") (0=.) (.=.), gen (dum4PlusChildren)
tab NUM_CHILD dum4PlusChildren, m

*NUM_CHILD_COMBINED

recode NUM_CHILD (1 = 1 "No_Children") (2/6 = 0 "Yes_Children") (0=.) (.=.), gen (dumNoChildren2)
tab NUM_CHILD dumNoChildren2, m

recode NUM_CHILD (2 3 4 5 6 = 1 "Yes_Children") (1 = 0 "No_Children")
```

```
recode NUM_CHILD (2 3 4 5 6 = 1 "Yes_Children") (1 = 0 "No_Children")
(0=.) (.=.), gen (dumYes_Children)
tab NUM_CHILD dumYes_Children, m


*PRIMARY_CAREGIVER_DURING

codebook PRIMARY_CAREGIVER_DURING
codebook CAREGIVING_nonCHILD

recode PRIMARY_CAREGIVER_DURING (1=1 "yes") (2=0 "No") (0=.) (.=.),
gen (dumCaregiverChiild)
tab PRIMARY_CAREGIVER_DURING dumCaregiverChiild, m

recode PRIMARY_CAREGIVER_DURING (2=1 "No") (1=0 "Yes") (0=.) (.=.),
gen (dumNotCaregiverChiild)
tab PRIMARY_CAREGIVER_DURING dumNotCaregiverChiild, m

recode CAREGIVING_nonCHILD (1=1 "yes") (2=0 "No") (0=.) (.=.), gen (
dumCaregiverNonChiild)
tab CAREGIVING_nonCHILD dumCaregiverNonChiild, m

recode CAREGIVING_nonCHILD (2=1 "No") (1=0 "Yes") (0=.) (.=.), gen (
dumNotCaregiverNonChiild)
tab CAREGIVING_nonCHILD dumNotCaregiverNonChiild, m

* Combine into a single caregiver variable
gen dumCaregiver = (dumCaregiverChiild == 1 | dumCaregiverNonChiild ==
 1)
label define caregiverlabel 0 "No" 1 "Yes"
label values dumCaregiver caregiverlabel

tab dumCaregiverChiild  dumCaregiverNonChiild
* Tabulate to check the new variable
tab dumCaregiver,m
codebook dumCaregiver

recode dumCaregiver (1=1 "yes") (0=0 "No") (.=.), gen (dumCaregiverYES
)
tab dumCaregiver dumCaregiverYES, m

recode dumCaregiver (0=1 "No") (1=0 "Yes") (.=.), gen (dumCaregiverNO)
tab dumCaregiver dumCaregiverNO, m

****GENDER ANALYSIS*******

***********************************
*****SOCIODEMOGRAPHIC DATA*********
```

```
***********************************

*Gender*

*Participant
codebook GENDER_PARTICIPANT

recode GENDER_PARTICIPANT (1=1 "Woman") (2 3 4 6 5 7 8 =0 "Other") (0
=.) (.=.), gen (dumWoman)
tab GENDER_PARTICIPANT dumWoman, m

recode GENDER_PARTICIPANT (2=1 "Man") (1 3 4 5 6 7 8=0 "Other") (0=.)
(.=.), gen (dumMan)
tab GENDER_PARTICIPANT dumMan, m

recode GENDER_PARTICIPANT (3 4 5 6 7 8=1 "Gender Diverse/PreferNotSay"
) (1 2=0 "Other") (0=.) (.=.), gen (dumGenders)
tab GENDER_PARTICIPANT dumGenders, m

*Participant 2 - with out prefer not to say

recode GENDER_PARTICIPANT (1=1 "Woman") (2 3 4 6 5 7 =0 "Other") (8
=.)(0=.) (.=.), gen (dumWoman2)
tab GENDER_PARTICIPANT dumWoman2, m

recode GENDER_PARTICIPANT (2=1 "Man") (1 3 4 5 6 7 =0 "Other")(8=.) (0
=.) (.=.), gen (dumMan2)
tab GENDER_PARTICIPANT dumMan2, m

recode GENDER_PARTICIPANT (3 4 5 6 7 =1 "genderdiverse") (1 2=0
"Other") (8=.)(0=.) (.=.), gen (dumGenders2)
tab GENDER_PARTICIPANT dumGenders2, m

**Participant 3 - women and men

recode GENDER_PARTICIPANT (1=1 "Woman") (2 =0 "Man")(3 =.)(4 =.)(5=.)(
6=.)(7=.)(8=.)(0=.)(.=.), gen (dumWoman3)
tab GENDER_PARTICIPANT dumWoman3, m

recode GENDER_PARTICIPANT (2=1 "Man") (1=0 "Woman")(3 =.)(4 =.)(5=.)(6
=.)(7=.)(8=.)(0=.)(.=.), gen (dumMan3)
tab GENDER_PARTICIPANT dumMan3, m

*Partner
recode GENDER_PARTNER (2=1 "Woman") (1 3 4 5 6 7 8 =0 "Other") (0=.)
(.=.), gen (dumWomanPartner)
tab GENDER_PARTNER dumWomanPartner, m
```

```
recode GENDER_PARTNER (1=1 "Man") (2 3 4 5 6 7 8=0 "Other") (0=.)
(.=.), gen (dumManPartner)
tab GENDER_PARTNER dumManPartner, m

recode GENDER_PARTNER (3 4 5 6 7 8=1 "GenderDiverse/PreferNotSay") (1
2=0 "Other") (0=.) (.=.), gen (dumGendersPartner)
tab GENDER_PARTNER dumGendersPartner, m

*Partner 2 without prefer not to say

recode GENDER_PARTNER (2=1 "Woman") (1 3 4 5 6 7 =0 "Other") (8=.)(0
=.)(.=.), gen (dumWomanPartner2)
tab GENDER_PARTNER dumWomanPartner2, m

recode GENDER_PARTNER (1=1 "Man") (2 3 4 5 6 7 =0 "Other")(8=.)(0
=.)(.=.), gen (dumManPartner2)
tab GENDER_PARTNER dumManPartner2, m

recode GENDER_PARTNER (3 4 5 6 7 =1 "genderdiverse") (1 2=0
"Man/Woman")(8=.)(0=.) (.=.), gen (dumGendersPartner2)
tab GENDER_PARTNER dumGendersPartner2, m

**Partner 3 - just women and men

recode GENDER_PARTNER (2=1 "Woman") (1 =0 "Man")(3 =.)(4 =.)(5=.)(6
=.)(7=.)(8=.)(0=.)(.=.), gen (dumWomanPartner3)
tab GENDER_PARTNER dumWomanPartner3, m

recode GENDER_PARTNER (1=1 "Man") (2=0 "Woman")(3 =.)(4 =.)(5=.)(6=.)(
7=.)(8=.)(0=.) (.=.), gen (dumManPartner3)
tab GENDER_PARTNER dumManPartner3, m

***Partner 4 - women and gender diverse

recode GENDER_PARTNER (2 3 4 5 6 7 =1 "Woman_Gender Diverse") (1 =0
"Man") (8=.)(0=.)(.=.), gen (dumWomanGDPartner4)
tab GENDER_PARTNER dumWomanGDPartner4, m

recode GENDER_PARTNER (1=1 "Man") (2 3 4 5 6 7 =0 "Woman_Gender
Diverse")(8=.)(0=.)(.=.), gen (dumManPartner4)
tab GENDER_PARTNER dumManPartner4, m

***** GENDER AND RELATIONSHIP TYPE ANALYSIS ****

* Step 1: Create a new variable for participant gender
gen new_genderParticipant = ""
replace new_genderParticipant = "woman" if GENDER_PARTICIPANT == 1
replace new_genderParticipant = "man" if GENDER_PARTICIPANT == 2
```

```
replace new_genderParticipant = "gender diverse" if inlist(GENDER_PARTICIPANT, 3, 4, 5, 6, 7)
replace new_genderParticipant = "prefer not to say" if GENDER_PARTICIPANT == 8
replace new_genderParticipant = "missing" if GENDER_PARTICIPANT == 0 | missing(GENDER_PARTICIPANT)

* Step 2: Create a new variable for partner gender
gen new_genderPartner = ""
replace new_genderPartner = "woman" if GENDER_PARTNER == 2
replace new_genderPartner = "man" if GENDER_PARTNER == 1
replace new_genderPartner = "gender diverse" if inlist(GENDER_PARTNER, 3, 4, 5, 6, 7)
replace new_genderPartner = "prefer not to say" if GENDER_PARTNER == 8
replace new_genderPartner = "missing" if GENDER_PARTNER == 0 | missing(GENDER_PARTNER)

* Step 3: Create a new variable for relationship type
gen relationship_type3 = .

* Step 4: Categorize heterosexual relationships as 1
replace relationship_type3 = 1 if (new_genderParticipant == "man" & new_genderPartner == "woman") | ///
                                  (new_genderParticipant == "woman" & new_genderPartner == "man")

* Step 5: Categorize same-sex relationships as 2
replace relationship_type3 = 2 if (new_genderParticipant == "man" & new_genderPartner == "man") | ///
                                  (new_genderParticipant == "woman" & new_genderPartner == "woman")

* Step 6: Categorize gender-diverse relationships as 3
replace relationship_type3 = 3 if (new_genderParticipant == "gender diverse" | new_genderPartner == "gender diverse")

* Step 7: Categorize "prefer not to say" as 4
replace relationship_type3 = 4 if (new_genderParticipant == "prefer not to say" | new_genderPartner == "prefer not to say")

* Step 8: Handle missing cases (keep as missing in relationship_type3)
replace relationship_type3 = . if (new_genderParticipant == "missing" | new_genderPartner == "missing")

* Step 9: Verify the distribution of relationship types
tabulate relationship_type3
```

```
* Step 10: Perform chi-square analysis for relationship type and IPV experience
*tabulate relationship_type3 dumYES_IPV, chi2 row
*tabulate relationship_type3 dumYES_IPV, exact row

* Step 11: Perform chi-square analysis for gender and IPV experience
*tabulate new_genderParticipant dumYES_IPV, chi2
*tabulate new_genderParticipant dumYES_IPV, exact

*tabulate relationship_type3 dumYES_IPV, chi2 row
*tabulate relationship_type3 dumYES_IPV, exact row

* Subset the data for "Prefer not to say" (relationship_type3 == 4)
*tabulate dumYES_IPV if relationship_type3 == 4, chi2 row
*tabulate dumYES_IPV if relationship_type3 == 4, exact row

codebook relationship_type3,m

recode relationship_type3 (1 = 1 "heterosexual") (2 3 4 = 0 "other") (.=.) (0=.), gen (dumheterosexual)
tab relationship_type3 dumheterosexual, m

recode relationship_type3 (2 3 = 1 "LGBTQ2+") (1 4 = 0 "other") (.=.) (0=.), gen (dumLGBTQ2)
tab relationship_type3 dumLGBTQ2, m

recode relationship_type3 (4 = 1 "PreferNotSay") (1 2 3 = 0 "other") (.=.) (0=.), gen (dumRelationprefnotsay)
tab relationship_type3 dumRelationprefnotsay, m

ttest dumheterosexual, by (dumYES_IPV)
ttest dumLGBTQ2, by (dumYES_IPV)
ttest dumRelationprefnotsay, by (dumYES_IPV)
ranksum dumRelationprefnotsay, by (dumYES_IPV)
tabulate dumRelationprefnotsay dumYES_IPV, exact

**AD HOC**
* Fisher's Exact Test: Heterosexual vs. Same-Sex
tabulate dumYES_IPV relationship_type3 if relationship_type3 == 1 | relationship_type3 == 2, exact

* Fisher's Exact Test: Heterosexual vs. Gender-Diverse
tabulate dumYES_IPV relationship_type3 if relationship_type3 == 1 | relationship_type3 == 3, exact

* Fisher's Exact Test: Heterosexual vs. Prefer Not to Say
tabulate dumYES_IPV relationship_type3 if relationship_type3 == 1 |
```

```
relationship_type3 == 4, exact

* Same-Sex vs. Gender-Diverse
tabulate dumYES_IPV relationship_type3 if relationship_type3 == 2 |
relationship_type3 == 3, exact

* Same-Sex vs. Prefer Not to Say
tabulate dumYES_IPV relationship_type3 if relationship_type3 == 2 |
relationship_type3 == 4, exact

* Gender-Diverse vs. Prefer Not to Say
tabulate dumYES_IPV relationship_type3 if relationship_type3 == 3 |
relationship_type3 == 4, exact


* Step 12: Create a binary variable for relationship type
gen binary_relationship_type = .

* Categorize Heterosexual relationships as 1
replace binary_relationship_type = 1 if relationship_type3 == 1

* Categorize LGBTQ2+/Unspecified relationships (Same-Sex,
Gender-Diverse, and Prefer Not to Say) as 2
replace binary_relationship_type = 2 if inlist(relationship_type3, 2,
3, 4)

* Handle missing cases
replace binary_relationship_type = . if missing(relationship_type3)

* Step 13: Verify the distribution of the binary variable
tabulate binary_relationship_type
codebook binary_relationship_type, m

recode binary_relationship_type (1 = 1 "Heterosexual") (2 = 0 "LGBTQ2"
) (.=.) (0=.) , gen (dumHeterosexual2)
tab binary_relationship_type dumHeterosexual2, m

recode binary_relationship_type (2 = 1 "LGBTQ2") (1 = 0 "Heterosexual"
) (.=.) (0=.), gen (dumLGBTQ2b)
tab binary_relationship_type dumLGBTQ2b, m

*Research Question*
*How do the health pathways and perceived outcomes differ between
women IPV Survivors and non-Survivors during COVID-19 lockdowns?*

*Focusing only on Women**
codebook GENDER_PARTICIPANT

```

```
*dropping men**
tab dumWoman3, m
drop if dumWoman3 == 0

*dropping gender diverse/did not want to disclose
tab dumWoman3, m
drop if dumWoman3 == .

tab dumWoman3, m
*653 women*

tab dumWoman3 dumYES_IPV, m
*503 = non-IPV; 150 Yes_IPV*


***because some of the categories had small sample size, we had to test using different methods for significance:
*Chi square
*tabulate variable group, chi2
*Fisher's exact
*tabulate variable group, exact
*ranksum
*ranksum variable, by(group)

*Age Participant*
codebook AGE_PART
gen age_group = .
replace age_group = 1 if inlist(AGE_PART, 1, 2)  // 18-34
replace age_group = 2 if inlist(AGE_PART, 3, 4)  // 35-54
replace age_group = 3 if inlist(AGE_PART, 5, 6)  // 55+
label define age_labels 1 "18-34" 2 "35-54" 3 "55+"
label values age_group age_labels
tab AGE_PART age_group, missing

tabulate age_group dumYES_IPV, chi2
tab age_group dumYES_IPV, chi2 expected cell
prtest IPV_EXP if age_group == 1, by(IPV_EXP)
prtest dumYES_IPV if age_group == 2, by(dumYES_IPV)
prtest dumYES_IPV if age_group == 3, by(dumYES_IPV)


*Relationship Type*
codebook relationship_type3
gen relationship = .
replace relationship = 1 if inlist(relationship_type3, 1)  // Heterosexual
replace relationship = 2 if inlist(relationship_type3, 2, 3)  // LGBTQ2+
```

```
replace relationship = 3 if inlist(relationship_type3, 4)  // Prefer not to say
label define relationship_labels 1 "Heterosexual" 2 "LGBTQ2+" 3 "NotDisclosed"
label values relationship relationship_labels
tab relationship_type3 relationship, m

tabulate relationship dumYES_IPV, exact

*Race participant*

codebook RACE_PARTICIPANT_2
gen race_participant = .
replace race_participant = 1 if inlist(RACE_PARTICIPANT_2, 1, 3, 4, 5, 6, 7, 8, 9, 13)  // racialized
replace race_participant = 2 if inlist(RACE_PARTICIPANT_2, 2)  // white
replace race_participant = 3 if inlist(RACE_PARTICIPANT_2, 10, 11)  // Prefer not to say
label define raceparticipant_labels 1 "racialized" 2 "white" 3 "NotDisclosed"
label values race_participant raceparticipant_labels
tab RACE_PARTICIPANT_2 race_participant, m
tabulate race_participant dumYES_IPV, chi2

*Immigration Status
codebook CITIZEN_STATUS
gen participant_citizenship = .
replace participant_citizenship = 1 if inlist(CITIZEN_STATUS, 1)  // Canadian
replace participant_citizenship = 2 if inlist(CITIZEN_STATUS, 2, 3, 4, 5, 6)  // Immigrant/Refugee/PR
replace participant_citizenship = 3 if inlist(CITIZEN_STATUS, 7)  // Prefer not to say
label define citizenshipparticipant_labels 1 "Canadian" 2 "Immigrant/Refugee/PR" 3 "Prefer not to say"
label values participant_citizenship citizenshipparticipant_labels
tab CITIZEN_STATUS participant_citizenship, m

tabulate participant_citizenship dumYES_IPV, exact

**Education participant*

codebook EDUC_PART
gen participant_education = .
replace participant_education = 1 if inlist(EDUC_PART, 1,2)  // Primary/Secondary
replace participant_education = 2 if inlist(EDUC_PART, 3,4,5)  //
```

```
Trade/College
replace participant_education = 3 if inlist(EDUC_PART, 6,7,8)  // University+
label define participanteduc_labels 1 " Primary/Secondary" 2 " Trade/College" 3 " University+"
label values participant_education participanteduc_labels
tab EDUC_PART participant_education, m

tabulate participant_education dumYES_IPV, chi2

**Employment participant*
codebook EMPL_PART_DURING
gen participant_employment = .
replace participant_employment = 1 if inlist(EMPL_PART_DURING, 1)  // Employed
replace participant_employment = 2 if inlist(EMPL_PART_DURING, 2,3)  // Unemployed
label define participantemp_labels 1 " Employed" 2 " Unemployed"
label values participant_employment participantemp_labels
tab EMPL_PART_DURING participant_employment, m

tabulate participant_employment dumYES_IPV, chi2

**Partner Gender**
codebook GENDER_PARTNER
gen partner_gender = .
replace partner_gender = 1 if inlist(GENDER_PARTNER, 1)  // Man
replace partner_gender = 2 if inlist(GENDER_PARTNER, 2)  // Woman
replace partner_gender = 3 if inlist(GENDER_PARTNER, 3, 4, 5, 6, 7, 8)  // Gender Diverse
label define partnergender_labels 1 " Man" 2 " Woman" 3 "Gender Diverse"
label values partner_gender partnergender_labels
tab GENDER_PARTNER partner_gender, m

tabulate partner_gender dumYES_IPV, exact

*Age partner*
codebook AGE_PARTNER
gen partner_age_group = .
replace partner_age_group = 1 if inlist(AGE_PARTNER, 1, 2)  // 18-34
replace partner_age_group = 2 if inlist(AGE_PARTNER, 3, 4)  // 35-54
replace partner_age_group = 3 if inlist(AGE_PARTNER, 5, 6)  // 55+
label define partnerage_labels 1 "18-34" 2 "35-54" 3 "55+"
label values partner_age_group partnerage_labels
tab AGE_PARTNER partner_age_group, missing

tabulate partner_age_group dumYES_IPV, chi2
```

```
*Partner Race*
codebook RACE_PARTNER_2
gen race_partner = .
replace race_partner = 1 if inlist(RACE_PARTNER_2, 1, 3, 4, 5, 6, 7, 8, 9, 13)  // racialized
replace race_partner = 2 if inlist(RACE_PARTNER_2, 2)  // white
replace race_partner = 3 if inlist(RACE_PARTNER_2, 10, 11)  // Prefer not to say
label define racepartner_labels 1 "racialized" 2 "white" 3 "NotDisclosed"
label values race_partner racepartner_labels
tab RACE_PARTNER_2 race_partner, m

tabulate race_partner dumYES_IPV, exact

*Partner Education
codebook EDUC_PARTNER
gen partner_education = .
replace partner_education = 1 if inlist(EDUC_PARTNER, 1,2)  // Primary/Secondary
replace partner_education = 2 if inlist(EDUC_PARTNER, 3,4,5)  // Trade/College
replace partner_education = 3 if inlist(EDUC_PARTNER, 6,7,8)  // University+
label define partnereduc_labels 1 " Primary/Secondary" 2 " Trade/College" 3 " University+"
label values partner_education partnereduc_labels
tab EDUC_PARTNER partner_education, m

tabulate partner_education dumYES_IPV, chi2

*Status partner*

codebook STATUS_PARTNER
gen partner_citizenship = .
replace partner_citizenship = 1 if inlist(STATUS_PARTNER, 1)  // Canadian
replace partner_citizenship = 2 if inlist(STATUS_PARTNER, 2, 3, 4, 5, 6)  // Immigrant/Refugee/PR
replace partner_citizenship = 3 if inlist(STATUS_PARTNER, 7)  // Unknown
label define statuspartner_labels 1 "Canadian" 2 "Immigrant/Refugee/PR" 3 "Unknown"
label values partner_citizenship statuspartner_labels
tab STATUS_PARTNER partner_citizenship, m

tabulate partner_citizenship dumYES_IPV, exact
```

```
*Partner employment

codebook EMP_PARTNER_DURING
gen partner_employment = .
replace partner_employment = 1 if inlist(EMP_PARTNER_DURING, 1)  // Employed
replace partner_employment = 2 if inlist(EMP_PARTNER_DURING, 2,3)  // Unemployed
label define partneremployment_labels 1 " Employed" 2 " Unemployed"
label values partner_employment partneremployment_labels
tab EMP_PARTNER_DURING partner_employment, m

tabulate partner_employment dumYES_IPV, chi2

*householde income
codebook HH_INCOME
gen hhincome = .
replace hhincome = 1 if inlist(HH_INCOME, 1, 2)  // <40,000
replace hhincome = 2 if inlist(HH_INCOME, 3, 4)  // 40-69,999
replace hhincome = 3 if inlist(HH_INCOME, 5)  // 70,99,999
replace hhincome = 4 if inlist(HH_INCOME, 6)  // >100,000
label define income_labels 1 "<40,000" 2 "40-69,999" 3 "70,99,999" 4 ">100,000"
label values hhincome income_labels
tab HH_INCOME hhincome, m

tabulate hhincome dumYES_IPV, chi2

*Geography
codebook GEOGRAPHY
gen geography = .
replace geography = 1 if inlist(GEOGRAPHY, 1)  // Eastern
replace geography = 2 if inlist(GEOGRAPHY, 2)  // Central
replace geography = 3 if inlist(GEOGRAPHY, 3, 4)  // Toronto & GTA
replace geography = 4 if inlist(GEOGRAPHY, 5)  // Western
replace geography = 5 if inlist(GEOGRAPHY, 6)  // Northern
label define geography_labels 1 "Eastern" 2 "Central" 3 "Toronto/GTA" 4 "Wester" 5 "Northern"
label values geography geography_labels
tab GEOGRAPHY geography, m

tabulate geography dumYES_IPV, chi2

*Rural/Urban
codebook RURALURBAN
gen ruralurban = .
replace ruralurban = 1 if inlist(RURALURBAN, 1)  // Rural
```

```
replace ruralurban = 2 if inlist(RURALURBAN, 2)  // Urban
label define ruralurban_labels 1 "Rural" 2 "Urban"
label values ruralurban ruralurban_labels
tab RURALURBAN ruralurban, m
tabulate RURALURBAN dumYES_IPV, chi2

*Community violence
codebook COMM_VIOL_DURING
gen communityviolence = .
replace communityviolence = 1 if inlist(COMM_VIOL_DURING, 1,3)  // No
replace communityviolence = 2 if inlist(COMM_VIOL_DURING, 2)  // Yes
label define communityviolence_labels 1 "No" 2 "Yes"
label values communityviolence communityviolence_labels
tab COMM_VIOL_DURING communityviolence, m
tabulate communityviolence dumYES_IPV, chi2

**Infomration during Covid
codebook INFORMATION_DURING
gen information = .
replace information = 1 if inlist(INFORMATION_DURING, 1)  // yes
replace information = 2 if inlist(INFORMATION_DURING, 2, 3)  // no/not sure
label define information_labels 1 "Yes" 2 "No"
label values information information_labels
tab INFORMATION_DURING information, m
tabulate information dumYES_IPV, chi2

**Impact of substance use
codebook substanceImpactLevel
* Generate a numeric variable
gen substanceImpactNum = .
replace substanceImpactNum = 1 if substanceImpactLevel == "High Impact"
replace substanceImpactNum = 3 if substanceImpactLevel == "Moderate Impact"
replace substanceImpactNum = 2 if substanceImpactLevel == "Low Impact"

gen substanceImpactBinary = .
replace substanceImpactBinary = 1 if inlist(substanceImpactLevel, "High Impact", "Moderate Impact")
replace substanceImpactBinary = 2 if substanceImpactLevel == "Low Impact"

tabulate substanceImpactLevel substanceImpactNum, missing
tabulate substanceImpactLevel substanceImpactBinary, missing

tabulate substanceImpactBinary dumYES_IPV, chi2
```

```
tabulate substanceImpactBinary dumYES_IPV, chi2

*Number of Children
gen has_children = .
replace has_children = 0 if NUM_CHILD == 1  // None (No Children)
replace has_children = 1 if inlist(NUM_CHILD, 2, 3, 4, 5, 6)  // Has at least 1 child

label variable has_children "Has Children (0 = No, 1 = Yes)"
label define hasChildrenLabel 0 "No Children" 1 "Has Children"
label values has_children hasChildrenLabel
tabulate NUM_CHILD has_children, missing
tabulate has_children dumYES_IPV, chi2

*Informal Caregiver*

tabulate dumCaregiver dumYES_IPV, chi2


*Health Behaviour

tabulate ALCOHOL_DURING dumYES_IPV if ALCOHOL_DURING > 0, chi2

tabulate TOBAC_DRUING dumYES_IPV if TOBAC_DRUING > 0, chi2

tabulate CANNAB_DURING dumYES_IPV if CANNAB_DURING > 0, exact

tabulate SUBSTANCE_DURING dumYES_IPV if SUBSTANCE_DURING > 0, exact

tabulate TV_DURING dumYES_IPV if TV_DURING > 0, chi2

tabulate INTERNET_DURING dumYES_IPV if INTERNET_DURING > 0, chi2

tabulate EXERCISING_DURING dumYES_IPV if EXERCISING_DURING > 0, chi2

tabulate EATJUNK_DURING dumYES_IPV if EATJUNK_DURING > 0, chi2

tabulate INFORMAL_DURING dumYES_IPV if INFORMAL_DURING > 0, chi2

tabulate FORMAL_DURING dumYES_IPV if FORMAL_DURING > 0, chi2

tabulate SLEEP_DURING dumYES_IPV if SLEEP_DURING > 0, chi2


*Health outcomes

tabulate BadMentHealth dumYES_IPV, chi2
tabulate BadPhysHealth dumYES_IPV, chi2
```

```
tabulate GoodPhysHealth dumYES_IPV, chi2
tabulate GoodMentHealth dumYES_IPV, chi2

ttest BadMentHealth, by (dumYES_IPV)
ttest BadPhysHealth, by (dumYES_IPV)
ttest GoodPhysHealth, by (dumYES_IPV)
ttest GoodMentHealth, by (dumYES_IPV)


ttest

*DESCRIPTIVES TABLE 1*

*FOR IPV=0 NO IPV*:

* Set up the Excel file for output
putexcel set "my_results_NO_IPV_Jan_23_Women.xlsx", replace

* Set headers in the first row
putexcel A1 = "Variable" B1 = "Mean (%)" C1 = "Lower Bound (%)" D1 = "Upper Bound (%)" E1 = "T-Statistic" F1 = "P-Value" G1 = "Sample Size (n)"

* Define a list of variables and initialize row counter for Excel output
local row = 2

* Loop through each variable
foreach var in dumAgePARTIC_18_34 dumAgePARTIC_35_54 dumAgePARTIC_55Plus dumHeterosexual dumLGBTQ2 dumRelationprefnotsay dumRacializedPARTIC dumWHITEPARTIC dumPrefNotSayRACEPARTIC3 dumCANADIAN_PARTIC3 dumIMMIG_PR_REF_PARTIC3 dumPrimSEcEd_PARTIC2 dumTrade_DiplomaEd_PARTIC2 dumUniversityEd_PARTIC2 dumEMPLOYEDPARTIC2 dumUNEMPLOYEDPARTIC2 dumWomanPartner dumManPartner dumGendersPartner dumAgePARTNER_18_34 dumAgePARTNER_35_54 dumAgePARTNER_55Plus dumWHITEPARTNER dumRacializedPARTNER dumPrefNotSayRACEPARNER dumCANADIAN_PARTNER3 dumIMMIG_PR_REF_PARTNER3 dumPrimSEcEd_PARTNER3 dumTrade_DiplomaEd_PARTNER3 dumUniversityEd_PARTNER3 dumEMPLOYEDPARTNER2 dumUNEMPLOYEDPARTNER2 dumHHINCOMEUNDER40 dumHHINCOMEUNDER40_69 dumHHINCOMEUNDER70_99 dumHHINCOMEUNDER100PLUS dumEASTONT2 dumCENTRALONT2 dumToronto_GTA2 dumWESTONT2 dumNORTHONT2 dumRURAL dumURBAN dumNO_COMMVIOLENCE dumYES_COMMVIOLENCE dumNotSure_COMMVIOLENCE dumYESINFORMATION2 dumNOINFORMATION2 dumSubstanceModHighIMPACT2 dumSubstanceLowIMPACT2 dumNoChildren2 dumYes_Children dumCaregiverYES dumCaregiverNO {
    * Compute CI for the variable if IPV=yes
    ci means `var' if dumYES_IPV==0, level(95)
    local mean = r(mean) * 100
```

```
    local mean = r(mean) * 100
    local lb = r(lb) * 100
    local ub = r(ub) * 100
    local n = r(N)

    * Compute t-test for the variable by IPV status
    ttest `var', by(dumYES_IPV)
    local tstat = r(t)
    local pval = r(p)

    * Write results to Excel, formatted as percentages where applicable
    putexcel A`row' = "`var'" B`row' = `mean' C`row' = `lb' D`row' = `ub' E`row' = `tstat' F`row' = `pval' G`row' = `n'

    * Increment row counter
    local row = `row' + 1
}

* Save the Excel file
putexcel save

*FOR IPV=1 YES IPV*:

* Set up the Excel file for output
putexcel set "my_results_YES_IPV_Jan_23_Women.xlsx", replace

* Set headers in the first row
putexcel A1 = "Variable" B1 = "Mean (%)" C1 = "Lower Bound (%)" D1 = "Upper Bound (%)" E1 = "T-Statistic" F1 = "P-Value" G1 = "Sample Size (n)"

* Define a list of variables and initialize row counter for Excel output
local row = 2

* Loop through each variable
foreach var in dumAgePARTIC_18_34 dumAgePARTIC_35_54 dumAgePARTIC_55Plus dumHeterosexual dumLGBTQ2 dumRelationprefnotsay dumRacializedPARTIC dumWHITEPARTIC dumPrefNotSayRACEPARTIC3 dumCANADIAN_PARTIC3 dumIMMIG_PR_REF_PARTIC3 dumPrimSEcEd_PARTIC2 dumTrade_DiplomaEd_PARTIC2 dumUniversityEd_PARTIC2 dumEMPLOYEDPARTIC2 dumUNEMPLOYEDPARTIC2 dumWomanPartner dumManPartner dumGendersPartner dumAgePARTNER_18_34 dumAgePARTNER_35_54 dumAgePARTNER_55Plus dumWHITEPARTNER dumRacializedPARTNER dumPrefNotSayRACEPARNER dumCANADIAN_PARTNER3 dumIMMIG_PR_REF_PARTNER3 dumPrimSEcEd_PARTNER3 dumTrade_DiplomaEd_PARTNER3 dumUniversityEd_PARTNER3 dumEMPLOYEDPARTNER2 dumUNEMPLOYEDPARTNER2 dumHHINCOMEUNDER40
```

```
dumEMPLOYEDPARTNER2 dumUNEMPLOYEDPARTNER2 dumHHINCOMEUNDER40
dumHHINCOMEUNDER40_69 dumHHINCOMEUNDER70_99 dumHHINCOMEUNDER100PLUS
dumEASTONT2 dumCENTRALONT2 dumToronto_GTA2 dumWESTONT2 dumNORTHONT2
dumRURAL dumURBAN dumNO_COMMVIOLENCE dumYES_COMMVIOLENCE
dumNotSure_COMMVIOLENCE dumYESINFORMATION2 dumNOINFORMATION2
dumSubstanceModHighIMPACT2 dumSubstanceLowIMPACT2 dumNoChildren2
dumYes_Children dumCaregiverYES dumCaregiverNO {
    * Compute CI for the variable if IPV=yes
    ci means `var' if dumYES_IPV==1, level(95)
    local mean = r(mean) * 100
    local lb = r(lb) * 100
    local ub = r(ub) * 100
    local n = r(N)

    * Compute t-test for the variable by IPV status
    ttest `var', by(dumYES_IPV)
    local tstat = r(t)
    local pval = r(p)

    * Write results to Excel, formatted as percentages where
applicable
    putexcel A`row' = "`var'" B`row' = `mean' C`row' = `lb' D`row' =
`ub' E`row' = `tstat' F`row' = `pval' G`row' = `n'

    * Increment row counter
    local row = `row' + 1
}

* Save the Excel file
putexcel save


*DESCRIPTIVES TABLE HEALTH OUTCOMES*

*FOR IPV=0 NO IPV*:

* Set up the Excel file for output
putexcel set "Health Behavrious_NO_IPV_Jan_23.xlsx", replace

* Set headers in the first row
putexcel A1 = "Variable" B1 = "Mean (%)" C1 = "Lower Bound (%)" D1 =
"Upper Bound (%)" E1 = "T-Statistic" F1 = "P-Value" G1 = "Sample Size
(n)"

* Define a list of variables and initialize row counter for Excel
output
local row = 2

```

```
* Loop through each variable
foreach var in AlcoholIncreased AlcoholDecreased AlcoholNoChange TOBACIncreased TOBACDecreased TOBACNoChange CANNABIncreased CANNABDecreased CANNABNoChange SUBSTANCEIncreased SUBSTANCEDecreased SUBSTANCENoChange TV_Increased TV_Decreased TV_NoChange INTERNET_Increased INTERNET_Decreased INTERNET_NoChange EXERCISE_Increased EXECRISE_Decreased EXERCISE_NoChange JUNK_Increased  JUNK_Decreased JUNK_NoChange   SeekingINFIncreased SeekingINFdecreased NoChangeInSeekingINF SeekingFORMIncreased SeekingFORMdecreased NoChangeInSeekingFORM QualitySLEEPIncreased QualitySLEEPdecreased NoChangeInSLEEP BadMentHealth GoodMentHealth BadPhysHealth GoodPhysHealth {
    * Compute CI for the variable if IPV=yes
    ci means `var' if dumYES_IPV==0, level(95)
    local mean = r(mean) * 100
    local lb = r(lb) * 100
    local ub = r(ub) * 100
    local n = r(N)

    * Compute t-test for the variable by IPV status
    ttest `var', by(dumYES_IPV)
    local tstat = r(t)
    local pval = r(p)

    * Write results to Excel, formatted as percentages where applicable
    putexcel A`row' = "`var'" B`row' = `mean' C`row' = `lb' D`row' = `ub' E`row' = `tstat' F`row' = `pval' G`row' = `n'

    * Increment row counter
    local row = `row' + 1
}

* Save the Excel file
putexcel save

*FOR IPV=1 YES IPV*:

* Set up the Excel file for output
putexcel set "Health Behavrious_YES_IPV_Jan_23.xlsx", replace

* Set headers in the first row
putexcel A1 = "Variable" B1 = "Mean (%)" C1 = "Lower Bound (%)" D1 = "Upper Bound (%)" E1 = "T-Statistic" F1 = "P-Value" G1 = "Sample Size (n)"

* Define a list of variables and initialize row counter for Excel output
```

```
local row = 2

* Loop through each variable
foreach var in AlcoholIncreased AlcoholDecreased AlcoholNoChange TOBACIncreased TOBACDecreased TOBACNoChange CANNABIncreased CANNABDecreased CANNABNoChange SUBSTANCEIncreased SUBSTANCEDecreased SUBSTANCENoChange TV_Increased TV_Decreased TV_NoChange INTERNET_Increased INTERNET_Decreased INTERNET_NoChange EXERCISE_Increased EXECRISE_Decreased EXERCISE_NoChange JUNK_Increased  JUNK_Decreased JUNK_NoChange   SeekingINFIncreased SeekingINFdecreased NoChangeInSeekingINF SeekingFORMIncreased SeekingFORMdecreased NoChangeInSeekingFORM QualitySLEEPIncreased QualitySLEEPdecreased NoChangeInSLEEP BadMentHealth GoodMentHealth BadPhysHealth GoodPhysHealth {
    * Compute CI for the variable if IPV=yes
    ci means `var' if dumYES_IPV==1, level(95)
    local mean = r(mean) * 100
    local lb = r(lb) * 100
    local ub = r(ub) * 100
    local n = r(N)

    * Compute t-test for the variable by IPV status
    ttest `var', by(dumYES_IPV)
    local tstat = r(t)
    local pval = r(p)

    * Write results to Excel, formatted as percentages where applicable
    putexcel A`row' = "`var'" B`row' = `mean' C`row' = `lb' D`row' = `ub' E`row' = `tstat' F`row' = `pval' G`row' = `n'

    * Increment row counter
    local row = `row' + 1
}

* Save the Excel file
putexcel save


***because some of the categories had small sample size, we had to test using different methods for significance:
*Chi square
tabulate variable group, chi2
*Fisher's exact
tabulate variable group, exact
*ranksum
ranksum variable, by(group)

```

```
ranksum dumAgePARTIC_18_34, by (dumYES_IPV)
ranksum dumAgePARTIC_35_54, by (dumYES_IPV)
ranksum dumAgePARTIC_55Plus, by (dumYES_IPV)
tabulate dumAgePARTIC_18_34 dumYES_IPV
tabulate dumAgePARTIC_35_54 dumYES_IPV
tabulate dumAgePARTIC_55Plus dumYES_IPV

tabulate relationship_type3 dumYES_IPV, exact

tabulate RACE_PARTICIPANT_2 dumYES_IPV, chi2

tabulate CITIZEN_STATUS dumYES_IPV, exact

tabulate EDUC_PART dumYES_IPV, chi2

tabulate EMPL_PART_DURING dumYES_IPV, chi2

tabulate GENDER_PARTNER dumYES_IPV, exact


ranksum dumAgePARTNER_18_34, by (dumYES_IPV)
ranksum dumAgePARTNER_35_54, by (dumYES_IPV)
ranksum dumAgePARTNER_55Plus, by (dumYES_IPV)
tabulate dumAgePARTNER_18_34 dumYES_IPV
tabulate dumAgePARTNER_35_54 dumYES_IPV
tabulate dumAgePARTNER_55Plus dumYES_IPV

tabulate RACE_PARTNER dumYES_IPV, exact

tabulate EDUC_PARTNER dumYES_IPV, chi2

tabulate STATUS_PARTNER dumYES_IPV, exact

tabulate EMP_PARTNER_DURING dumYES_IPV, chi2

ranksum dumHHINCOMEUNDER40 , by (dumYES_IPV)
ranksum dumHHINCOMEUNDER40_69, by (dumYES_IPV)
ranksum dumHHINCOMEUNDER70_99, by (dumYES_IPV)
ranksum dumHHINCOMEUNDER100PLUS, by (dumYES_IPV)
tabulate dumHHINCOMEUNDER40 dumYES_IPV
tabulate dumHHINCOMEUNDER40_69 dumYES_IPV
tabulate dumHHINCOMEUNDER70_99 dumYES_IPV
tabulate dumHHINCOMEUNDER100PLUS dumYES_IPV


tabulate GEOGRAPHY dumYES_IPV, chi2
prtest dumEASTONT2, by(dumYES_IPV)
prtest dumCENTRALONT2, by(dumYES_IPV)
```

```
prtest dumToronto_GTA2, by(dumYES_IPV)
prtest dumWESTONT2, by(dumYES_IPV)
prtest dumNORTHONT2, by(dumYES_IPV)

tabulate RURALURBAN dumYES_IPV, chi2

tabulate COMM_VIOL_DURING dumYES_IPV, exact

tabulate INFORMATION_DURING dumYES_IPV, chi2

tabulate substanceImpactLevel dumYES_IPV, chi2

tabulate NUM_CHILD dumYES_IPV, exact
codebook NUM_CHILD

tabulate dumCaregiver dumYES_IPV, chi2


*Health Behaviour


tabulate ALCOHOL_DURING dumYES_IPV if ALCOHOL_DURING > 0, chi2
prtest AlcoholIncreased, by(dumYES_IPV)
prtest AlcoholDecreased, by(dumYES_IPV)
prtest AlcoholNoChange, by(dumYES_IPV)

tabulate TOBAC_DRUING dumYES_IPV if TOBAC_DRUING > 0, chi2
prtest TOBACIncreased, by(dumYES_IPV)
prtest TOBACDecreased, by(dumYES_IPV)
prtest TOBACNoChange, by(dumYES_IPV)

tabulate CANNAB_DURING dumYES_IPV if CANNAB_DURING > 0, chi2
prtest CANNABIncreased, by(dumYES_IPV)
prtest CANNABDecreased, by(dumYES_IPV)
prtest CANNABNoChange, by(dumYES_IPV)

tabulate SUBSTANCE_DURING dumYES_IPV if SUBSTANCE_DURING > 0, exact
prtest SUBSTANCEIncreased, by(dumYES_IPV)
prtest SUBSTANCEDecreased, by(dumYES_IPV)
prtest SUBSTANCENoChange, by(dumYES_IPV)

tabulate TV_DURING dumYES_IPV if TV_DURING > 0, chi2
prtest TV_Increased, by(dumYES_IPV)
prtest TV_Decreased, by(dumYES_IPV)
prtest TV_NoChange, by(dumYES_IPV)

tabulate INTERNET_DURING dumYES_IPV if INTERNET_DURING > 0, chi2
prtest INTERNET_Increased, by(dumYES_IPV)
```

```
prtest INTERNET_Decreased, by(dumYES_IPV)
prtest INTERNET_NoChange, by(dumYES_IPV)


tabulate EXERCISING_DURING dumYES_IPV if EXERCISING_DURING > 0, chi2
prtest EXERCISE_Increased, by(dumYES_IPV)
prtest EXECRISE_Decreased, by(dumYES_IPV)
prtest EXERCISE_NoChange, by(dumYES_IPV)


tabulate EATJUNK_DURING dumYES_IPV if EATJUNK_DURING > 0, chi2
prtest JUNK_Increased, by(dumYES_IPV)
prtest JUNK_Decreased, by(dumYES_IPV)
prtest JUNK_NoChange, by(dumYES_IPV)

tabulate INFORMAL_DURING dumYES_IPV if INFORMAL_DURING > 0, chi2
prtest SeekingINFIncreased, by(dumYES_IPV)
prtest SeekingINFdecreased, by(dumYES_IPV)
prtest NoChangeInSeekingINF, by(dumYES_IPV)

tabulate FORMAL_DURING dumYES_IPV if FORMAL_DURING > 0, chi2
prtest SeekingFORMIncreased, by(dumYES_IPV)
prtest SeekingFORMdecreased, by(dumYES_IPV)
prtest NoChangeInSeekingFORM, by(dumYES_IPV)

tabulate SLEEP_DURING dumYES_IPV if SLEEP_DURING > 0, chi2
prtest QualitySLEEPIncreased, by(dumYES_IPV)
prtest QualitySLEEPdecreased, by(dumYES_IPV)
prtest NoChangeInSLEEP, by(dumYES_IPV)

*RELATIVE RISK

*ALCOHOL*
* Recode alcohol so No change = 0 (base), Increased = 1, Decreased = 2
recode ALCOHOL_DURING (3=0 "No Change") (1=1 "Increased") (2=2
"Decreased"), gen(alcohol_change)
label define alc 0 "No Change" 1 "Increased" 2 "Decreased", replace
label values alcohol_change alc

* Multinomial logistic regression
mlogit alcohol_change i.dumYES_IPV i.age_group i.hhincome ///
       i.participant_education i.participant_employment ///
       i.has_children i.dumCaregiver, baseoutcome(0) vce(robust)

* Show relative risk ratios instead of log-odds
mlogit, rrr
```

```
* Predicted probabilities of each category by IPV
margins dumYES_IPV, predict(outcome(0))   // No change
margins dumYES_IPV, predict(outcome(1))   // Increased
margins dumYES_IPV, predict(outcome(2))   // Decreased

* Differences between IPV and non-IPV
margins r.dumYES_IPV, predict(outcome(0))   // No change diff
margins r.dumYES_IPV, predict(outcome(1))   // Increased diff
margins r.dumYES_IPV, predict(outcome(2))   // Decreased diff

*TOBACCO*

* Recode alcohol so No change = 0 (base), Increased = 1, Decreased = 2
recode TOBAC_DRUING (3=0 "No Change") (1=1 "Increased") (2=2 "Decreased"), gen(tobac_change)
label define tobac 0 "No Change" 1 "Increased" 2 "Decreased", replace
label values tobac_change tobac

* Multinomial logistic regression
mlogit tobac_change i.dumYES_IPV i.age_group i.hhincome ///
       i.participant_education i.participant_employment ///
       i.has_children i.dumCaregiver, baseoutcome(0) vce(robust)

* Show relative risk ratios instead of log-odds
mlogit, rrr


* Predicted probabilities of each category by IPV
margins dumYES_IPV, predict(outcome(0))   // No change
margins dumYES_IPV, predict(outcome(1))   // Increased
margins dumYES_IPV, predict(outcome(2))   // Decreased

* Differences between IPV and non-IPV
margins r.dumYES_IPV, predict(outcome(0))   // No change diff
margins r.dumYES_IPV, predict(outcome(1))   // Increased diff
margins r.dumYES_IPV, predict(outcome(2))   // Decreased diff


*---------------------------------------------------------------------*
* CANNABIS
*---------------------------------------------------------------------*
* Recode cannabis so No change = 0 (base), Increased = 1, Decreased = 2
recode CANNAB_DURING (3=0 "No Change") (1=1 "Increased") (2=2 "Decreased"), gen(cannab_change)
```

```
recode CANNAB_DURING (3=0 "No Change") (1=1 "Increased") (2=2 "Decreased"), gen(cannab_change)
label define cannab 0 "No Change" 1 "Increased" 2 "Decreased", replace
label values cannab_change cannab

* Multinomial logistic regression
mlogit cannab_change i.dumYES_IPV i.age_group i.hhincome ///
       i.participant_education i.participant_employment ///
       i.has_children i.dumCaregiver, baseoutcome(0) vce(robust)

* Show relative risk ratios
mlogit, rrr

* Predicted probabilities of each category by IPV
margins dumYES_IPV, predict(outcome(0))   // No change
margins dumYES_IPV, predict(outcome(1))   // Increased
margins dumYES_IPV, predict(outcome(2))   // Decreased

* Differences between IPV and non-IPV
margins r.dumYES_IPV, predict(outcome(0))
margins r.dumYES_IPV, predict(outcome(1))
margins r.dumYES_IPV, predict(outcome(2))


*---------------------------------------------------------------------*
* OTHER SUBSTANCES
*---------------------------------------------------------------------*
recode SUBSTANCE_DURING (3=0 "No Change") (1=1 "Increased") (2=2 "Decreased"), gen(subst_change)
label define subst 0 "No Change" 1 "Increased" 2 "Decreased", replace
label values subst_change subst

mlogit subst_change i.dumYES_IPV i.age_group i.hhincome ///
       i.participant_education i.participant_employment ///
       i.has_children i.dumCaregiver, baseoutcome(0) vce(robust)
mlogit, rrr

margins dumYES_IPV, predict(outcome(0))
margins dumYES_IPV, predict(outcome(1))
margins dumYES_IPV, predict(outcome(2))

margins r.dumYES_IPV, predict(outcome(0))
margins r.dumYES_IPV, predict(outcome(1))
margins r.dumYES_IPV, predict(outcome(2))
```

```
*--------------------------------------------------------------------*
* TELEVISION
*--------------------------------------------------------------------*
recode TV_DURING (3=0 "No Change") (1=1 "Increased") (2=2 "Decreased"), gen(tv_change)
label define tv 0 "No Change" 1 "Increased" 2 "Decreased", replace
label values tv_change tv

mlogit tv_change i.dumYES_IPV i.age_group i.hhincome ///
       i.participant_education i.participant_employment ///
       i.has_children i.dumCaregiver, baseoutcome(0) vce(robust)
mlogit, rrr

margins dumYES_IPV, predict(outcome(0))
margins dumYES_IPV, predict(outcome(1))
margins dumYES_IPV, predict(outcome(2))

margins r.dumYES_IPV, predict(outcome(0))
margins r.dumYES_IPV, predict(outcome(1))
margins r.dumYES_IPV, predict(outcome(2))


*--------------------------------------------------------------------*
* INTERNET
*--------------------------------------------------------------------*
recode INTERNET_DURING (3=0 "No Change") (1=1 "Increased") (2=2 "Decreased"), gen(internet_change)
label define internet 0 "No Change" 1 "Increased" 2 "Decreased", replace
label values internet_change internet

mlogit internet_change i.dumYES_IPV i.age_group i.hhincome ///
       i.participant_education i.participant_employment ///
       i.has_children i.dumCaregiver, baseoutcome(0) vce(robust)
mlogit, rrr

margins dumYES_IPV, predict(outcome(0))
margins dumYES_IPV, predict(outcome(1))
margins dumYES_IPV, predict(outcome(2))

margins r.dumYES_IPV, predict(outcome(0))
margins r.dumYES_IPV, predict(outcome(1))
margins r.dumYES_IPV, predict(outcome(2))
```

```
margins r.dumYES_IPV, predict(outcome(2))


*------------------------------------------------------------------------*
* EXERCISE
*------------------------------------------------------------------------*
recode EXERCISING_DURING (3=0 "No Change") (1=1 "Increased") (2=2 "Decreased"), gen(exercise_change)
label define exercise 0 "No Change" 1 "Increased" 2 "Decreased", replace
label values exercise_change exercise

mlogit exercise_change i.dumYES_IPV i.age_group i.hhincome ///
       i.participant_education i.participant_employment ///
       i.has_children i.dumCaregiver, baseoutcome(0) vce(robust)
mlogit, rrr

margins dumYES_IPV, predict(outcome(0))
margins dumYES_IPV, predict(outcome(1))
margins dumYES_IPV, predict(outcome(2))

margins r.dumYES_IPV, predict(outcome(0))
margins r.dumYES_IPV, predict(outcome(1))
margins r.dumYES_IPV, predict(outcome(2))


*------------------------------------------------------------------------*
* JUNK FOOD
*------------------------------------------------------------------------*
recode EATJUNK_DURING (3=0 "No Change") (1=1 "Increased") (2=2 "Decreased"), gen(junk_change)
label define junk 0 "No Change" 1 "Increased" 2 "Decreased", replace
label values junk_change junk

mlogit junk_change i.dumYES_IPV i.age_group i.hhincome ///
       i.participant_education i.participant_employment ///
       i.has_children i.dumCaregiver, baseoutcome(0) vce(robust)
mlogit, rrr

margins dumYES_IPV, predict(outcome(0))
margins dumYES_IPV, predict(outcome(1))
margins dumYES_IPV, predict(outcome(2))

margins r.dumYES_IPV, predict(outcome(0))
margins r.dumYES_IPV, predict(outcome(1))
```

```
margins r.dumYES_IPV, predict(outcome(1))
margins r.dumYES_IPV, predict(outcome(2))


*--------------------------------------------------------------------*
* INFORMAL SUPPORT
*--------------------------------------------------------------------*
recode INFORMAL_DURING (3=0 "No Change") (1=1 "Increased") (2=2 "Decreased"), gen(informal_change)
label define informal 0 "No Change" 1 "Increased" 2 "Decreased", replace
label values informal_change informal

mlogit informal_change i.dumYES_IPV i.age_group i.hhincome ///
       i.participant_education i.participant_employment ///
       i.has_children i.dumCaregiver, baseoutcome(0) vce(robust)
mlogit, rrr

margins dumYES_IPV, predict(outcome(0))
margins dumYES_IPV, predict(outcome(1))
margins dumYES_IPV, predict(outcome(2))

margins r.dumYES_IPV, predict(outcome(0))
margins r.dumYES_IPV, predict(outcome(1))
margins r.dumYES_IPV, predict(outcome(2))


*--------------------------------------------------------------------*
* FORMAL SUPPORT
*--------------------------------------------------------------------*
recode FORMAL_DURING (3=0 "No Change") (1=1 "Increased") (2=2 "Decreased"), gen(formal_change)
label define formal 0 "No Change" 1 "Increased" 2 "Decreased", replace
label values formal_change formal

mlogit formal_change i.dumYES_IPV i.age_group i.hhincome ///
       i.participant_education i.participant_employment ///
       i.has_children i.dumCaregiver, baseoutcome(0) vce(robust)
mlogit, rrr

margins dumYES_IPV, predict(outcome(0))
margins dumYES_IPV, predict(outcome(1))
margins dumYES_IPV, predict(outcome(2))
```
