# Supplementary material for: “We were locked in with our trauma” - a mixed-methods study of health pathways among intimate partner violence (IPV) survivors during COVID-19 lockdowns in Ontario
Source: BMC Public Health. 2026 Jun 19;26:1924. doi: 10.1186/s12889-026-28032-6 (PMC13282876; doi:10.1186/s12889-026-28032-6)
Supplement: Supplementary file 5 — Additional file 5. Detailed Descriptive and Analytical Results Tables. [file 12889_2026_28032_MOESM5_ESM.docx]

**Additional File 5**

**Detailed Descriptive and Analytical Results Tables**

This supplement presents the full descriptive and analytical results of the study examining health inequities faced by survivors of IPV during COVID-19. To streamline the main text, detailed descriptive and analytical tables are included here rather than in the manuscript. Readers can refer to the tables below for comprehensive information.

The supplementary tables are as follows:

- **Table S1** presents a comprehensive comparison of sociodemographic, partner, and household characteristics between women who reported experiencing IPV (IPV group) and those who did not (non-IPV group) during the COVID-19 lockdown period. These comparisons were made using Chi-square or Fisher’s exact tests, depending on cell sizes, to assess statistically significant differences.
- **Table S2** examines differences in health-related behaviours—including substance use, screen time, physical activity, eating habits, sleep, and help-seeking—between IPV group and non-IPV group, using Chi-square tests with Bonferroni-adjusted p-values to account for multiple comparisons.
- **Table S3** reports results from multivariable logistic regression models predicting poor mental and physical health outcomes. The models account for a range of sociodemographic, behavioural, and contextual covariates to isolate the association between IPV during the pandemic and health outcomes. Only the significant findings are reported in the manuscript with the full model provided here in Table S4.

**Table S1: Descriptive Statistics Comparing Women Survivors of Intimate Partner Violence (IPV) and Non-Survivors in an Ontario Survey Cohort During COVID-19 Lockdowns (n=653)**

|  | **IPV group**  **(n=150; 23%)** | | **non-IPV group**  **Participants**  **(n=503; 77%)** | |  |  |  |
| --- | --- | --- | --- | --- | --- | --- | --- |
|  | **Mean (%)** | **n** | **Mean (%)** | **n** | ***χ²* (df)/ Fisher's Exact test (df)** | ***p-value*** |  |
| **Participant Characteristics** |  |  |  |  |  |  |  |
| Age (in years) |  | 150 |  | 502 |  |  |  |
| 18-34 | 51.33 | 77 | 20.12 | 101 | 76.67 (2) | 0.00 |  |
| 35-54 | 41.33 | 62 | 41.63 | 209 |  |  |  |
| 55 + | 7.33 | 11 | 38.25 | 192 |  |  |  |
| Relationship Status* |  | 149 |  | 503 |  |  |  |
| Heterosexual | 79.19 | 118 | 92.05 | 463 | n/a | 0.00 |  |
| LGBTQ2+ | 19.46 | 29 | 6.96 | 35 |  |  |  |
| Prefer not to say | 1.34 | 2 | 1.00 | 5 |  |  |  |
| Race |  | 150 |  | 503 |  |  |  |
| Racialized/Indigenous | 42.00 | 63 | 16.30 | 82 | 44.17 (2) | 0.000 |  |
| White | 56.00 | 84 | 80.72 | 406 |  |  |  |
| Prefer not to say | 2.00 | 3 | 2.98 | 15 |  |  |  |
| Immigration Status* |  | 150 |  | 503 |  |  |  |
| Non-immigrant | 89.33 | 134 | 94.23 | 474 | n/a | 0.069 |  |
| Immigrant & non-permanent resident | 8.67 | 13 | 5.17 | 26 |  |  |  |
| Don’t know | 2.00 | 3 | 0.60 | 3 |  |  |  |
| Education |  | 149 |  | 502 |  |  |  |
| Primary | 18.79 | 28 | 16.53 | 83 | 0.5837 (2) | 0.747 |  |
| Trade/Diploma | 37.58 | 56 | 36.85 | 185 |  |  |  |
| University | 43.62 | 65 | 46.61 | 234 |  |  |  |
| Employment |  | 150 |  | 503 |  |  |  |
| Employed | 64.00 | 96 | 67.99 | 342 | 0.8338 (2) | 0.361 |  |
| Unemployed | 36.00 | 54 | 32.01 | 161 |  |  |  |
| **Partner Characteristics** |  |  |  |  |  |  |  |
| Gender* |  | 149 | 503 |  |  |  |  |
| Woman | 18.12 | 27 | 6.96 | 35 | n/a | 0.000 |  |
| Man | 79.19 | 118 | 92.05 | 463 |  |  |  |
| Gender Diverse | 2.68 | 4 | 0.99 | 5 |  |  |  |
| Age (in years) |  | 147 |  | 500 |  |  |  |
| 18-34 | 46.94 | 69 | 16.80 | 84 | 88.87 (2) | 0.000 |  |
| 35-54 | 46.26 | 68 | 39.40 | 197 |  |  |  |
| 55 + | 6.80 | 10 | 43.80 | 219 |  |  |  |
| Race* |  | 149 |  | 501 |  |  |  |
| Racialized/Indigenous | 37.58 | 56 | 17.37 | 87 | n/a | 0.000 |  |
| White | 59.73 | 89 | 79.64 | 399 |  |  |  |
| Prefer not to say | 2.68 | 4 | 2.99 | 15 |  |  |  |
|  |  |  |  |  |  |  |  |
| Immigration Status* |  | 150 |  | 503 |  |  |  |
| Non-immigrant | 83.78 | 124 | 94.00 | 470 | n/a | 0.000 |  |
| Immigrant & non-permanent resident | 16.22 | 24 | 6.00 | 30 |  |  |  |
| Don’t know | 1.33 | 2 | 0.60 | 3 |  |  |  |
| Education |  | 148 |  | 499 |  |  |  |
| Primary | 33.78 | 50 | 25.65 | 128 | 6.48 (2) | 0.039 |  |
| Trade/Diploma | 30.41 | 45 | 41.28 | 206 |  |  |  |
| University | 35.81 | 53 | 33.07 | 165 |  |  |  |
| Employment |  | 148 |  | 499 |  |  |  |
| Employed | 63.51 | 94 | 70.74 | 353 | 2.79 (1) | 0.095 |  |
| Unemployed | 36.49 | 54 | 29.26 | 146 |  |  |  |
| **Household Income** |  | 142 |  | 450 |  |  |  |
| <$40,0000 | 41.55 | 59 | 14.44 | 65 | 51.79 (3) | 0.000 |  |
| $40,000-$69,999 | 19.01 | 27 | 24.44 | 110 |  |  |  |
| $70,0100-$99,999 | 19.72 | 28 | 21.33 | 96 |  |  |  |
| $100,000 + | 19.72 | 28 | 39.78 | 179 |  |  |  |
| **Geography**** |  | 150 |  | 503 |  |  |  |
| Eastern Ontario | 19.33 | 29 | 10.34 | 52 | 51.79 (4) | 0.007 |  |
| Central Ontario | 11.33 | 17 | 8.95 | 45 |  |  |  |
| Toronto and the GTA (905 Belt) | 38.00 | 57 | 45.53 | 229 |  |  |  |
| Western Ontario | 20.67 | 31 | 28.23 | 142 |  |  |  |
| Northern Ontario | 10.67 | 16 | 6.96 | 35 |  |  |  |
| **Urban/Rural** |  | 141 |  | 483 |  |  |  |
| Rural | 12.06 | 17 | 13.25 | 64 | 0.1377 (1) | 0.711 |  |
| Urban | 87.94 | 124 | 86.75 | 419 |  |  |  |
| **Community violence is a problem** |  | 150 |  | 501 |  |  |  |
| No | 58.00 | 87 | 69.46 | 364 | 11.65 (1) | 0.001 |  |
| Yes | 42.00 | 63 | 27.35 | 137 |  |  |  |
| **Enough information about services available during pandemic** |  | 150 |  | 501 |  |  |  |
| Yes | 46.98 | 70 | 70.82 | 352 | 28.77 (1) | 0.000 |  |
| No | 53.02 | 79 | 29.18 | 145 |  |  |  |
| **Impact of substance use on relationship** |  | 150 |  | 503 |  |  |  |
| Moderate/High impact | 62.00 | 93 | 10.54 | 53 | 176.28 (1) | 0.000 |  |
| Low impact | 38.00 | 57 | 89.46 | 450 |  |  |  |
| **Number of Children** |  |  |  |  |  |  |  |
| No children | 45.33 | 68 | 65.67 | 329 | 20.06 (1) | 0.000 |  |
| Yes Children | 54.66 | 82 | 34.33 | 172 |  |  |  |
| **Informal caregiver** |  | 150 |  | 503 |  |  |  |
| Yes | 60.00 | 90 | 44.53 | 224 | 11.07 (1) | 0.001 |  |
| No | 40.00 | 60 | 55.47 | 279 |  |  |  |
| *** *Fisher’s exact test was used due to small cell counts in categorical variables, ensuring appropriate statistical inference when expected frequencies were low.  **Post-hoc tests revealed that Eastern Ontario (higher proportion of IPV survivors) and Wester Ontario (lower proportion of reported IPV) were significantly different. | | | | | | | |

**Table S2: Health Behaviors Among Women Experiencing IPV and Non-IPV During COVID-19 Lockdowns: Chi-Squared Analysis (n=653)**

|  | **Quantitative** | | | **Pairwise comparison Bonferroni Correction*** |
| --- | --- | --- | --- | --- |
| **Variable** | **IPV group**  **n (%)** | **Non-IPV group**  **n (%)** | *χ²* **(df)/ Fisher's Exact test (df)** | **p-value** |
| **SUBSTANCE USE** |  |  |  |  |
| **Alcohol Consumption** (n=649) | (n=149) | (n=500) | 52.24 (2)  p < 0.001 |  |
| Increased | 69 (46.3%) | 103 (20.6%) |  | **<0.001** |
| Decreased | 25 (16.8%) | 51 (10.2%) |  | 0.028 |
| No Change | 55 (36.9%) | 346 (69.2%) |  | **<0.001** |
| **Tobacco Use** (n=642) | (n=148) | (n=494) | 46.35 (2)  p < 0.001 |  |
| Increased | 33 (22.3%) | 33 (6.7%) |  | **<0.001** |
| Decreased | 14 (9.5%) | 13 (2.6%) |  | **0.0003** |
| No Change | 101 (68.2%) | 448 (90.7%) |  | **<0.001** |
| **Cannabis Use** (n=643) | (n=148) | (n=495) | 67.62 (2)  p < 0.001 |  |
| Increased | 45 (30.4%) | 49 (9.9%) |  | **<0.001** |
| Decreased | 15 (10.1%) | 8 (1.6%) |  | **<0.001** |
| No Change | 88 (59.5%) | 438 (88.5%) |  | **<0.001** |
| **Illicit Substance Use** (n=642) | (n=146) | (n=496) | 68.10 (2)  p < 0.001 |  |
| Increased | 17 (11.6%) | 2 (0.4%) |  | **<0.001** |
| Decreased | 10 (6.8%) | 5 (1.0%) |  | **<0.001** |
| No Change | 119 (81.5%) | 489 (98.6%) |  | **<0.001** |
|  |  |  |  |  |
| **TV AND INTERNET HABITS** |  |  |  |  |
| **TV Watching** (n=653) | (n=150) | (n=503) | 8.95 (2)  p = 0.011 |  |
| Increased | 109 (72.7%) | 359 (71.4%) |  | 0.757 |
| Decreased | 17 (11.3%) | 27 (5.4%) |  | **0.011** |
| No Change | 24 (16.0%) | 117 (23.3%) |  | 0.058 |
| **Internet Use** (n=652) | (n=149) | (n=503) | 16.12 (2)  p < 0.001 |  |
| Increased | 119 (79.9%) | 381 (75.7%) |  | 0.296 |
| Decreased | 13 (8.7%) | 14 (2.8%) |  | **0.0014** |
| No Change | 17 (11.4%) | 108 (21.5%) |  | **0.006** |
|  |  |  |  |  |
| **EXERCISE, DIET AND SLEEP HABITS** |  |  |  |  |
| **Exercise** (n=652) | (n=150) | (n=502) | 6.38 (2)  p = 0.041 |  |
| Increased | 41 (27.3%) | 132 (26.3%) |  | 0.8 |
| Decreased | 68 (45.3%) | 180 (35.9%) |  | **0.036** |
| No Change | 41 (27.3%) | 190 (37.8%) |  | **0.018** |
| **Eating Junk Food** (n=652) | (n=150) | (n=502) | 13.48 (2)  p = 0.001 |  |
| Increased | 84 (56.0%) | 218 (43.4%) |  | **0.007** |
| Decreased | 21 (14.0%) | 49 (9.8%) |  | 0.141 |
| No Change | 45 (30.0%) | 235 (46.8%) |  | **0.0003** |
| **Sleep Quality** (n=650) | (n=149) | (n=501) | 52.49 (2)  p < 0.001 |  |
| Increased | 29 (19.3%) | 77 (15.4%) |  | 0.235 |
| Decreased | 97 (64.7%) | 184 (36.7%) |  | **<0.001** |
| No Change | 23 (15.3%) | 240 (47.9%) |  | **<0.001** |
|  |  |  |  |  |
| **HELP SEEKING HABITS** |  |  |  |  |
| **Informal Help-Seeking** (n=653) | (n=150) | (n=503) | 48.62 (2)  p < 0.001 |  |
| Increased | 44 (29.3%) | 106 (21.1%) |  | 0.035 |
| Decreased | 54 (36.0%) | 73 (14.5%) |  | **<0.001** |
| No Change | 52 (34.7%) | 324 (64.4%) |  | **<0.001** |
| **Formal Help-Seeking** (n=652) | (n=150) | (n=502) | 78.71 (2)  p < 0.001 |  |
| Increased | 50 (33.3%) | 63 (12.6%) |  | **<0.001** |
| Decreased | 44 (29.3%) | 57 (11.4%) |  | **<0.001** |
| No Change | 56 (37.3%) | 382 (76.1%) |  | **<0.001** |

Notes: * Pairwise comparisons between IPV and non-IPV groups were conducted for each categorical response (e.g., increased, decreased, no change); p-values were adjusted using the Bonferroni correction to account for multiple comparisons, maintaining a conservative threshold for statistical significance.

**Table S3: Logistic Regression Models Comparing Poor Mental and Physical Health Outcomes Between IPV Survivors and Non-Survivors Survey Respondents During COVID-19 Lockdowns in Ontario**

|  | **Poor Mental Health during COVID-19 lockdowns** | | | **Poor Physical Health during COVID-19 lockdowns** | | |
| --- | --- | --- | --- | --- | --- | --- |
| **Variables (reference group)** | **aOdds Ratio** | **95% CI** | **p-value** | **aOdds Ratio** | **95% CI** | **p-value** |
|  |  |  |  |  |  |  |
| **IPV during COVID-19 Lockdowns** | 2.460** | 1.273 - 4.753 | 0.007 | 1.835* | 1.003 - 3.359 | 0.049 |
|  |  |  |  |  |  |  |
| **Participant Characteristics** |  |  |  |  |  |  |
| **Age (55+)** |  |  |  |  |  |  |
| 18-34 | 1.174 | 0.387 - 3.566 | 0.777 | 1.600 | 0.545 - 4.693 | 0.392 |
| 35-54 | 1.300 | 0.569 - 2.971 | 0.534 | 1.657 | 0.717 - 3.825 | 0.237 |
| **Race (white)** |  |  |  |  |  |  |
| Racialized, Indigenous, prefer not to say | 0.626 | 0.309 - 1.270 | 0.194 | 0.521 | 0.250 - 1.085 | 0.082 |
| **Immigration Status (non-immigrant)** |  |  |  |  |  |  |
| Immigrant & non-permanent resident | 0.819 | 0.294 - 2.281 | 0.703 | 1.257 | 0.421 - 3.757 | 0.682 |
| **Education (primary)** |  |  |  |  |  |  |
| Trade/Diploma | 0.926 | 0.500 - 1.716 | 0.808 | 0.879 | 0.454 - 1.704 | 0.703 |
| University | 0.986 | 0.514 - 1.891 | 0.967 | 1.281 | 0.636 - 2.580 | 0.489 |
| **Employment status (employed)** |  |  |  |  |  |  |
| Unemployed | 0.665 | 0.409 - 1.082 | 0.101 | 1.181 | 0.712 - 1.957 | 0.519 |
| **Partner Characteristics** |  |  |  |  |  |  |
| Gender (Woman/Gender Diverse) (1) |  |  |  |  |  |  |
| Man | 2.283* | 1.150 - 4.532 | 0.018 | 0.606 | 0.303 - 1.211 | 0.156 |
| **Age (55+)** |  |  |  |  |  |  |
| 18-34 | 1.028 | 0.334 - 3.160 | 0.962 | 0.563 | 0.191 - 1.659 | 0.297 |
| 35-54 | 0.919 | 0.410 - 2.059 | 0.837 | 0.736 | 0.320 - 1.690 | 0.469 |
| **Race (white)** |  |  |  |  |  |  |
| Racialized, Indigenous, prefer not to say (2) | 1.002 | 0.508 - 1.977 | 0.995 | 0.755 | 0.375 - 1.522 | 0.432 |
| **Immigration Status (non-immigrant)** |  |  |  |  |  |  |
| Immigrant & non-permanent resident | 1.825 | 0.753 - 4.424 | 0.183 | 0.613 | 0.257 - 1.461 | 0.269 |
| **Education (primary)** |  |  |  |  |  |  |
| Trade/Diploma | 0.673 | 0.395 - 1.147 | 0.145 | 1.295 | 0.742 - 2.263 | 0.363 |
| University | 0.854 | 0.459 - 1.589 | 0.619 | 0.888 | 0.459 - 1.718 | 0.725 |
| **Employment status (employed)** |  |  |  |  |  |  |
| Unemployed | 0.845 | 0.514 - 1.387 | 0.505 | 0.946 | 0.559 - 1.601 | 0.835 |
| **Household Income ($100,000 +)** |  |  |  |  |  |  |
| <$40,0000 | 0.872 | 0.450 - 1.689 | 0.685 | 1.344 | 0.676 - 2.673 | 0.399 |
| $40,000-$69,999 | 0.667 | 0.376 - 1.185 | 0.167 | 1.150 | 0.611 - 2.166 | 0.665 |
| $70,0100-$99,999 | 0.731 | 0.424 - 1.262 | 0.261 | 0.906 | 0.507 - 1.617 | 0.738 |
| **Geography (Toronto/GTA)** |  |  |  |  |  |  |
| Eastern Ontario | 0.744 | 0.385 - 1.437 | 0.378 | 1.128 | 0.579 - 2.198 | 0.723 |
| Central Ontario | 1.630 | 0.773 - 3.436 | 0.199 | 0.669 | 0.311 - 1.441 | 0.305 |
| Western Ontario | 1.118 | 0.681 - 1.835 | 0.660 | 0.677 | 0.399 - 1.149 | 0.148 |
| Northern Ontario | 1.180 | 0.540 - 2.579 | 0.678 | 0.581 | 0.252 - 1.342 | 0.204 |
| **Community violence is a problem (No) (3)** |  |  |  |  |  |  |
| Yes | 1.074 | 0.685 - 1.681 | 0.757 | 1.432 | 0.912 - 2.247 | 0.119 |
| **Enough information about services available during pandemic (Yes)** |  |  |  |  |  |  |
| No | 1.310 | 0.831 - 2.066 | 0.245 | 1.733* | 1.107 - 2.712 | 0.016 |
| **Impact of substance use on relationship (Low)** |  |  |  |  |  |  |
| Moderate/High | 0.695 | 0.380 - 1.272 | 0.238 | 0.925 | 0.512 - 1.669 | 0.795 |
| **Have children (No) (4)** |  |  |  |  |  |  |
| Yes | 0.860 | 0.452 - 1.638 | 0.647 | 0.789 | 0.405 - 1.539 | 0.487 |
| **Informal caregiver (no)** |  |  |  |  |  |  |
| Yes | 1.337 | 0.771 - 2.319 | 0.301 | 1.384 | 0.769 - 2.491 | 0.279 |
| **Help seeking (increased/no change)** |  |  |  |  |  |  |
| Formal decreased | 0.726 | 0.370 - 1.424 | 0.352 | 0.885 | 0.461 - 1.698 | 0.712 |
| Informal decreased | 3.222*** | 1.701 - 6.106 | 0.00 | 1.367 | 0.770 - 2.427 | 0.286 |
| **Health outcomes (Good)** |  |  |  |  |  |  |
| Poor Physical | 6.997*** | 4.245 - 11.53 | 0.00 |  |  |  |
| Poor Mental |  |  |  | 7.056*** | 4.272 - 11.66 | 0.000 |
| **Constant** | 0.406 | 0.143 - 1.153 | 0.0904 | 0.123*** | 0.038 - 0.397 | 0.000 |
|  |  |  |  |  |  |  |
| **Observations** | 559 |  |  | 559 |  |  |
| *** p<0.001, ** p<0.01, * p<0.05  Notes: | | | | | | |
