# Supplementary material for: “We were locked in with our trauma” - a mixed-methods study of health pathways among intimate partner violence (IPV) survivors during COVID-19 lockdowns in Ontario
Source: BMC Public Health. 2026 Jun 19;26:1924. doi: 10.1186/s12889-026-28032-6 (PMC13282876; doi:10.1186/s12889-026-28032-6)
Supplement: Supplementary file 6 — Additional file 6. Propensity Score Matching Sensitivity Analysis. [file 12889_2026_28032_MOESM6_ESM.docx]

**Additional File 6**

**Propensity Score Matching Sensitivity Analysis**

**Overview**

A reviewer raised the important question of whether differences in health outcomes between women who experienced intimate partner violence (IPV) during COVID-19 lockdowns and those who did not might be explained by pre-existing differences between the two groups — for example, that IPV-exposed women were already younger, lower-income, or more marginalized before the lockdowns began.

To address this, a propensity score matching analysis was conducted. This technique creates a comparison group of non-IPV women who are statistically as similar as possible to IPV-exposed women across a wide range of background characteristics. The idea is straightforward: if the health differences hold up even after we have carefully matched the two groups on these characteristics, we can be more confident that IPV itself — not background differences — is driving the findings.

**Who was matched and who was not**

Of the 150 IPV-exposed women in the women-only analytic sample, 76 were successfully matched to a non-IPV woman with a very similar background profile (caliper = 0.05, nearest-neighbour matching without replacement). There were 56 IPV-exposed women could not be matched because they had no comparable counterpart in the non-IPV group — they were the most marginalized women in the sample (youngest, lowest income, highest rates of partner substance use). This is itself a meaningful finding: the most vulnerable women fall outside the range of comparison, which highlights the limits of matching in a study of inequality. A total of 19 **women with missing values on one or more of the matching covariates**.

Among IPV-exposed women, the 56 who could not be matched were significantly more likely to be aged 18–34 (70% vs. 40%, p = .001), to have a household income below $40,000 (59% vs. 30%, p = .001), to identify as racialized or Indigenous (55% vs. 37%, p = .031), and to have a partner with moderate or high substance use (98% vs. 40%, p < .001), compared to the 76 who were successfully matched. This pattern suggests that the women excluded from the matched analysis were among the most structurally marginalized in the sample, and that propensity score matching — by design — cannot capture the experiences of those at the extreme end of vulnerability. This is an important limitation of the method in the context of health equity research.

**Table: Characteristics of Matched vs. Unmatched IPV-Exposed Women**

Among the 132 IPV-exposed women in the analytic sample, 76 were successfully matched to a non-IPV comparator and 56 could not be matched. The table below compares these two groups on key sociodemographic characteristics.

| **Characteristic** | **Matched IPV (n = 76)** | **Unmatched IPV (n = 56)** | **t** | **p-value** |
| --- | --- | --- | --- | --- |
| Age 18–34 | 40% | 70% | −3.59 | **.001** |
| Household income below $40,000 | 30% | 59% | −3.51 | **.001** |
| Partner with moderate/high substance use | 40% | 98% | −8.57 | **<.001** |
| Racialized or Indigenous identity | 37% | 55% | −2.18 | **.031** |
| Has children | 49% | 64% | −1.83 | .069 |
| Immigrant, PR, or refugee | 8% | 11% | −0.62 | .534 |
| Informal caregiver | 56% | 66% | −1.17 | .244 |

Note: Values represent the percentage of women in each group with the characteristic. Bold p-values indicate statistically significant differences (p < .05). Two-sample t-tests with equal variances. Matched = IPV-exposed women successfully matched to a non-IPV comparator (on common support). Unmatched = IPV-exposed women who fell outside the common support region and could not be matched.

**Table: Characteristics of Matched vs. Unmatched IPV-Exposed Women**

Among the 132 IPV-exposed women in the analytic sample, 76 were successfully matched to a non-IPV comparator and 56 could not be matched. The table below compares these two groups on key sociodemographic characteristics.

| **Characteristic** | **Matched IPV (n = 76)** | **Unmatched IPV (n = 56)** | **t** | **p-value** |
| --- | --- | --- | --- | --- |
| Age 18–34 | 40% | 70% | −3.59 | **.001** |
| Household income below $40,000 | 30% | 59% | −3.51 | **.001** |
| Partner with moderate/high substance use | 40% | 98% | −8.57 | **<.001** |
| Racialized or Indigenous identity | 37% | 55% | −2.18 | **.031** |
| Has children | 49% | 64% | −1.83 | .069 |
| Immigrant, PR, or refugee | 8% | 11% | −0.62 | .534 |
| Informal caregiver | 56% | 66% | −1.17 | .244 |

Note: Values represent the percentage of women in each group with the characteristic. Bold p-values indicate statistically significant differences (p < .05). Two-sample t-tests with equal variances. Matched = IPV-exposed women successfully matched to a non-IPV comparator (on common support). Unmatched = IPV-exposed women who fell outside the common support region and could not be matched.

**Table S-A: How similar were the two groups before and after matching?**

The table below shows whether IPV-exposed and non-IPV women differed on 13 background characteristics before matching (Unmatched) and after matching (Matched). The '% Bias' column shows how large the difference between groups was — values below 10% are generally considered acceptable. The p-value column shows whether any remaining difference was statistically significant.

| **Characteristic** | **Sample** | **IPV** | **Non-IPV** | **% Bias** | **t-test** | |
| --- | --- | --- | --- | --- | --- | --- |
|  |  |  |  |  | **t** | **p-value** |
| Age 18–34 | Unmatched | .508 | .191 | 70.3 | 7.56 | **0.000** |
|  | Matched | .368 | .421 | −11.7 | −0.66 | 0.510 |
| Age 35–54 | Unmatched | .417 | .416 | 0.1 | 0.01 | 0.994 |
|  | Matched | .526 | .566 | −8.0 | −0.49 | 0.628 |
| Racialized/Indigenous | Unmatched | .439 | .188 | 56.0 | 6.02 | **0.000** |
|  | Matched | .355 | .382 | −5.9 | −0.33 | 0.739 |
| Immigrant/PR/Refugee | Unmatched | .098 | .049 | 19.0 | 2.10 | **0.036** |
|  | Matched | .092 | .092 | 0.0 | 0.00 | 1.000 |
| Trade/College education | Unmatched | .371 | .377 | −1.1 | −0.11 | 0.909 |
|  | Matched | .355 | .355 | 0.0 | 0.00 | 1.000 |
| University education | Unmatched | .462 | .453 | 1.7 | 0.17 | 0.862 |
|  | Matched | .487 | .487 | 0.0 | 0.00 | 1.000 |
| Unemployed/Retired | Unmatched | .348 | .319 | 6.3 | 0.64 | 0.522 |
|  | Matched | .289 | .197 | 19.5 | 1.32 | 0.188 |
| Household income <$40K | Unmatched | .409 | .151 | 59.8 | 6.57 | **0.000** |
|  | Matched | .276 | .237 | 9.2 | 0.55 | 0.580 |
| Community violence | Unmatched | .417 | .284 | 28.1 | 2.89 | **0.004** |
|  | Matched | .329 | .316 | 2.8 | 0.17 | 0.863 |
| Informed about services | Unmatched | .492 | .705 | −44.2 | −4.56 | **0.000** |
|  | Matched | .566 | .592 | −5.5 | −0.33 | 0.744 |
| Partner substance use (moderate/high) | Unmatched | .636 | .102 | 132.4 | 15.18 | **0.000** |
|  | Matched | .382 | .355 | 6.5 | 0.33 | 0.739 |
| Has children | Unmatched | .561 | .342 | 44.9 | 4.58 | **0.000** |
|  | Matched | .500 | .553 | −10.8 | −0.65 | 0.519 |
| Informal caregiver | Unmatched | .614 | .442 | 34.8 | 3.49 | **0.001** |
|  | Matched | .579 | .658 | −16.0 | −1.00 | 0.320 |
| **Overall Model Fit** | | | | | | |
| **Sample** | **Pseudo R²** | **LR chi²** | **p > chi²** | **Mean % Bias** | **Median % Bias** | |
| Unmatched | 0.370 | 226.39 | **0.000** | 38.4 | 34.8 | |
| Matched | 0.046 | 9.63 | 0.724 | 7.4 | 6.5 | |

Before matching, the two groups differed substantially on many characteristics (mean bias = 38.4%). After matching, these differences were greatly reduced (mean bias = 7.4%), and none of the individual characteristics remained statistically significant. The overall test of whether the groups were comparable after matching returned p = 0.724, confirming that balance was achieved.

**Table S-B: Mental and physical health outcomes in the matched sample**

The two models below repeat the primary logistic regression analyses (Table 4 in the main manuscript) using only the 152 matched women (76 IPV-exposed, 76 non-IPV). Only key significant and near-significant predictors are shown for readability; full model output is available from the corresponding author on request.

| **Variable** | **Odds Ratio** | **95% CI** | **p-value** |
| --- | --- | --- | --- |
| **OUTCOME: Poor Mental Health (matched sample, n=151; OR=3.92, p=.020)** | | | |
| IPV exposure (Yes vs. No) | 3.92 | 1.24, 12.37 | **.020** |
| Decreased informal help-seeking | 4.63 | 1.05, 20.39 | **.043** |
| Poor physical health | 13.81 | 3.28, 58.09 | <.001 |
| Immigrant/PR/Refugee (participant) | 10.44 | 1.04, 104.92 | **.046** |
| Unemployed/Retired | 0.27 | 0.08, 0.92 | **.037** |
| Eastern Ontario (vs. Toronto) | 0.04 | 0.006, 0.27 | **.001** |
| **OUTCOME: Poor Physical Health (matched sample, n=151; OR=1.69, p=.284)** | | | |
| IPV exposure (Yes vs. No) | 1.69 | 0.65, 4.40 | .284 |
| Household income <$40K | 12.71 | 2.03, 79.39 | **.007** |
| Caregiving responsibilities | 14.07 | 1.82, 108.86 | **.011** |
| Community violence exposure | 3.40 | 1.06, 10.93 | **.040** |
| Lack of information about services | 4.06 | 1.37, 12.06 | **.012** |
| Eastern Ontario (vs. Toronto) | 6.80 | 1.16, 39.82 | **.034** |
| Poor mental health | 13.25 | 3.22, 54.61 | <.001 |

**What the mental and physical health results mean**

Mental health: The matched analysis confirmed and strengthened the main finding. After matching the two groups on background characteristics, IPV-exposed women still had nearly four times the odds of reporting poor mental health compared to non-IPV women (OR = 3.92, p = .020). This is stronger than the original finding (OR = 2.46), suggesting the main analysis was not inflated by group differences.

Physical health: In the matched sample, the association between IPV and poor physical health was no longer statistically significant (OR = 1.69, p = .284). This is a more modest finding than in the original analysis (OR = 1.84, p = .049), and the change is most likely explained by reduced statistical power in the smaller matched sample (n = 152 vs. n = 653) rather than by the absence of a true effect. The direction of the association remained the same (IPV women still had higher odds), and the original finding was already borderline. This result should be interpreted with caution.

**Table S-C: Behavioural pathway outcomes in the matched sample**

The table below repeats the behavioural pathway analyses (Table 3 in the main manuscript) in the matched sample (n = 152). For each behaviour, results are shown for three possible directions of change: no change (the reference category), increased, and decreased during lockdown.

The Relative Risk Ratio (RRR) compares how likely IPV-exposed women were to shift in a given direction (increased or decreased) rather than show no change, relative to non-IPV women. A value above 1.0 means IPV-exposed women were more likely to change in that direction; below 1.0 means less likely. The predicted probabilities show the estimated percentage of each group falling into each category; the difference column shows the gap between groups in plain percentage-point terms.

| **Behaviour** | **Outcome** | **Relative Risk Ratio** | | | **Predicted Probabilities** | | | |
| --- | --- | --- | --- | --- | --- | --- | --- | --- |
|  |  | **RRR** | **95% CI** | **p** | **Non-IPV** | **IPV** | **Difference** | **p** |
| **Alcohol** | No change | — | — | — | .591 | .475 | −.116 | .138 |
|  | Increased | 1.33 | 0.62, 2.84 | .465 | .321 | .337 | .016 | .831 |
|  | Decreased | 2.88 | 0.98, 8.40 | .053 | .088 | .188 | .099 | .071 |
| **Tobacco** | No change | — | — | — | .808 | .798 | −.011 | .863 |
|  | Increased | 0.90 | 0.33, 2.46 | .844 | .138 | .124 | −.014 | .795 |
|  | Decreased | 1.48 | 0.37, 5.96 | .580 | .054 | .078 | .024 | .561 |
| **Cannabis** | No change | — | — | — | .841 | .680 | −.161 | **.012** |
|  | Increased | 2.65 | 1.07, 6.57 | **.035** | .135 | .249 | .113 | .052 |
|  | Decreased† | — | — | — | .023 | .071 | .048 | .160 |
| **Illicit substances†** | No change | — | — | — | .983 | .917 | −.065 | — |
|  | Increased | — | — | — | .000 | .035 | .035 | — |
|  | Decreased | — | — | — | .017 | .047 | .030 | — |
| **Television** | No change | — | — | — | .204 | .203 | −.001 | .983 |
|  | Increased | 0.97 | 0.38, 2.49 | .948 | .740 | .702 | −.038 | .607 |
|  | Decreased | 1.82 | 0.37, 9.02 | .462 | .056 | .095 | .040 | .377 |
| **Internet†** | No change | — | — | — | .204 | .163 | −.041 | — |
|  | Increased | — | — | — | .789 | .756 | −.033 | — |
|  | Decreased | — | — | — | .007 | .081 | .075 | — |
| **Exercise** | No change | — | — | — | .330 | .277 | −.053 | .469 |
|  | Increased | 0.85 | 0.36, 2.03 | .715 | .356 | .250 | −.107 | .145 |
|  | Decreased | 1.95 | 0.83, 4.60 | .127 | .314 | .474 | .160 | **.034** |
| **Junk food** | No change | — | — | — | .304 | .369 | .065 | .376 |
|  | Increased | 0.78 | 0.36, 1.67 | .516 | .534 | .527 | −.007 | .934 |
|  | Decreased | 0.46 | 0.14, 1.54 | .208 | .163 | .104 | −.058 | .267 |
| **Informal support** | No change | — | — | — | .564 | .397 | −.167 | **.038** |
|  | Increased | 1.93 | 0.85, 4.41 | .118 | .246 | .318 | .073 | .312 |
|  | Decreased | 2.28 | 0.94, 5.50 | .067 | .191 | .285 | .094 | .162 |
| **Formal support** | No change | — | — | — | .751 | .439 | −.313 | **<.001** |
|  | Increased | 6.18 | 2.44, 15.66 | **<.001** | .111 | .367 | .257 | **<.001** |
|  | Decreased | 2.59 | 0.97, 6.92 | .057 | .138 | .194 | .056 | .354 |
| **Sleep** | No change | — | — | — | .385 | .207 | −.178 | **.015** |
|  | Increased | 2.24 | 0.76, 6.61 | .145 | .159 | .173 | .014 | .811 |
|  | Decreased | 2.74 | 1.19, 6.34 | **.018** | .456 | .620 | .164 | **.044** |

Note: RRR = relative risk ratio; CI = confidence interval. Reference category = No Change. All models adjusted for age, household income, education, employment, children, and caregiving. Bold values indicate p < 0.05. Differences reflect IPV minus non-IPV predicted probabilities. † Illicit substances and internet models produced unreliable estimates due to very sparse outcome categories in the small matched sample; RRR and standard errors are not reported. Predicted probabilities are shown for descriptive purposes only.

**What the behavioural results mean**

The behavioural findings were largely consistent with the original analysis, with several findings holding up and some becoming clearer after matching:

• Formal support-seeking: The strongest and most consistent finding across both the original and matched analyses. IPV-exposed women were over six times more likely to have increased their use of formal support services (shelters, crisis lines, counselling) compared to non-IPV women (RRR = 6.18, p < .001). In real terms, 37% of IPV-exposed women increased formal support-seeking versus 11% of non-IPV women — a 26 percentage point difference. This finding is robust.

• Sleep disruption: IPV-exposed women were nearly three times more likely to experience decreased sleep (RRR = 2.74, p = .018). In the matched sample, 62% of IPV-exposed women reported worse sleep compared to 46% of non-IPV women.

• Cannabis use: IPV-exposed women were more than twice as likely to have increased cannabis use (RRR = 2.65, p = .035) — a finding consistent with the original analysis.

• Exercise: IPV-exposed women were more likely to have decreased their physical activity, with a 16 percentage point gap in the predicted probability of reduced exercise (p = .034).

• Informal support-seeking: IPV-exposed women were significantly less likely to report no change in informal support — meaning they were more likely to have experienced a shift (either increase or decrease) in reaching out to family and friends (p = .038 for the no-change contrast).

• Alcohol, tobacco, television, junk food: No significant differences between groups were found in the matched sample, consistent with the original analysis.

• Illicit substances and internet: These models could not produce reliable estimates in the matched sample due to very small numbers of women reporting changes in these behaviours. Results are shown descriptively only.

Overall, the matched analyses support the conclusion that IPV exposure during COVID-19 lockdowns was associated with distinct behavioural, psychological, and physiological health patterns that are not explained by pre-existing demographic differences between groups.
